# Systematic Investigation of the Diagnostic and Prognostic Impact of LINC01087 in Human Cancers

**DOI:** 10.3390/cancers14235980

**Published:** 2022-12-03

**Authors:** Fatima Domenica Elisa De Palma, Vincent Carbonnier, Francesco Salvatore, Guido Kroemer, Jonathan G. Pol, Maria Chiara Maiuri

**Affiliations:** 1 Department of Molecular Medicine and Medical Biotechnologies, University of Napoli Federico II, 80131 Napoli, Italy; 2 CEINGE-Biotecnologie Avanzate Franco Salvatore, 80145 Napoli, Italy; 3 Équipe Labellisée par la Ligue Contre le Cancer, Centre de Recherche des Cordeliers, Inserm U1138, Université Paris Cité, Sorbonne Université, Institut Universitaire de France, 75005 Paris, France; 4 Metabolomics and Cell Biology Platforms, Gustave Roussy Cancer Campus, 94805 Villejuif, France; 5 Centro Interuniversitario per Malattie Multigeniche e Multifattoriali e Loro Modelli Animali (Federico II, 80131, Napoli, Tor Vergata, Rome and “G. D’Annunzio”, Chieti-Pescara), 80131 Napoli, Italy; 6 Department of Biology, Institut du Cancer Paris CARPEM, Hôpital Européen Georges Pompidou, 75004 Paris, France

**Keywords:** LINC01087, long non-coding RNA, biomarker, diagnostic biomarker, prognostic biomarker, stomach cancer, breast cancer, testicular cancer, epigenetics, lung squamous cell carcinoma, ovarian cancer, esophageal cancer, kidney papillary cell carcinoma, ion channel, peptide receptor

## Abstract

**Simple Summary:**

LINC01087 is a recently described long non-coding RNA. Its potential implication within oncology is being increasingly acknowledged. Here, we explored the clinical interest of LINC01087 in the diagnosis and prognostication of multiple cancer types. A series of in-depth in silico analyses revealed LINC01087 as a potential diagnostic indicator of some hormone-related tumors, including breast cancer (BC), ovarian carcinoma, and testicular germ cell tumors, as well as other cancer histotypes, such as esophageal and stomach cancers. Moreover, LINC01087 appears as a prognostic indicator in BC and renal papillary cell carcinoma. Therefore, the quantitation of LINC01087 in tissue specimens might have a clinical utility to improve the management of these pathologies in the future.

**Abstract:**

(1) Background: Long non-coding RNAs may constitute epigenetic biomarkers for the diagnosis, prognosis, and therapeutic response of a variety of tumors. In this context, we aimed at assessing the diagnostic and prognostic value of the recently described long intergenic non-coding RNA 01087 (LINC01087) in human cancers. (2) Methods: We studied the expression of LINC01087 across 30 oncological indications by interrogating public resources. Data extracted from the TCGA and GTEx databases were exploited to plot receiver operating characteristic curves (ROC) and determine the diagnostic performance of LINC01087. Survival data from TCGA and KM-Plotter directories allowed us to graph Kaplan–Meier curves and evaluate the prognostic value of LINC01087. To investigate the function of LINC01087, gene ontology (GO) annotation and Kyoto Encyclopedia of Gene and Genomes (KEGG) enrichment analyses were performed. Furthermore, interactions between LINC01087 and both miRNA and mRNA were studied by means of bioinformatics tools. (3) Results: LINC01087 was significantly deregulated in 7 out of 30 cancers, showing a predominant upregulation. Notably, it was overexpressed in breast (BC), esophageal (ESCA), and ovarian (OV) cancers, as well as lung squamous cell carcinoma (LUSC), stomach adenocarcinoma (STAD), and uterine carcinosarcoma (UCS). By contrast, LINC01087 displayed downregulation in testicular germ cell tumors (TGCT). ROC curve analyses identified LINC01087 as a potential diagnostic indicator in BC, ESCA, OV, STAD, and TGCT. Moreover, high and low expression of LINC01087 predicted a favorable prognosis in BC and papillary cell carcinoma, respectively. In silico analyses indicated that deregulation of LINC01087 in cancer was associated with a modulation of genes related to ion channel, transporter, and peptide receptor activity. (4) Conclusions: the quantification of an altered abundance of LINC01087 in tissue specimens might be clinically useful for the diagnosis and prognosis of some hormone-related tumors, including BC, OV, and TGCT, as well as other cancer types such as ESCA and STAD. Moreover, our study revealed the potential of LINC01087 (and perhaps other lncRNAs) to regulate neuroactive molecules in cancer.

## 1. Introduction

Carcinogenesis is a dynamic process that inexorably and time-dependently perturbs the normal functions of an organism [1,2]. Besides environmental and lifestyle factors, genetic and epigenetic risk factors contribute dramatically to the transformation of normal cells into malignant cells [1]. Consequently, cancers are highly heterogeneous and complex. In this context, patients can effectively benefit from precision medicine, which may lead to more efficient diagnostic and therapeutic strategies.

Progress in high-throughput technologies contributed to the identification of new molecules, referred to as biomarkers, such as gene sequences, proteins, and metabolites, which are quantifiable in a variety of human samples (e.g., tissue biopsies, blood, feces, sperm, and urine) [3,4,5,6]. This allows biomarkers to precisely discriminate between healthy and disease status, as well as to predict clinical outcomes. Epigenetic modifications, such as aberrant DNA methylation (hypermethylation of tumor suppressor genes or hypomethylation of oncogenes), histone modifications, and dysregulated non-coding RNAs, have been explored as potential biomarkers in the diagnosis and follow-up of patients [7,8,9,10,11]. For instance, the methylation of the O(6)- methylguanine- DNA methyltransferase (*MGMT*) gene is recognized as a potential prognostic/predictive marker in glioblastoma [12]. Moreover, high DNA methylation of the gene coding for branched chain amino acid transaminase 1 (*BCAT1)* in plasma may distinguish between lung disease and healthy individuals [13]. Recently, Meyer and colleagues identified DNA methylation patterns that correlate with the response to neoadjuvant chemotherapy in triple-negative breast cancer (TNBC) [14]. Non-coding RNAs, including long (lncRNAs), short (miRNAs), and circular (circRNAs) RNAs, have been recognized as a promising type of early diagnostic and prognostic epigenetic biomarkers in human diseases, taking advantage of their stability to ribonucleases in solid tissues and body fluids [11,15,16,17,18,19,20,21]. LncRNAs consist of transcripts longer than 200 nucleotides, lacking open reading frames [22,23,24]. They can act as tumor suppressors or oncogenes, thereby contributing to cancer pathogenesis and progression when deregulated in tumors [25,26]. Moreover, several studies reported an association between altered lncRNA expression (up or down) and patient survival [25,27]. For instance, a high abundance of the Hox transcript antisense intergenic RNA (HOTAIR) is associated with an unfavorable prognosis in various malignancies, including bladder, gastric, and esophageal squamous cell carcinomas [28,29,30]. Inversely, a decreased expression of the maternally expressed gene 3 (*MEG3*) lncRNA predicts poor survival in breast cancer (BC) patients [31]. Additionally, in the clinical management of highly heterogeneous malignancies, such as BC or lung cancers, lncRNAs appeared to be highly efficient in discriminating between the different subtypes of organ-specific malignancies [32,33]. Notably, Li and colleagues identified three different molecular subtypes in glioma patients using a lncRNA expression signature [34]. Furthermore, a lncRNA-based molecular classification was described in BC. In particular, among the 4 clusters described, clusters I and II correlated with the basal-like and HER2-enriched subtypes (according to the Prediction Analysis of Microarray 50, PAM50, classification), respectively [35].

The long intergenic non-coding RNA 01087 (LINC01087) is a recently described lncRNA whose function remains poorly investigated. The contribution of LINC01087 to the pathogenesis of cancer, as well as its pivotal role as a biomarker, has been described only in a few oncological indications (i.e., BC, glioma, esophageal squamous cell carcinoma, and thyroid cancer) [32,36,37,38,39]. Recent evidence reported LINC01087 to be overexpressed in glioma patients with respect to healthy subjects [36,37]. Mechanistically, it has been demonstrated that LINC01087 participates in the development of glioma by targeting some microRNAs (miRNAs) (i.e., miR-384 and miR-1277-5p) and, therefore, altering the related signaling pathways [36,37]. For instance, it has been demonstrated in vitro that a high expression of LINC01087 reduces the level of Bcl2 by acting as a miR-384 sponge, thus promoting apoptosis in glioma cells [36]. In addition, its overexpression promotes proliferation, migration, and invasion of thyroid cancer cells through the upregulation of the gene protein phosphatase, Mg2^+^/Mn2^+^ dependent 1E (*PPM1E*) [38]. Furthermore, LINC01087 demonstrated a strong interest in the stratification and prognosis of BC patients [32]. Indeed, the abundance of LINC01087 can discriminate between the luminal and triple-negative (TNBC) subtypes of BC. Furthermore, a high tumor level of this lncRNA is an indicator of prolonged overall survival in BC patients [32].

Here, we shed light on the value of LINC01087 as an indicator for the diagnosis and prognostication of multiple cancer types. Using bioinformatics methods, we first assessed the level of expression of LINC01087 across several tumors. Then, we validated the diagnostic value of LINC01087 using receiver operating characteristics (ROC) curves analysis. We correlated its altered expression with patient survival and clinicopathological features. Finally, the potential functions of LINC01087 were identified by building co-expression networks and gene ontology (GO) enrichment analyses. Our results revealed that LINC01087 is mainly upregulated in multiple cancers, particularly in BC, esophageal, stomach, and ovarian carcinomas, as well as in testicular germ cell tumors. Additionally, LINC01087 could serve as a prognostic indicator in BC and papillary renal cell carcinoma. Interestingly, in silico analyses revealed a potential role for LINC01087 in carcinogenesis through the regulation of gene products that sense or exchange extracellular molecules.

## 2. Materials and Methods

### 2.1. Transcriptomics Data Analysis

RNA-sequencing (RNA-seq) and clinical data of 24 primary tumors and paired normal tissues were extracted from The Cancer Genome Atlas (TCGA) pan-cancer 2018 online repository using TCGAbiolinks package in R [23,24,40]. RNA-seq data of 27 types of healthy organs/tissues were downloaded from Genotype-Tissue Expression (GTEx) portal (http://www.gtexportal.org/home/datasets, 1 August 2022). To perform the differential expression analysis of LINC01087, we compared the transcriptomic data of primary tumors extracted from TCGA to paired normal tissues (also retrieved from TCGA) combined with healthy samples of the tissue of origin (extracted from GTEx). The TCGA and GTEx datasets originally comprised 32 and 35 tissues, respectively. However, we excluded several breast cancer and liver subtypes (total of 8 tissues) from both datasets, as well as some organs/tissues with no data (n = 3) from GTEx, resulting in 24 (TCGA) and 27 (GTEx) tissues, reflecting a total of 30 distinct tissues. Of note, only datasets with information about LINC01087 expression were included. Samples with some unassigned values (i.e., “NA”) were removed. Analysis of the differential expression of LINC01087 between normal and malignant tissues was performed using DeSeq2 R/Bioconductor in the 30 types of organs/tissues. LINC01087 was considered as significantly deregulated in cancer when log_2_ fold change (FC) absolute value ≥ 1.5 and *p*-value ≤ 0.05 (Wilcoxon test). For each dataset, the differential expression of LINC01087 was calculated based on the dataset median value used as a threshold. The association between the expression of LINC01087 in different tumor types and the clinicopathological parameters (i.e., age, gender, tumor stage, tumor size, tumor status, lymph node, and metastasis involvement) was calculated using Fisher’s exact test. For each dataset, a high versus low discrimination of LINC01087 expression was achieved, using the dataset median value as a threshold.

### 2.2. Survival Curve Analysis

To evaluate the effect of LINC01087 on the overall survival (OS) across patients affected with various cancer histotypes, we used the pan-cancer RNA-seq data from TCGA (https://portal.gdc.cancer.gov 15 September 2022) and the Kaplan–Meier Plotter (KM-Plotter) datasets (https://kmplot.com, 2 July 2022) [41,42]. In KM-Plotter, survival curves were generated by selecting the three following filters: median, JetSet best probe set, and all datasets. Survival R package v.3.4 and Survminer R package v.0.4.9 were applied to TCGA datasets in order to calculate the hazard ratio (HR), 95% confidence intervals (CI), and *p*-values. For each TCGA dataset, depending on the number of available samples, patients were subdivided into two groups according to the median expression level of LINC01087: high or low. A *p*-value ≤ 0.05 was considered statistically significant.

### 2.3. ROC Curve Analysis

ROC curves were generated and the area under the curve (AUC) was calculated using the R packages MASS and pROC [43,44]. All tests were performed in R statistical software (ver. 4). AUC ≥ 0.7 and *p*-value ≤ 0.05 were applied as a cut-off criterion to consider LINC01087 as a good diagnostic biomarker. For each dataset, a high versus low discrimination of LINC01087 expression was considered based on the dataset best cut-off value.

### 2.4. Network and Pathway Enrichment Analyses of LINC01087

For the identification of interactions between LINC01087 and mRNAs and miRNAs, we employed a dual strategy. In the first step, we performed a Spearman correlation test to identify mRNAs and miRNAs that correlated with LINC01087 expression in BC, ESCA, OV, STAD, and TGCT by extracting data from TCGA datasets. We defined significant associations as having a correlation coefficient (R) > 0.4 and a *p*-value < 0.05. Volcano plots and Venn diagrams were drawn using EnhancedVolcano and VennDiagram R packages, respectively. In the second step, we used the web tool DIANA-LncBASE v3 (https://diana.e-ce.uth.gr/lncbasev3, 14 October 2022) to identify candidate miRNA targets of LINC01087 [45]. Then, we took advantage of the miRTarBase (https://mirtarbase.cuhk.edu.cn/~miRTarBase/miRTarBase_2022/php/index.php, 20 October 2022) to predict mRNA targets of the miRNAs derived from both analyses [46]. Finally, the target mRNA candidates were compared with the mRNAs that correlated with LINC01087 expression in the initial analysis.

To investigate the biological pathways and processes, Gene Ontology (GO) and Kyoto Encyclopedia of Genes and Genomes (KEGG) term enrichment analyses were performed using the clusterProfiler R package on genes significantly associated with LINC01087 expression (R > 0.1 *p* < 0.05) in BC, ESCA, STAD, and TGCT tumor tissues derived from TCGA (*p*-value were adjusted using the Benjamini–Hochberg procedure).

## 3. Results

### 3.1. Diagnostic Value of LINC01087

To assess the diagnostic potential of LINC01087, we measured its abundance in 30 types of tissue/organs extracted from the TCGA and GTEx databases (Table 1). In particular, we compared the transcriptomics data of primary tumors from TCGA to paired normal tissues (retrieved from TCGA) combined with healthy samples of the tissue of origin (extracted from GTEx).

**Figure 1:**
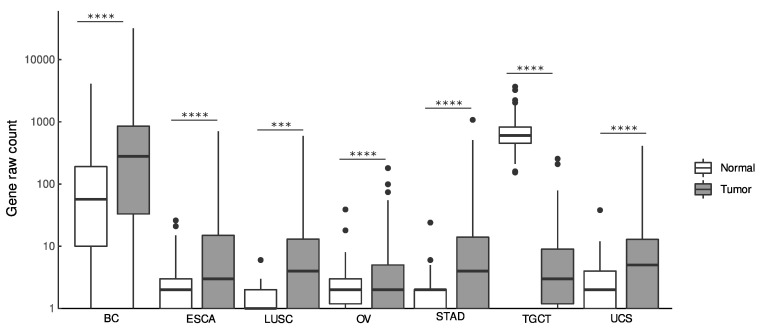
Deregulation of LINC01087 expression in representative cancers. Boxplots depicting the expression level of LINC01087 in normal and malignant tissues derived from the TCGA and GTEx databases. Data show median, quartiles, and individual values that are expressed as gene raw count. Comparison was performed using Wilcoxon rank-sum test, *** *p* ≤ 0.001, **** *p* ≤ 0.0001. Abbreviations: BC, breast cancer (n = 1685); ESCA, esophageal carcinoma (n = 1619); GTEx, genotype-tissue expression; LUSC, lung squamous cell carcinoma (n = 551); OV, ovarian cancer (n = 561); STAD, stomach adenocarcinoma (n = 766); TCGA, the cancer genome atlas; TGCT, testicular germ cell tumors (n = 517); UCS, uterine carcinosarcoma (n = 199).

LINC01087 demonstrated significant upregulation (|log2 FC| ≥ 1.5, *p* ≤ 0.05) in 6 out of the 30 cancers as compared to their normal (i.e., non-tumor or healthy) tissue counterparts (Figure 1 and Table 1).

Notably, LINC01087 displayed elevated levels in BC (log2FC = 2.2, *p* = 2.32e-35), esophageal carcinoma (ESCA, log2FC = 3.7, *p* = 4.08e-35), lung squamous cell carcinoma (LUSC, log2FC = 2.73, *p* = 0.003), ovarian cancer (OV, log2FC = 1.5, *p* = 1.11e-14), stomach adenocarcinoma (STAD, log2FC = 4, *p* = 2.49e-50), and in uterine carcinosarcoma (UCS, log2FC = 3.2, *p* = 0.0001) (Figure 1 and Table 1). In addition, a smaller (log2FC < 1.5) but significantly (*p* ≤ 0.05) altered expression of LINC01087 was detected in clear cell (ccRCC, log2FC = 0.8, *p* = 1.25e-15) and papillary (pRCC, log2FC = 0.9, *p* = 0.005) renal cell carcinomas, hepatocellular carcinoma (HCC, log2FC = 0.8, *p* = 4.28e-07), lung adenocarcinoma (LUAD, log2FC = 1.3, *p* = 0.002), rectum adenocarcinoma (READ, log2FC = 0.7, *p* = 0.001), and skin cutaneous melanoma (SKCM, log2FC = 0.5, *p* = 7.99e-23) (Table 1).

By contrast, LINC01087 demonstrated a strong downregulation in testicular germ cell tumors (TGCT, log2FC = −6.1, *p* = 6.59e-73) (Figure 1 and Table 1). Moreover, it was less (log2FC ≥ −1.5), but significantly (*p* ≤ 0.05) expressed in acute myeloid leukemia (LAML, log2FC = −1, *p* = 9.46e-08), brain lower grade glioma (LGG, log2FC = −0.3, *p* = 2.43e-04), pancreatic adenocarcinoma (PAAD, log2FC = −1.2, *p* = 6.86e-20), pheochromocytoma and paraganglioma (PGPG, log2FC = −0.7, *p* = 3.87e-07), and thyroid carcinoma (THCA, log2FC = −0.6, *p* = 3.13e-10) (Table 1).

Next, we measured the diagnostic performance of LINC01087 by plotting and interpreting ROC curves (Table 1). Values of area under the curve (AUC) superior to 0.7 designated LINC01087 as a good diagnostic biomarker in BC (AUC = 0.7, *p* = 1.16e-35), as well as esophageal carcinoma (AUC = 0.8, *p* = 2.04e-35), ovarian (AUC = 0.7, *p* = 5.56e-15) and stomach (AUC = 0.8, *p* = 1.25e-50) cancers, and in testicular germ cell tumors (AUC = 1, *p* = 3.30e-73) (Table 1). Furthermore, we observed lower (AUC < 0.7), but significant (*p*< 0.05), AUC values of LINC01087 in clear cell (ccRCC: AUC = 0.7, *p* = 6.24e-16) and papillary renal cell carcinomas (pRCC: AUC = 0.6, *p* = 0.002), acute myeloid leukemia (AML: AUC = 0.6, *p* = 4.73e-08), lower grade glioma (LGG: AUC = 0.5, *p* = 0.0001), hepatocellular carcinoma (HCC: AUC = 0.6, *p* = 2.14e-07), lung adenocarcinoma (LUAD: AUC = 0.5, *p* = 0.001), lung squamous cell carcinoma (LUSC: AUC = 0.6, *p* = 0.001), pheochromocytoma and paraganglioma (PCPG: AUC = 0.6, *p* = 1.93e-07), skin cutaneous melanoma (SKCM: AUC = 0.6, *p* = 3.99e-23), and uterine carcinosarcoma (UCS: AUC = 0.6, *p* = 6.69e-05) (Table 1).

Finally, we investigated whether the deregulation of LINC01087 could be associated with clinicopathological features (i.e., age, gender, tumor stage, tumor size, tumor status, lymph node, and metastasis involvement). To address this point, we focused on cancers in which LINC01087 showed both up- or down-regulation (|log2FC|> 1.5, *p* < 0.05) and diagnostic performance (AUC > 0.7, *p* < 0.05), when such information was available. These criteria restricted the analysis to BC, ESCA, OV, STAD, and TGCT (Table 2, Table S1). LINC01087 expression appeared to be significantly associated with the tumor status (*p* = 0.04) and tumor size (*p* = 0.01) in BC (Table 2). In TGCT, LINC01087 significantly correlated with the size of tumor (*p* = 0.05) (Table 2).

Altogether, these results imply that the perturbation of LINC01087 expression might play a critical role in carcinogenesis. Moreover, LINC01087 shows great potential as an indicator for the diagnosis of BC, as well as esophageal carcinoma and stomach adenocarcinomas, OV and TGCT. These findings require further experimentation to validate such interest.

### 3.2. Prognostic Value of LINC01087 in Cancer

Subsequently, we examined the prognostic value of LINC01087 by evaluating the relationship between the expression of LINC01087 and patient overall survival (OS). For this purpose, cohorts gathered in the TCGA directory were used for derivation, while inputs of the KM-Plotter database initially served as validation cohorts.

Low expression of LINC01087 denoted a favorable prognosis in pRCC (n = 412, *p* = 2.86E-04) (Table 1). Contrarily, the presence of LINC01087 correlated with a better OS in patients affected with BC (n = 1685, *p* = 0.01). Such prognostic interest was validated using KM-Plotter datasets (Table 1).

Further interrogation of the KM-Plotter database revealed that a low abundance of LINC01087 matched an extended OS in HCC, as well as in uterine corpus endometrial carcinoma (Table 1).

### 3.3. Impact of LINC01087 on Cancer-Related Pathways

**Figure 2:**
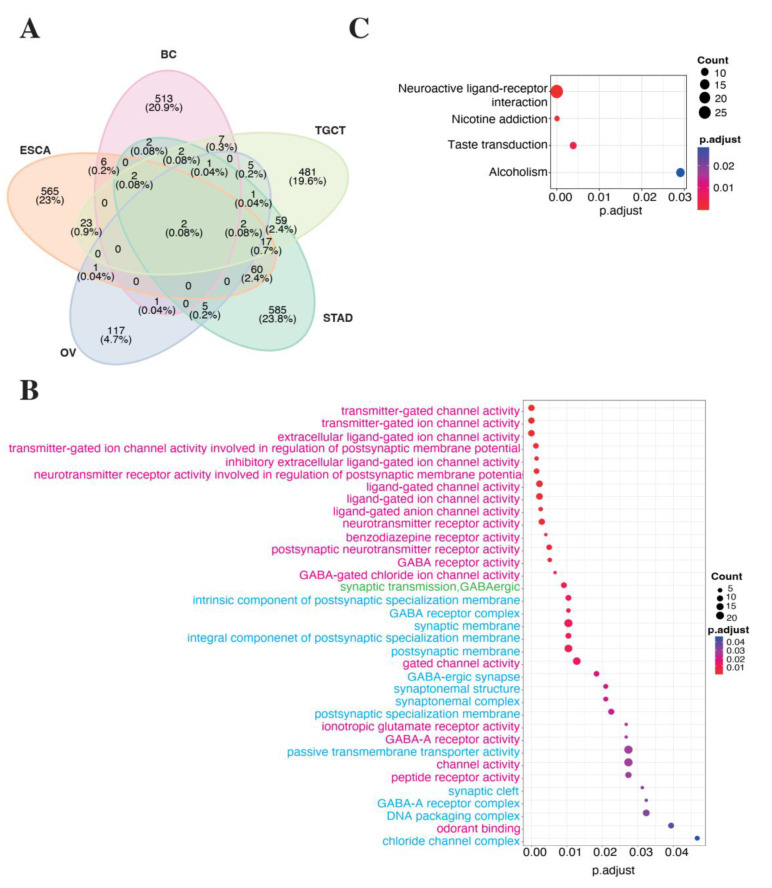
LINC010187-related gene expression changes in representative cancers. (**A**) Venn diagram showing the overlapping genes positively associated with LINC01087 in cancers where it shows a strong diagnostic interest. The percentage of overlapping genes is reported in brackets. (**B**,**C**) Bubble maps depicting results of (**B**) GO term and (**C**) KEGG pathway enrichment analyses. The size of the circles indicates the number of genes in each set (count), while the color code informs about the adjusted *p*-values (*p*.adjust). Panel (**B**) illustrates the annotated clusters that included ≥ 4 genes. Panel (**B**) also indicates the “Biological process”, “Cellular component”, and “Molecular function” subcategories of GO hierarchy in turquoise, green, and fuchsia, respectively. See Supplementary Tables S7 and S8 for details. Abbreviations: BC, breast cancer; ESCA, esophageal carcinoma; GO, gene ontology; KEGG, Kyoto encyclopedia of genes and genomes; OV, ovarian cancer; STAD, stomach adenocarcinoma; TGCT, testicular germ cell tumors.

To capture significant insights into LINC01087 functions in cancers, we performed a correlation-based expression analysis extracting data from TCGA datasets. In particular, we looked for genes that significantly correlated (Spearman correlation test: R ≥ |0.4|, *p* ≤ 0.05) with LINC01087 expression in each of the tissue types where LINC01087 demonstrated a strong diagnostic potential (log2 FC ≥ |1.5|, *p* ≤ 0.05, and AUC ≥ 0.7, *p* ≤ 0.05), namely BC, ESCA, OV, STAD, and TGCT (Analysis 1—Table 1, Figure S1, and Tables S2–S6). We found significant positive (R ≥ 0.4, *p* ≤ 0.05) correlations between the expression of LINC01087 and 574 transcripts in BC, 691 in ESCA, 141 in OV, 773 in STAD, and 636 in TGCT (Tables S2–S6). LINC01087 was associated with pseudogenes, microRNAs (miRNAs), and other lncRNAs, as well as with protein-coding genes. Out of the 10 transcripts that were the most significantly correlated with LINC01087 in BC, 6 were pseudogenes, 2 were lncRNAs, and 2 were protein-coding genes belonging to the POTE family (*POTEH, POTEI*). Similarly, in ESCA, among the top 10 genes associated with LINC01087 were pseudogenes and lncRNAs, as well as the neurofilament heavy polypeptide (*NEFH*) gene, whose deregulation had been associated with the development of several malignancies, such as BC [47]. Several genes were negatively and modestly (R ≤ −0.2, *p* ≤ 0.05) correlated with LINC01087; therefore, they were not considered for further analysis. To refine our analysis, we compared the correlating gene sets of every cancer type (R ≥ 0.4, *p* ≤ 0.05) for overlapping genes. We identified 2 transcripts that were positively associated with LINC01087 expression in all 5 cancers: the pseudogene POTE ankyrin domain family member K (*POTEKP*) and the uncharacterized lncRNA AC093838.1 (Figure 2A).

*POTEKP* showed the strongest positive correlation with LINC01087 in BC (R = 0.96), ESCA (R = 0.95), STAD (R = 0.93), and TGCT (R = 0.98), with the exception of OV, where it showed a moderate yet significant correlation (R = 0.61) (Tables S2–S6). In OV, *AC093838.1* demonstrated the strongest association with LINC01087 (R = 0.91). The latter transcript also displayed a strong correlation with LINC01087 in the 4 other types of cancer (Tables S2–S6). In addition, the transcript of mediator complex subunit 15 pseudogene 4 (*MED15P4*) showed a strong association with LINC01087 in 3 out of the 5 tumor types (i.e., OV, STAD and TGCT), and a moderate correlation in ESCA (Tables S2–S6).

In the next step, we aimed at understanding the biological impact of LINC01087 in cancer. To this aim, we performed GO annotation and KEGG enrichment analyses on the set of genes correlated with LINC01087 expression in the 5 tumor types (n = 2458, |R| ≥ 0.1, *p* ≤ 0.05). We identified a total of 35 GO terms subcategorized as follows: 1 “biological process” (BP), 13 “cellular component” (CC) and 21 “molecular function” (MF) (Figure 2B, Table S7). Among them, the 3 largest GO terms referred to “channel activity”, “passive transmembrane transporter activity”, and “synaptic membrane”. Notably, these clusters encompassed genes encoding gamma-aminobutyric acid [GABA] receptors (i.e., *GABRG1*, *GABRG2*, *GABBR2*, and *GABRA2*,) and glutamate ionotropic receptors (i.e., *GRIN3A*, *GRIA2*, *GRIA4*, and *GRIN1*) (Figure 2B, Table S7). Of note, this annotation analysis revealed an over-representation of genes uncovered in ESCA, such as *GABRA1*, *GRIN3A GRIA2*, *GRIA4*, and *GLRB* (Figure 2B, Supplementary Table S7). *GABRG2* was linked with both ESCA and STAD. *AQP11*, *KCNH3*, *GABRA6*, and *CHRNA10* were exclusively associated with TGCT, while *GJA8* and *CLCN1* were correlating with LINC01087 only in OV (Figure 2B, Table S7). Concordant results were obtained using the KEGG annotation analysis (Figure 2C, Table S8).

In summary, this orthogonal investigation revealed that LINC01087 might impact cancer progression by modulating the ability of cells to interact with their environment.

### 3.4. LINC01087 Functions as Competing Endogenous RNA

LncRNAs participate in carcinogenesis by modulating gene expression via either direct binding to mRNAs, or indirectly by functioning as a competing endogenous RNA (ceRNA) in the cytoplasm. This latter mechanism is characterized by the binding of lncRNAs to miRNAs, thus preventing their interaction with target mRNAs [48]. LINC01087 displays a cytoplasmic distribution as predicted by lncLocator database (score = 0.76, Table S9). Therefore, we investigated the potential of LINC01087 to act as a miRNA sponge.

**Figure 3:**
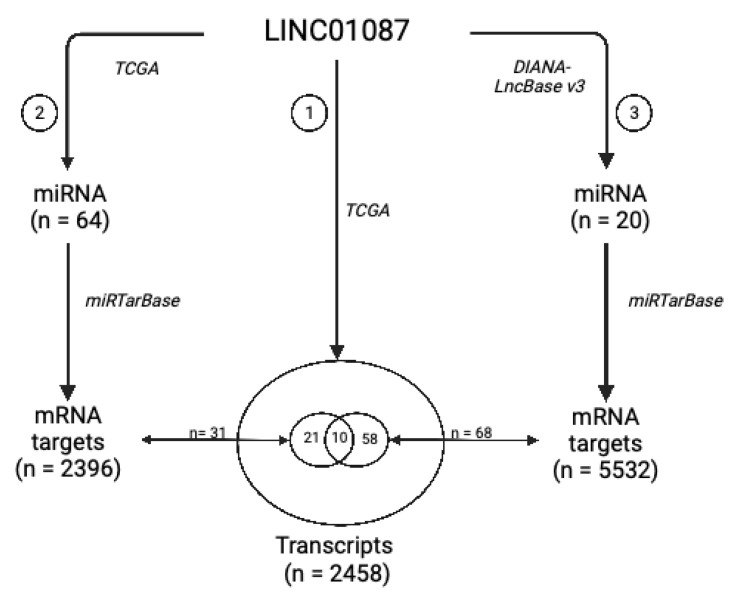
Schematic representation of the strategy used to characterize the LINC01087-miRNA-mRNA network. A Spearman correlation test was applied to TCGA datasets to identify 2458 transcripts (Analysis 1) and 64 miRNAs (Analysis 2), the expression of which correlated with that of LINC01087 in BC, ESCA, OV, STAD, and TGCT. In parallel, the DIANA-LncBASE v3 toolbox allowed to discover 20 miRNAs that interacted with LINC01087 (Analysis 3). Next, the miRTarBase predicted 2396 mRNA targets of the miRNAs identified in Analysis 2, and 5532 mRNA targets of the miRNAs identified in Analysis 3. The expression of 31 among these mRNA targets from Analysis 2 and 68 mRNA targets from Analysis 3 had been correlated with that of LINC01087 in Analysis 1. Among them, 10 messenger RNAs were shared between the three analyses, hence representing the core of the genes regulated by LINC0187 and related miRNAs. The resulting LINC01087-miRNA-mRNA network is detailed in Table 3.

To construct a potential LINC01087-miRNA-mRNA network, we used a dual strategy. In a first step based on the TCGA datasets, we screened for miRNAs the expression of which correlated with that of LINC01087 in the 5 types of cancer (R ≥ |0.4|, *p* ≤ 0.05) (Analysis 2—Figure 3). Sixty-four miRNAs were positively correlated with the expression of LINC01087 (Figure 3, Table S10). The top 5 transcripts demonstrated a moderate correlation (0.64 ≤ R ≤ 0.72) and comprised miR-5692B, miR-4666A, miR-31199-1, miR-1252, and miR-7157 (Table S10). Then, the miRTarBase was interrogated, and 2396 potential mRNA targets of these 64 miRNAs were identified (Figure 3). Finally, we compared this list of candidates (n = 2396, Analysis 2) with the initial clusters of genes positively correlated with LINC01087 in the 5 tumor types from TCGA (n = 2458, Analysis 1). A total of 31 genes overlapped between the two datasets (Figure 3, Table S11).

In the second step, we used DIANA-LncBASE v3 to discover miRNAs interacting with LINC01087. A total of 20 miRNAs were identified as potential targets of LINC01087 (Analysis 3—Figure 3, Table S12). Most of them had already been involved in carcinogenesis, such as miR-19a-3p and miR-181-5p [49,50]. Furthermore, 4 of the 20 miRNAs belonged to the let-7 miRNA family (i.e., hsa-let-7a-5p, hsa-let-7d-5p, hsa-let-7e-5p, hsa-miR-98-5p). However, no overlapping miRNAs were observed between the DIANA- and TCGA-based analyses. Then, we executed the miRTarBase to detect mRNA targets of these 20 miRNAs. A total of 5532 mRNA targets were identified in Analysis 3. Sixty-eight of them were common with the list of 2458 genes identified in Analysis 1 (Figure 3, Table S13).

Next, we compared the 31 mRNA targets annotated in Analysis 2 with the 68 mRNA targets uncovered in Analysis 3 and identified 10 overlapping messengers representing putative targets of LINC01087: chromosome 8 open reading frame 37 (*C8orf37*), ephrin A5 (*EFNA5*), hook microtubule-tethering protein 3 (*HOOK3*), myozenin 3 (*MYOZ3*), pleomorphic adenoma gene 1 (*PLAG1*), DNA Polymerase Iota (*POLI*), solute carrier family 2member 3 (*SLC2A3*), small nuclear ribonucleoprotein polypeptide A’ (*SNRPA1*), as well as two isoleucine/glutamine (IQ) motif genes, namely Purkinje cell protein 4 like 1 (*PCP4L1*) and IQ motif containing G (*IQCG*) (Figure 3, Tables S11 and S13).

Ultimately, this study led to the construction of a core network of 10 mRNA targets and 28 miRNAs related to LINC01087 (Table 3).

In more detail, LINC01087 seemed to interfere with 2 miRNAs targeting the messenger of *C8orf37* (namely has-miR-644a and has-miR-7-5p), *EFNA5* (hsa-miR-1305, hsa-miR-92a-3p), *HOOK3* (hsa-miR-4505, hsa-miR-181a-5p), *MYOZ3* (hsa-miR-7975, hsa-miR-197-3p), and *PCP4L1* (hsa-miR-4505, hsa-miR-423-5p), with 3 miRNAs targeting the mRNAs of *SLC2A3* (hsa-miR-606, hsa-miR-34a-5p, hsa-miR-98-5p) and *SNRPA1* (hsa-miR-3174, hsa-miR-34a-5p, hsa-miR-98-5p), and 4 miRNAs interfering with the transcripts of *IQCG* (hsa-miR-3671, hsa-miR-181a-5p, hsa-miR-181b-5p, hsa-miR-181d-5p), *PLAG1* (hsa-miR-569, hsa-miR-181a-5p, hsa-miR-181b-5p, hsa-miR-98-5p), and *POLI* (hsa-miR-4255, hsa-miR-19a-3p, hsa-miR-19b-3p, hsa-miR-92a-3p). Interestingly, most of these mRNAs and miRNAs have been connected to tumor suppressive and/or oncogenic functions in multiple cancers [49,50,51,52,53,54,55,56,57,58,59,60,61,62,63,64,65,66,67].

Altogether, these results documented a constellation of both protein-coding and translation-interfering transcripts that are directly or indirectly regulated by LINC01087 and that might contribute to the initiation and progression of neoplasms.

## 4. Discussion

LncRNAs represent promising epigenetic diagnostic, prognostic, and therapeutic biomarkers [25]. No less than 23 trials (source: clinicaltrials.gov (accessed on 1 August 2022)) aim at evaluating their clinical relevance in a variety of malignancies, including bladder (NCT05270174), colorectal (NCT04729855), gastric (NCT05397548, NCT05334849), head and neck (NCT04946968), kidney (NCT04946266), liver (NCT05088811), and prostate (NCT05141383) cancers. In the present report, bioinformatics analyses uncovered the role of LINC01087 in the diagnosis and prognostication of different human cancers.

To examine the clinical interest of LINC01087, we first analyzed its expression across a large array of tumor types using TCGA and GTEx databases, then calculated the ROC scores to assess its diagnostic performance. Among the 30 cancers evaluated, LINC01087 displayed a significant modulation of its expression in 7 of them, mainly hormone-dependent. More precisely, LINC01087 showed an increased expression in BC, OC, and UCS, and an inversely reduced level in TGCT as compared to non-tumor/healthy control samples. Additionally, an elevation of LINC01087 expression was witnessed in LUSC, as well as in two digestive cancers, namely ESCA and STAD.

Our results were consistent with the literature regarding the abundance of LINC01087 in BC and ESCA [36,37,38,68]. Nevertheless, previous research reported a higher expression of LINC01087 in THCA patients than in normal individuals [38], which was not validated in this study. This discrepancy could originate from differences in the size of the cohorts (n = 1224 in the present work versus n = 30 in the former article by Yin et al.) as well as the detection method applied (RNA-seq versus RT-qPCR) [38].

Out of the 7 cancers harboring an aberrant expression of LINC01087, a diagnostic test revealed promising performances in 5 of them (i.e., ROC curve AUC > 0.7). In detail, the measurement of LINC01087 levels was an excellent (AUC = 1) indicator of malignancy in TGCT, and a fair (0.7 < AUC ≤ 0.8) diagnostic indicator in BC, ESCA, OV, and STAD. Of note, we reported that the measurement of the level of LINC01087 in BC also allowed us to further distinguish between the luminal and triple-negative subtypes [32].

Next, we evaluated the prognostic value of LINC01087 by comparing cancer patient OS according to its level of expression using the cohorts of TCGA and KM-plotter databases for derivation and validation, respectively. LINC01087 deregulation predicted patient outcome in two oncological indications. First, a downregulation of LINC01087 correlated with favorable survival in patients suffering from pRCC. Second, a high expression level of LINC01087 indicated a better prognosis in subjects affected with BC.

We proceeded by evaluating the potential functional significance of LINC01087 in the 5 cancer types in which it was most upregulated, namely, BC, ESCA, OV, STAD, and TGCT. At first sight, we screened for genes correlated with LINC01087 expression in the 5 tumor types. Such analysis identified 2 transcripts overlapping in the 5 cancer types, namely POTEKP and AC093838.1. These genes are both located on chromosome 2 but their role in human malignancies has not been elucidated.

Interestingly, GO annotation and KEGG enrichment analyses performed on the genes positively correlated with LINC01087 expression in BC, ESCA, OV, STAD, and TGCT, found a spectrum of genes and pathways associated with the regulation of ion channel activities and neurotransmission. This cluster consisted mainly of genes related to the receptors for GABA (i.e., *GABRG1*, *GABRG2*, *GABBR2*, *GABRA2*, *GABRA6*) and α-amino-3-hydroxy-5-methyl-4-isoxazolepropionic acid (AMPA) (i.e., *GRIN3A*, *GRIA2*, *GRIA4*, and *GRIN1*). Based on this information, we speculate that the involvement of LINC010187 in tumorigenesis might be associated with the “neuro-cancer axis” [69]. Of note, neuronal cells and immune and cancer cells can synthesize and release neurotransmitters such as GABA [70,71,72,73]. GABA exerts its multiple functions through ionotropic GABA A receptors and metabolotropic GABA B receptors. The aberrant expression of GABA and GABA receptors has been associated with cancer development, metastasis, and suppression of immunosurveillance [71,74]. In this scenario, lncRNAs, as well as miRNAs, may regulate neuronal activity and target GABA receptors [59,75,76]. For instance, one recent study examined the role of the lncRNA Gm37494 in the context of osteoarthritis, focusing on its capability to target *GABRA1* by binding to miR-181a-5p [76]. Therefore, it appears plausible, yet remains to be demonstrated, that LINC01087 (and probably other lncRNAs) regulate GABA receptor activity during the formation and progression of specific cancer types.

LncRNAs can regulate gene expression by interacting with DNA, RNA, and proteins [23]. Notably, they can either directly alter the transcription of neighboring and distant protein-coding genes, or indirectly, by targeting and thereby modulating the expression of miRNAs acting as ceRNAs. Therefore, we constructed a LINC01087-miRNA-mRNA network by interrogating the TCGA and DIANA-LncBASE datasets. Next, miRTarBase was used to discover possible interactions between miRNAs and mRNA targets in both datasets. Finally, we overlapped the mRNA targets with the cluster of genes correlated with LINC01087 expression. Our analysis yielded a list of 28 miRNAs and 10 mRNAs that constituted a core panel of putative targets of LINC01087.

Some mRNAs and miRNAs of the established LINC01087 network are related to malignant disease [51,52,53]. For instance, miR-3174 (targeting *SNRPA1* mRNA) exhibits oncogenic activity, whereas miR-34a-5p and hsa-miR-644a (targeting *SLC2A3* and *C8orf37* mRNAs, respectively) possess tumor-suppressive properties [60,61,77,78,79]. Nevertheless, most of the miRNAs related to LINC01087 (i.e., miR-7-5p, miR-19a-3p, miR-19-b-3p, miR-92b-3p, hsa-miR-98-5p, miR-181a-5p, miR-181d-5p, miR-197-3p, miR-423-5p, miR-569, and hsa-miR-1305) have an ambiguous impact on carcinogenesis, acting as either tumor suppressor genes or oncogenes depending on the tumor type [58,63,65,66,67,80,81,82,83,84,85,86,87,88]. Among them, miR-92b-3p inhibited the proliferation and invasion of pancreatic cancer cells through the downregulation of *GABRA3* [59]. In an analogous way, we found that LINC01087 expression was correlated with several GABRA genes, such as *GABRA2* and *GABRA6*. Furthermore, some miRNAs of the LINC01087 network like miR-34a-5p, miR-197-3p, miR-423-5p, and hsa-miR-3174, were reported as targets of lncRNAs. For instance, miR-34a-5p can be trapped by LINC00665, HOTAIR, or else nuclear paraspeckle assembly transcript 1 (*NEAT1*) [62,64,89,90,91,92,93]. *NEAT1* is also able to target miR-98-5p to promote glioma cancer progression [94]. Out of the 28 miRNAs, a few have been scarcely explored in cancer (e.g., miR-606, miR-3671, miR-4255, and miR-7975) [95,96,97]. For instance, miR-606 was mentioned in a single study to be upregulated in pancreatic cancer [98]. Similarly, one study reported a high expression level of miR-4255 in exosomes collected from patients with prostate cancer [99]. Moreover, Lin and collaborators demonstrated that the glycoprotein lipocalin 2 prevents oral cancer metastasis through miR-4505-mediated suppression of carbonic anhydrase IX expression [100].

Concerning the protein-encoding genes identified in the LINC01087 network, several of them were described as potential cancer-relevant biomarkers. Indeed, *PLAG1* was reported as an immunohistochemical marker in pleomorphic adenoma [52]. Moreover, *PLAG1* was found to be upregulated in OV, in which its silencing promoted chemosensitivity [50]. Another example concerns the role of *HOOK3* as a predictor of poor prognosis in prostate cancer [51]. Furthermore, in accordance with our data, He and colleagues predicted *HOOK3* as a target of miR-181-5p [101]. Similarly, upregulation of *POLI* predicted poor prognosis in esophageal squamous cell carcinoma (ESCC) patients [68,69,70]. Furthermore, the single amino acid polymorphism Thr706Ala in POLI was indicative of a higher risk of developing lung adenocarcinoma and squamous cell carcinoma in individuals of <61 years [102]. The aforementioned investigations support a possible role of the LINC01087-POLI interaction in cancer, in particular ESCA.

Here, we found a high expression of *SLC2A3* in ESCA. *SLC2A3*, also known as glucose transporter 3 (*GLUT3*), is a member of the SLC2 family, mediating glucose transport across the plasma membrane. The oncogenic potential of *SLC2A3* has been extensively investigated. An overexpression or copy number gain of *SLC2A3* has been witnessed in liquid and solid tumors [103,104,105,106,107,108]. For instance, high levels of *SLC2A3* were associated with unfavorable outcomes in CRC, gastric (GC), and head and neck squamous cell carcinoma (HNSCC) [105,106,107]. Additionally, *SLC2A3* seems directly targeted by miRNAs along tumorigenesis [109,110,111]. For instance, miR-29c inhibited cell growth and glucose metabolism in prostate cancer by targeting *SLC2A3* [110]. Moreover, a decreased expression of miR-106a increased the expression of *SLC2A3*, thereby contributing to progression and poor prognosis in glioblastoma patients [109]. In GC, cell glucose metabolism and growth are regulated by the miRNA-129-5p/SCL2A3 axis via the phosphatidylinositol 3-kinase/protein kinase B (PI3K-Akt) and mitogen-activated protein kinase (MAPK) signaling pathways [111]. Accordingly, we observed a relationship between LINC01087 and certain SLC membrane transporters (e.g., *SLC28A1*, *SLC9A3*, *SLC4A9*, and *SLC17A8*) in luminal and TNBC BC subtypes [32]. These similarities support an intimate link between the solute carrier family and LINC01087 in cancer.

*EFNA5*, a member of the ephrinA ligand family, is deregulated (up or down) in a variety of cancers [112,113,114,115,116]. A recent study explored the mRNA and protein expression, as well as DNA methylation, immune-related signature, and prognostic value of the EFNA family using a multi-omics integrative approach [56]. *EFNA5* mRNA was found to be overexpressed in most tumors compared to normal tissues. Moreover, an association between the mRNA level of *EFNA5* and DNA methylation of its promoter were documented for ccRCC, chRCC, pRCC, mesothelioma, prostate cancer, thymoma, and diffuse large B-cell lymphoma. At the protein level, EFNA5 was downregulated in glioblastoma multiforme and ccRCC, but highly expressed in endometrial carcinoma. In addition, a high abundance of *EFNA5* in both TCGA and gene expression omnibus (GEO) databases correlated with poor OS in patients with advanced LUAD [56].

The gene *SNRPA1*, a spliceosome component responsible for processing pre-mRNA into mRNA, showed strong prognostic potential in prostate cancer, HCC, and ccRCC [55,117,118]. Its oncogenic role in CRC has also been demonstrated [119]. Here, we observed high expression of *SNRPA1* in STAD, as well as a positive correlation between LINC01087 and *SNRPA1*. Interestingly, Chen and colleagues reported the interaction between the lncRNA RMRP (RNA component of mitochondrial RNA-processing endoribonuclease) and the protein SNRPA1 as a mediator of TP53 inactivation and as a promoter of cell proliferation in CRC [120].

*IQCG* is a protein-encoding gene involved in sperm axoneme assembly. Here, we reported an association between LINC01087, miR-181a-5p, and *IQCG* mRNAs in cancer. The link between miR-181a-5p and *IQCG* has previously been observed in endometrial carcinoma using an in silico approach [101].

Conversely, there is limited knowledge on *C8orf37* and *PCP4L1* in malignancies. While *C8orf37* has been only associated with genetic disorders, the possible involvement of *PCP4L1* in CRC and prostate cancer progression requires further confirmation [121,122,123,124].

Based on our results and previous observations, we speculate that the deregulation of LINC01087 might be related to the carcinogenesis of BC, ESCA, OV, STAD, and TGCT. However, the mechanisms behind LINC01087 involvement in the development/progression of these malignant diseases remain to be explored experimentally.

Since its discovery, interest in and functional knowledge of LINC01087 in the field of oncology have rapidly expanded. Further bench-to-bedside investigations are required to demonstrate the clinical relevance of LINC01087 as a diagnostic and prognostic biomarker.

## 5. Conclusions

In summary, the deregulation of LINC01087 plays a potential role in the diagnosis and prognosis of different tumor types. Notably, a high expression of LINC01087 exhibits a strong diagnostic value in breast, esophageal, stomach, and ovarian carcinomas. Moreover, a decreased expression of LINC01087 could be used for the diagnosis of malignant testicular germ tumors. LINC01087 also appears to predict the survival of patients suffering from breast and papillary kidney malignancies. Furthermore, the involvement of LINC01087 in cancer development might be related to its direct and indirect (miRNA-mediated) role in modulating biochemical exchanges between the intracellular milieu and the surrounding microenvironment. Future studies must shed light on the biological activity of LINC01087 and confirm its clinical utility as a diagnostic and/or prognostic biomarker. Ultimately, the quantitation of LINC01087 may ease clinical decision-making and improve the management of these oncological pathologies.

## Figures and Tables

**Table 1:** Diagnostic and prognostic value of LINC01087 in a variety of human cancers.

		Diagnosis					Prognosis			
		TCGA and GTEx					TCGA			KM Plotter
			Expression		Clinical Interest (ROC)		OS			
Type of Tumor	Abbreviation	N. of Samples (N, T)	*p*-Value	log2FC	*p*-Value	AUC	*p*-Value	HR (95%CI)	*p*-Value	HR (95%CI)
Adrenocortical carcinoma	ACC	337 (258, 79)	NS	−0.17	0.03	0.6	NS	1.15 (0.53–2.5)	NA	NA
Bladder cancer	BLCA	452 (40, 412)	NS	2.5	0.03	0.6	NS	1.01 (0.75–1.36)	NS	1 (0.74–1.37)
Breast cancer	BC	1685 (572, 1113)	**2.32E-35**	**2.2**	**1.16E-35**	**0.7**	**0.01**	**0.65** (**0.47–0.9**)	**0.0063**	**0.64** (**0.46–0.88**)
Cervical squamous cell carcinoma and endocervical adenocarcinoma	CESC	328 (22, 306)	NS	0.2	NS	0.5	NS	0.91 (0.57–1.44)	NS	0.92 (0.56–1.51)
Cholangiocarcinoma	CHOL	270 (235, 35)	NS	2.5	NS	0.5	NS	1.24 (0.46–3.38)	NA	NA
Colon adenocarcinoma	COAD	1300 (820, 480)	NS	0.8	0.05	0.5	NS	0.97 (0.61–1.52)	NA	NA
Esophageal carcinoma	ESCA	1619 (1456, 163)	**4.08E-35**	**3.7**	**2.04E-35**	**0.8**	NS	1.50 (0.92–2.45)	NS	1.7 (0.89–3.23)
Glioblastoma multiforme	GBM	2816 (2647, 169)	NS	−0.1	NS	0.5	NS	1.09 (0.76–1.57)	NA	NA
Lower grade glioma	LGG	3174 (2642, 532)	2.43E-04	−0.3	0.0001	0.5	NS	0.98 (0.63–1.52)	NA	NA
Head-neck squamous cell carcinoma	HNSC	710 (206, 504)	1.55E-11	0.1	7.77E-12	0.6	NS	0.92 (0.69–1.21)	NS	0.8 (0.59–1.09)
Hepatocellular carcinoma	HCC	650 (276, 374)	4.28E-07	0.8	2.14E-07	0.6	NS	1.38 (0.96–1.98)	0.037	1.47 (1.02–2.13)
Acute myeloid leukemia	AML	1079 (929, 150)	9.46E-08	−1	4.73E-08	0.6	NS	1.26 (0.82–1.93)	NA	NA
Lung adenocarcinoma	LUAD	1176 (637, 539)	0.002	1.3	0.001	0.5	NS	0.99 (0.73–1.35)	NS	1.05 (0.77–1.44)
squamous cell carcinoma	LUSC	551 (49, 502)	**0.003**	**2.7**	0.001	0.6	NS	0.98 (0.75–1.29)	NS	124 (0.94–1.63)
Skin cutaneous melanoma	SKCM	2282 (1810, 472)	7.99E-23	0.5	3.99E-23	0.6	NS	0.84 (0.64–1.12)	NA	NA
Ovarian cancer	OV	561 (180, 381)	**1.11E-14**	**1.5**	**5.56E-15**	**0.7**	NS	0.97 (0.74–1.26)	NS	0.95 (0.73–1.24)
Pancreatic adenocarcinoma	PAAD	511 (332, 179)	6.86E-20	−1.1	3.43E-20	0.7	NS	0.69 (0.45–1.06)	NS	0.84 (0.53–1.34)
Pheochromocytoma and Paraganglioma	PCPG	445 (261,184)	3.87E-07	−0.7	1.93E-07	0.6	NS	3.3e-09 (0–Inf)	NS	0 (0–Inf)
Prostate adenocarcinoma	PRAD	798 (297, 501)	NS	1.6	NS	0.5	NS	0.94 (0.25–3.50)	NA	NA
Rectum adenocarcinoma	READ	956 (789,167)	0.001	0.7	4.73E-04	0.6	NS	0.82 (0.37–1.82)	NS	0.67 (0.29–1.53)
Chromophobe renal cell carcinoma	chRCC	179 (114, 65)	NS	0.1	NS	0.5	NS	0.45 (0.05–3.56)	NA	NA
Clear cell renal cell carcinoma	ccRCC	702 (161, 541)	1.25E-15	0.8	6.24E-16	0.7	NS	1.20 (0.89–1.63)	NS	1.19 (0.88–1.61)
Papillary renal cell carcinoma	pRCC	412 (121, 291)	0.005	0.9	0.002	0.6	**2.86E-04**	**2.85** (**1.58–5.17**)	**0.0025**	**2.46** (**1.35–4.49**)
Sarcoma	SARC	265 (2, 263)	NS	0.6	NS	0.6	NS	0.66 (0.43–1.02)	NS	0.71 (0.46–1.12)
Stomach adenocarcinoma	STAD	766 (391, 375)	**2.49E-50**	**4**	**1.25E-50**	**0.8**	NS	1.02 (0.73–1.41)	NS	1.05 (0.76–1.47)
Testicular germ cell tumors	TGCT	517 (361, 156)	**6.59E-73**	**−6.1**	**3.30E-73**	**1.0**	NS	0.76 (0.07–8.38)	NS	0.85 (0.08–9.44)
Thymoma	THYM	121 (2, 119)	NS	5.2	0.047	0.8	NS	0.91 (0.24–3.40)	NS	1.1 (0.3–4.4)
Thyroid carcinoma	THCA	1224 (712, 512)	3.13E-10	−0.6	1.56E-10	0.6	NS	1.84 (0.67–5.08)	NS	1.05 (0.36–3.02)
Uterine corpus endometrial carcinoma	UCEC	731 (177, 554)	NS	1.1	NS	0.5	NS	1.48 (0.97–2.24)	0.0088	1.73 (1.14–2.62)
Uterine carcinosarcoma	UCS	199 (142, 57)	**0.0001**	**3.2**	6.69E-05	0.6	NS	1.57 (0.80–3.08)	NA	NA

All prognostic data extracted from the Kaplan–Meier plotter (KM-Plotter) database were generated using RNA-sequencing pan-cancer datasets. Significant diagnostic and prognostic interests of LINC01087 are highlighted in bold. Abbreviations: AUC, area under the curve; CI, confidence interval; FC, fold change; GTEx, genotype-tissue expression; HR, hazard ratio; KM, Kaplan–Meier; N, normal samples; NA, not available; NS, not significant; OS, overall survival; RFS, relapse-free survival; ROC, receiver operating characteristic; T, tumor samples; TCGA, the cancer genome atlas.

**Table 2:** Significant Associations between LINC01087 Expression and Clinicopathological Characteristics in TCGA and GTEx data.

BC					
Clinicopathological Features		n. of Total Cases	LINC01087 Expression		*p*-Value
			Low	High	
pT	T1-T2	929	453	476	0.04
	T3-T4	180	103	77	
Tumor size	≤2 cm	282	123	159	0.01
	>2 cm	827	433	394	
**TGCT**					
**Clinicopathological Features**		**n. of Total Cases**	**LINC01087** **Expression**		***p*-Value**
Tumor size	≤2 cm	80	41	39	0.05
	>2 cm	58	40	18	

Abbreviations: BC, breast cancer; pN, lymph node stage; pM, metastasis stage; pT, tumor stage; TGCT, testicular germ cell tumors. Fisher’s test.

**Table 3:** LINC01087-miRNA-mRNA network identified in the present study.

lncRNA	10 miRNA Targets Extracted from Analysis 2	18 miRNA Targets Extracted from Analysis 3	10 mRNA Targets Shared between Analyses 1, 2, and 3
LINC01087	hsa-miR-644a	hsa-miR-7-5p	C8orf37
	hsa-miR-1305	hsa-miR-92a-3p	EFNA5
	hsa-miR-4505	hsa-miR-181a-5p	HOOK3
	hsa-miR-3671	hsa-miR-181a-5p, hsa-miR-181b-5p, hsa-miR-181d-5p	IQCG
	hsa-miR-7975	hsa-miR-197-3p	MYOZ3
	hsa-miR-4505	hsa-miR-423-5p	PCP4L1
	hsa-miR-569	hsa-miR-181a-5p, hsa-miR-181b-5p, hsa-miR-98-5p	PLAG1
	hsa-miR-4255	hsa-miR-19a-3p, hsa-miR-19b-3p, hsa-miR-92a-3p	POLI
	hsa-miR-606	hsa-miR-34a-5p, hsa-miR-98-5p	SLC2A3
	hsa-miR-3174	hsa-miR-34a-5p, hsa-miR-98-5p	SNRPA1

In Analysis 2, miRNA targets were evidenced using TCGA; In Analysis 3, miRNA targets were evidenced using DIANA-LncBASE. In both Analyses 2 and 3, mRNA targets of LINC01087-related miRNAs were identified using miRTarBase.

## Data Availability

The datasets used and/or analysed during the current study were published previously as indicated in “Methods” Section. In particular, RNA-seq data were downloaded from the TCGA pancancer 2018 using TCGAbiolinks package in R and from Genotype-Tissue Expression (GTEx) portal (http://www.gtexportal.org/home/datasets, 1 August 2022). The TCGA clinical data were downloaded from TCGA pancancer 2018 using TCGAbiolinks package in R. Survival curves were generated extracting data from TCGA (https://portal.gdc.cancer.gov, 15 September 2022) and were plotted using the online survival analysis tool “Kaplan-Meier Plotter” (http://kmplot.com/analysis/, 2 July 2022) [41,42]. miRNA targets of LINC01087 were identified using both TCGA datasets and DIANA-LncBASE v3 tool (https://diana.e-ce.uth.gr/lncbasev3, 14 October 2022) [45]. mRNA-miRNA interactions were established using miRTarBase (https://mirtarbase.cuhk.edu.cn/~miRTarBase/miRTarBase_2019/php/index.php 20 October 2022) [46].

## Supplementary Materials

The following supporting information can be downloaded at: https://www.mdpi.com/article/10.3390/cancers14235980/s1, Figure S1: Differentially expressed genes correlated with LINC01087 expression in several tissue types extracted from TCGA. Table S1: Non-significant associations between LINC01087 expression and clinicopathological characteristics in TCGA and GTEx data. Table S2: Genes significantly correlated with LINC01087 expression in breast cancer (TGCA); Table S3: Genes significantly correlated with LINC01087 expression in esophageal carcinoma (TGCA); Table S4: Genes significantly correlated with LINC01087 expression in ovarian cancer (TGCA); Table S5: Genes significantly correlated with LINC01087 expression in stomach adenocarcinoma (TGCA); Table S6: Genes significantly correlated with LINC01087 expression in testicular germ cell tumor carcinomas (TGCA); Table S7: GO annotation of genes positively associated with LINC01087 expression in BC, ESCA, OV, STAD and TGCT; Table S8: KEGG pathways associated with LINC01087 expression in BC, ESCA, OV, STAD and TGCT; Table S9: Prediction of subcellular localization of LINC0187 using “lncLocator” database; Table S10: List of the 64 miRNAs significantly correlated with LINC01087 expression in BC, ESCA, OV, STAD and TGCT in TCGA datasets (Analysis 2); Table S11: List of the 31 mRNA targets of LINC01087-related miRNAs that overlapped between Analysis 1 and Analysis 2; Table S12: List of the 20 miRNAs identified to interact with LINC01087 in the DIANA-LncBASE repository (Analysis 3); Table S13: List of the 68 mRNA targets of LINC01087-related miRNAs that overlapped between Analysis 1 and Analysis 3.
