# Supplementary material for: Systematic Investigation of the Diagnostic and Prognostic Impact of LINC01087 in Human Cancers
Source: Cancers (Basel). 2022 Dec 3;14(23):5980. doi: 10.3390/cancers14235980 (PMC9738797; doi:10.3390/cancers14235980)
Supplement: Supplementary file 1 [file cancers-14-05980-s001.zip › cancers-1964643-supplementary.pdf]

cancers

MDPI

Supplementary Materials

Systematic investigation of the diagnostic and prognostic impact of LINC01087 in human cancers

Fatima Domenica Elisa De Palma [1,2,3,4,†], Vincent Carbonnier [3,4,†], Francesco Salvatore [2,5], Guido Kroemer [3,4,6], Jonathan G. Pol [3,4,*,‡] and Maria Chiara Maiuri [1,3,4,‡,*]

[1] Department of Molecular Medicine and Medical Biotechnologies, University of Napoli Federico II, 80131 Napoli, Italy
[2] CEINGE-Biotecnologie Avanzate Franco Salvatore, 80145 Napoli, Italy
[3] Équipe Labellisée par la Ligue Contre le Cancer, Centre de Recherche des Cordeliers, Inserm U1138, Université Paris Cité, Sorbonne Université, Institut Universitaire de France, 75005 Paris, France
[4] Metabolomics and Cell Biology Platforms, Gustave Roussy Cancer Campus, 94805 Villejuif, France
[5] Centro Interuniversitario per Malattie Multigeniche e Multifattoriali e Loro Modelli Animali (Federico II, 80131, Napoli, Tor Vergata, Rome and "G. D'Annunzio", Chieti-Pescara), 80131 Napoli, Italy
[6] Department of Biology, Institut du Cancer Paris CARPEM, Hôpital Européen Georges Pompidou, 75004 Paris, France
* Correspondence: pol_jonathan@yahoo.fr (J.G.P.); chiara.maiuri@crc.jussieu.fr (M.C.M.)

Supplementary Tables

Table S1. Non-significant associations between LINC01087 expression and clinicopathological characteristics in TCGA and GTEx data.

BC

Clinicopathological features		*n. of total cases*	*LINC01087 expression*		*P-value*
			Low	*High*	
Gender	Female	1100	555	545	0.09
	Male	12	3	9	
Age	< 50 years	296	146	150	0.73
	≥ 50 years	801	405	396	
Tumor stage	I-II	815	411	404	0.78
	III-IV	273	135	138	
pN	N0	523	276	247	0.09
	N+	569	271	298	
pM	M0	923	465	458	0.28
	M1	6	14	8	

ESCA

Clinicopathological features		*n. of total cases*	*LINC01087 expression*		*P-value*
			Low	*High*	
Gender	Female	23	13	10	1.00
	Male	140	81	59	
Age	< 50 years	21	15	6	0.24
	≥ 50 years	142	79	63	
Tumor stage	I-II	85	53	32	0.12
	III-IV	57	28	29	
pT	T1-T2	82	49	33	0.61
	T3-T4	64	35	29	
pN	N0	66	42	24	0.18
	N1-N2-N3	78	40	38	
pM	M0	121	67	54	0.14
	M1	8	2	6	
Tumor size	≤ 2cm	119	71	48	0.29
	> 2cm	27	13	14	

OV

Clinicopathological features		*n. of total cases*	*LINC01087 expression*		*P-value*
			Low	*High*	
Gender	Female	381	235	146	1
	Male	0	0	0	

Age	< 50 years	79	54	25	0.23987
	≥ 50 years	294	178	116	
Tumor stage	I-II	24	13	11	0.515346
	III-IV	354	221	133	
pT	T1-T2	262	139	123	0.26
	T3-T4	44	19	25	
pN	N0	NA	NA	NA	NA
	N1-N2-N3	NA	NA	NA	
pM	M0	NA	NA	NA	NA
	M1	NA	NA	NA	
Tumor size	≤ 2cm	NA	NA	NA	NA
	> 2cm	NA	NA	NA	

STAD

Clinicopathological features		*n. of total cases*	*LINC01087 expression*		*P-value*
			Low	*High*	
Gender	Female	134	79	55	0.52
	Male	241	133	108	
Age	< 50 years	26	10	16	0.07
	≥ 50 years	341	197	144	
Tumor stage	I-II	164	89	75	0.59
	III-IV	188	108	80	
pT	T1-T2	99	58	41	0.64
	T3-T4	268	149	119	
pN	N0	111	60	51	0.49
	N1-N2-N3	246	143	103	
pM	M0	330	192	138	0.84
	M1	25	14	11	
Tumor size	≤ 2cm	19	13	6	0.35
	> 2cm	348	194	154	

TGCT

Clinicopathological features		*n. of total cases*	*LINC01087 expression*		*P-value*
			Low	*High*	
Gender	Female	139	81	58	1.00
	Male	0	0	0	
Age	< 50 years	131	77	54	0.72
	≥ 50 years	8	4	4	
Tumor stage	I-II	118	66	52	0.39
	III-IV	14	10	4	
pT	T1-T2	132	77	55	1.00

	T3-T4	6	4	2	
pN	N0	51	34	17	0.52
	N1-N2-N3	13	7	6	
pM	M0	125	72	53	0.14
	M1	8	7	1	

Abbreviations: BC, breast cancer; ESCA, esophageal carcinoma; NA, not available; OV, ovarian cancer; pN, lymph node stage; pM, metastasis stage; pT, tumor stage; STAD, stomach cancer; TGCT, testicular germ cell tumors, Fisher's test,

Table S2. Genes significantly correlated with LINC01087 expression in breast cancer (TGCA).

Gene symbol	Positive R value	P-value	Gene symbol	Positive R value	P-value
POTEKP	0.96	0	RPS20P4	0.44	5.52605E-54
AC093838.1	0.88	0	AC105074.1	0.44	6.06226E-54
POTEI	0.82	3.2585E-268	AC008269.1	0.44	6.25578E-54
GRAMD4P8	0.80	1.5094E-246	AC010746.1	0.44	6.26245E-54
C2orf27A	0.79	3.7805E-241	POTEF	0.44	7.79569E-54
POTEH	0.71	5.7065E-173	RNU6-155P	0.44	8.87003E-54
AC109361.2	0.70	1.2019E-163	RNU6-347P	0.44	9.91852E-54
AC093838.2	0.69	2.9736E-157	AC009948.3	0.44	1.02348E-53
AC103702.1	0.67	1.1757E-146	AC068790.1	0.44	1.05772E-53
LINC02676	0.67	2.7563E-144	AC013439.1	0.44	1.16953E-53
MIR1252	0.67	4.7256E-143	AL079304.1	0.44	1.26811E-53
POTEJ	0.66	1.0889E-141	AL117339.4	0.44	1.55987E-53
AC008679.1	0.66	1.97E-139	RN7SKP74	0.44	1.67098E-53
AC105924.1	0.65	2.1522E-135	RNA5SP372	0.44	1.8863E-53
RNU6-904P	0.64	1.1546E-131	RNU6-652P	0.44	2.03564E-53
C1QTNF7-AS1	0.64	3.6074E-130	ZNF680P1	0.44	3.25899E-53
RPS16P3	0.64	4.1942E-130	NDUFB4P2	0.44	3.50031E-53
MIR7157	0.64	2.8214E-128	MIR3170	0.44	3.95551E-53
AL365436.2	0.63	3.8839E-126	AMD1P1	0.44	4.82322E-53
MIR4460	0.63	6.6005E-126	AC011458.1	0.44	5.07023E-53
REXO1L9P	0.63	2.2553E-122	TCP11X2	0.44	6.00394E-53
AC096733.1	0.62	8.5576E-118	AC012404.2	0.44	6.17481E-53
AC018511.2	0.62	1.8347E-117	RNA5SP294	0.44	6.4889E-53
NBEAP2	0.61	7.0889E-116	LINC01946	0.44	7.2298E-53
AL021068.1	0.61	2.5408E-114	HMGN1P20	0.44	7.79725E-53
AL365436.1	0.61	2.0952E-113	AC006296.1	0.44	1.35717E-52
AL355333.1	0.61	3.44E-113	MTCO1P29	0.43	1.36158E-52
AL354771.1	0.60	8.1242E-112	AC096586.2	0.43	1.39966E-52
IDI1P1	0.60	1.5692E-110	RNU6-1152P	0.43	1.41596E-52
AC008591.1	0.60	4.5317E-110	DUTP8	0.43	1.66284E-52
AC090709.1	0.59	1.6977E-105	AC009716.1	0.43	1.70329E-52
TMEM161BP1	0.59	3.4108E-105	AC245884.3	0.43	1.80276E-52
AC020549.1	0.59	2.5896E-103	AL157902.2	0.43	1.85977E-52
MIR1537	0.58	4.9776E-103	AP005131.1	0.43	1.92492E-52
LRRC57P1	0.58	3.8318E-102	XKRY	0.43	2.08566E-52
LINC02510	0.58	5.9555E-100	SLC7A2-IT1	0.43	2.32398E-52
RNU6-316P	0.57	2.22472E-98	RNU6-1267P	0.43	3.30229E-52
RNU6-148P	0.57	1.27474E-97	PBOV1	0.43	3.55247E-52

RNU6-402P	0.57	2.07655E-97	COX6CP10	0.43	3.69733E-52
RPS20P15	0.57	3.22721E-96	AC007622.2	0.43	4.10376E-52
AC008533.1	0.57	5.28839E-96	AL139396.1	0.43	6.10676E-52
TMEM251P1	0.57	7.69541E-96	RNU6-1112P	0.43	7.98189E-52
ATP5MDP1	0.57	4.22373E-95	SNORD69	0.43	1.142E-51
VN1R53P	0.57	5.45731E-95	PRICKLE2-AS1	0.43	1.23387E-51
AC005741.1	0.56	6.62598E-94	MIR5586	0.43	1.25373E-51
PRKAR2B-AS1	0.56	1.83446E-93	AL450263.1	0.43	1.52582E-51
MIR554	0.56	2.90578E-93	MTRNR2L7	0.43	1.7414E-51
NREP-AS1	0.56	3.00829E-92	ACAP2-IT1	0.43	1.74217E-51
ANKRD30A	0.56	1.6399E-91	AC104446.2	0.43	1.74678E-51
MIR569	0.55	9.277E-91	RNU6-375P	0.43	1.76794E-51
PCDH9-AS4	0.55	1.078E-88	AL136360.1	0.43	1.8155E-51
RNU6-645P	0.55	1.10054E-87	AC004223.1	0.43	2.11204E-51
PTPRT-AS1	0.54	4.83839E-87	ZNF410	0.43	2.14805E-51
AP000462.1	0.54	7.16599E-87	RNU6-625P	0.43	2.33402E-51
AL139003.2	0.54	1.35546E-86	PCDH9-AS2	0.43	2.39072E-51
AC025031.3	0.54	3.19892E-86	RPL7P39	0.43	2.58614E-51
AC097493.1	0.54	5.96972E-86	FDPSP2	0.43	2.77588E-51
TOMM40P4	0.54	1.0363E-85	AC016727.3	0.43	2.84546E-51
NADK2-AS1	0.54	1.84012E-85	AL136981.2	0.43	3.26001E-51
RNU6-578P	0.54	2.04091E-85	MIR376A2	0.43	3.2875E-51
MTCO3P11	0.54	2.28195E-85	AL133259.1	0.43	4.65851E-51
MYB-AS1	0.54	2.68222E-85	TUBAP12	0.43	6.06676E-51
MIR3173	0.54	7.2137E-85	AL354733.2	0.43	6.07892E-51
MTCO2P29	0.54	1.89594E-84	RPL39P14	0.43	7.23221E-51
AP000462.3	0.54	5.61368E-84	RHOA-IT1	0.43	8.06586E-51
AC037193.1	0.54	7.05977E-84	RNU6-654P	0.43	8.15342E-51
RNU4-51P	0.54	7.41498E-84	AC100821.1	0.43	8.24454E-51
AL357568.1	0.54	1.42914E-83	AC009230.1	0.43	9.45708E-51
LDHBP3	0.54	1.5825E-83	AC005183.1	0.43	1.01414E-50
RNU2-13P	0.53	2.52972E-83	AP000893.2	0.43	1.04814E-50
RPS3AP14	0.53	5.52504E-83	SELENOKP2	0.43	1.43569E-50
MIR545	0.53	1.00071E-82	TAS2R20	0.43	1.52508E-50
AC092902.4	0.53	3.14034E-81	AC098869.1	0.43	1.59336E-50
AC239798.2	0.53	7.84425E-81	AC113398.2	0.43	1.62084E-50
AC010261.1	0.53	1.10223E-80	RNU6-724P	0.43	1.72528E-50
AC087427.1	0.53	1.2062E-80	RNY4P20	0.43	1.76417E-50
ANKRD20A5P	0.53	1.60932E-80	AC005785.2	0.43	1.84973E-50
RNU7-11P	0.53	2.00115E-80	POC1B-AS1	0.43	2.37625E-50
SOX5-AS1	0.53	2.87468E-80	AC138150.1	0.43	2.40342E-50
AL358779.1	0.53	4.09464E-80	AC008625.1	0.43	2.55131E-50

AC099313.1	0.52	2.78983E-79	AC015923.1	0.43	2.77732E-50
COPRSP1	0.52	5.37191E-79	AC107896.1	0.43	2.86259E-50
FTX	0.52	1.84318E-78	CNTN4-AS2	0.43	2.87379E-50
MATR3	0.52	2.05027E-78	RNU6-1007P	0.43	3.0334E-50
AC015971.1	0.52	2.70733E-78	AC007000.2	0.43	3.16066E-50
MTND5P14	0.52	3.58744E-78	AP006565.1	0.43	4.00363E-50
MTND4LP9	0.52	4.2651E-78	TFAP2A-AS2	0.43	4.06439E-50
AL133284.1	0.52	5.28131E-78	AC008119.1	0.43	4.31938E-50
MTND4P14	0.52	8.21682E-78	AC114401.1	0.43	4.40485E-50
AC008814.1	0.52	1.54482E-77	RNU6-522P	0.42	5.3168E-50
RNU7-46P	0.52	2.85563E-77	TAS2R4	0.42	5.98532E-50
AL133247.2	0.52	2.86949E-77	SNORD74B	0.42	6.14115E-50
CC2D2B	0.52	2.9524E-77	AL606804.1	0.42	6.65022E-50
CNTN4-AS1	0.52	7.00137E-77	MIR626	0.42	7.24075E-50
AP000462.2	0.52	9.47442E-77	AC037198.1	0.42	7.36609E-50
RNU6-617P	0.51	5.73961E-76	ALG13-AS1	0.42	8.36479E-50
SC4MOP	0.51	6.57989E-76	AC025300.1	0.42	1.05745E-49
MTCO2P11	0.51	8.83501E-76	DOCK4-AS1	0.42	1.11869E-49
LINC01861	0.51	3.8634E-75	OR13G1	0.42	1.20795E-49
MIR4505	0.51	3.97646E-75	SNORD113-9	0.42	1.23595E-49
AC008277.1	0.51	5.50134E-75	RNA5SP28	0.42	1.2442E-49
MIR920	0.51	1.46709E-74	RNU6-1176P	0.42	1.29878E-49
SARNP	0.51	2.31534E-74	AC131212.2	0.42	1.81212E-49
MIR3671	0.51	3.62973E-74	AL359885.1	0.42	2.3927E-49
AC021078.1	0.51	4.67639E-74	AC007695.1	0.42	2.44572E-49
RNU7-38P	0.51	4.70762E-74	AC010368.1	0.42	2.85455E-49
AC004522.1	0.51	2.48744E-73	AC025031.4	0.42	3.2362E-49
RNASEH2B-AS1	0.50	4.67954E-73	LINC02085	0.42	3.35734E-49
GSTCD-AS1	0.50	4.84526E-73	AC002044.3	0.42	3.73169E-49
AC004217.1	0.50	6.40081E-73	AC025576.2	0.42	4.08772E-49
LINC01832	0.50	7.28895E-73	RNU6-469P	0.42	4.99315E-49
RNU4-24P	0.50	1.14242E-72	SAP30L-AS1	0.42	5.07255E-49
RNA5SP307	0.50	2.58271E-72	AC005920.2	0.42	6.29745E-49
RNU6-894P	0.50	2.72109E-72	AC114980.1	0.42	8.41839E-49
AF127577.2	0.50	6.87032E-72	SOCS5P4	0.42	1.15115E-48
SNRPCP11	0.50	9.03613E-72	AC011825.2	0.42	1.15548E-48
AC008868.1	0.50	1.93077E-71	AC078809.1	0.42	1.36233E-48
SIDT1-AS1	0.50	6.16717E-71	AC006254.2	0.42	1.55463E-48
AF127577.6	0.50	7.75122E-71	CBX1P2	0.42	1.57928E-48
AC018816.2	0.50	2.18044E-70	AC005480.1	0.42	1.65847E-48
Z94721.2	0.50	2.65912E-70	AC016949.1	0.42	2.01301E-48
RNU7-169P	0.50	2.81809E-70	CDRT15P3	0.42	2.35342E-48

RN7SKP245	0.50	5.79494E-70	AC093752.2	0.42	2.87816E-48
AC025917.1	0.49	2.20708E-69	AC009754.1	0.42	3.13319E-48
RPS3AP19	0.49	2.32747E-69	HMGN1P12	0.42	3.33576E-48
ENPP7P4	0.49	2.45765E-69	RN7SKP253	0.42	4.32616E-48
UBE2V2P2	0.49	3.81168E-69	AC139792.2	0.42	4.3502E-48
SRGAP3-AS3	0.49	4.92853E-69	ANKRD61	0.42	4.5224E-48
GTF2IP9	0.49	6.26076E-69	Z93943.1	0.42	4.83198E-48
AC064852.1	0.49	1.39803E-68	AL391840.2	0.42	5.12056E-48
ZBTB8OSP1	0.49	1.93087E-68	AC090950.1	0.42	5.40195E-48
LINC02614	0.49	2.07348E-68	AC011586.1	0.42	5.49941E-48
POTEE	0.49	2.985E-68	TAS2R15P	0.42	7.36314E-48
AC007919.1	0.49	3.70502E-68	RNU6ATAC31P	0.42	7.78135E-48
AC114786.3	0.49	4.52611E-68	LNX1-AS1	0.42	1.00571E-47
AC004415.1	0.49	4.68242E-68	RNU6-1275P	0.42	1.02703E-47
NDUFV2	0.49	7.88961E-68	AL139407.1	0.42	1.06463E-47
AC083870.1	0.49	8.26364E-68	AC108471.3	0.42	1.07068E-47
RNU6-876P	0.49	8.69152E-68	AC005702.2	0.42	1.16786E-47
Z83843.1	0.49	9.82577E-68	AC013410.1	0.42	1.20978E-47
AC091117.2	0.49	1.6685E-67	AC004865.1	0.42	1.34752E-47
AC068790.6	0.49	3.10908E-67	ETF1P1	0.41	1.60903E-47
RN7SL314P	0.48	2.02852E-66	DDX3P2	0.41	1.86547E-47
AL391427.1	0.48	3.70775E-66	SPICE1	0.41	1.97282E-47
AL121584.1	0.48	4.64669E-66	LINC01906	0.41	2.08834E-47
AP002991.2	0.48	7.24975E-66	RPL26P27	0.41	2.21337E-47
AC011399.1	0.48	8.15581E-66	AC004223.2	0.41	2.55479E-47
UGT1A3	0.48	8.531E-66	AL137847.1	0.41	2.63159E-47
AC130651.1	0.48	1.24966E-65	RPL35AP14	0.41	3.27569E-47
HOXB-AS2	0.48	1.38319E-65	AL607077.1	0.41	3.56994E-47
AC069549.1	0.48	1.53992E-65	AC011373.1	0.41	3.78563E-47
AC087258.1	0.48	2.18023E-65	RNU6-1165P	0.41	4.20099E-47
RNU6-335P	0.48	3.04833E-65	RNU4-56P	0.41	4.74253E-47
RNA5SP237	0.48	4.49085E-65	LANCL1-AS1	0.41	5.0815E-47
RNU6-905P	0.48	6.22523E-65	TTC3-AS1	0.41	5.15091E-47
AC087260.1	0.48	6.96308E-65	PRSS37	0.41	5.42208E-47
RNU5A-6P	0.48	7.37522E-65	MTND2P2	0.41	5.56598E-47
MIR553	0.48	9.56536E-65	AC117500.2	0.41	5.68576E-47
AC004812.1	0.48	1.36169E-64	MTND5P26	0.41	5.77333E-47
AL163153.1	0.48	1.87713E-64	CSNK1G2P1	0.41	6.1605E-47
OPA1-AS1	0.48	4.3432E-64	NBPF5P	0.41	6.30031E-47
RNY1P14	0.47	1.16334E-63	MIR1284	0.41	7.15407E-47
AC139792.1	0.47	1.8356E-63	AC110813.1	0.41	8.20921E-47
AC091889.1	0.47	2.43712E-63	ANKRD26P4	0.41	8.4371E-47

MIR3134	0.47	2.83038E-63	AL360091.2	0.41	8.67909E-47
Z99129.1	0.47	3.65667E-63	MTND4P32	0.41	8.92578E-47
RAD17P1	0.47	3.83206E-63	PCDHA9	0.41	9.98996E-47
AC010632.3	0.47	4.17792E-63	RNU5E-3P	0.41	1.01342E-46
RNU6-1209P	0.47	5.8338E-63	COX6CP15	0.41	1.02707E-46
RNU4ATAC7P	0.47	8.96955E-63	AC008752.3	0.41	1.12985E-46
AC008147.2	0.47	9.51771E-63	NACAP10	0.41	1.14288E-46
AC002064.2	0.47	1.14321E-62	AC092881.1	0.41	1.27168E-46
RNU6-1005P	0.47	1.32381E-62	RNU6-890P	0.41	1.36536E-46
CDRT15P4	0.47	1.3294E-62	SNORD96B	0.41	1.66158E-46
AC008937.2	0.47	1.37628E-62	THRB-IT1	0.41	1.67832E-46
LINC00894	0.47	1.46986E-62	INTS9-AS1	0.41	1.93008E-46
RPL5P13	0.47	1.76159E-62	AC093752.1	0.41	2.1682E-46
MIR509-1	0.47	2.60502E-62	AL136221.1	0.41	2.54294E-46
PRICKLE2-AS3	0.47	2.92153E-62	AP006248.1	0.41	2.55352E-46
AC068189.2	0.47	4.36649E-62	AC006059.1	0.41	2.5824E-46
AC018797.3	0.47	4.92832E-62	ENPP7P14	0.41	3.01186E-46
RPL31P20	0.47	5.98546E-62	RNU6-206P	0.41	3.25898E-46
JPX	0.47	7.71628E-62	RPL7P26	0.41	3.6326E-46
AL391840.3	0.47	7.8646E-62	AC078882.1	0.41	3.92909E-46
PIGFP2	0.47	1.18736E-61	EIF1AX-AS1	0.41	4.3712E-46
CYP4F30P	0.47	1.20359E-61	ARHGAP16P	0.41	4.7096E-46
AL513185.2	0.47	1.74778E-61	AC063965.2	0.41	7.01352E-46
AC069549.2	0.47	1.77129E-61	AC098798.1	0.41	7.92086E-46
RPS26P5	0.47	3.46954E-61	AP005899.1	0.41	8.55745E-46
MIR606	0.47	3.47304E-61	RNU7-19P	0.41	9.0299E-46
AC139792.3	0.47	3.72859E-61	AC008758.2	0.41	1.09898E-45
AC093416.2	0.47	6.41134E-61	GPR141BP	0.41	1.12964E-45
MIR4526	0.47	6.48118E-61	AL117339.3	0.41	1.32765E-45
MTCO1P11	0.47	7.40838E-61	AC005104.2	0.41	1.32935E-45
SNORA70E	0.46	8.89569E-61	AC010265.1	0.41	1.34189E-45
AF178030.1	0.46	1.33652E-60	AC110760.2	0.41	1.3863E-45
AC034186.2	0.46	2.24793E-60	AC133555.6	0.41	1.60585E-45
PBX1-AS1	0.46	2.33978E-60	RNU6-742P	0.41	1.76225E-45
RNU6-1003P	0.46	2.52198E-60	AC087235.1	0.41	1.84607E-45
ARHGEF38-IT1	0.46	3.59388E-60	AC007673.1	0.41	2.31151E-45
AC091059.1	0.46	3.66108E-60	AC010623.1	0.41	2.53977E-45
MAST4-IT1	0.46	8.68889E-60	AC025918.1	0.41	2.55631E-45
TUBBP3	0.46	9.86969E-60	UHRF2P1	0.41	2.77601E-45
ANKRD20A11P	0.46	1.25026E-59	ADAMTS19-AS1	0.41	2.79056E-45
AC138832.1	0.46	1.71936E-59	LRRC37A17P	0.41	2.91769E-45
AC007684.1	0.46	1.90708E-59	LRRC19	0.41	3.10636E-45

CYP3A7-CYP3A51P	0.46	2.70546E-59	MIR4796	0.41	3.20014E-45
AC037487.2	0.46	3.24593E-59	AC090617.7	0.41	3.36331E-45
AC108063.1	0.46	3.44594E-59	RN7SL418P	0.41	3.49419E-45
AC012358.1	0.46	4.63136E-59	AC022726.2	0.40	3.5701E-45
RHOT1P1	0.46	4.87668E-59	AC009220.2	0.40	3.59159E-45
AC008873.1	0.46	4.94395E-59	ZC3H11A	0.40	3.70404E-45
PRDX3P4	0.46	5.39565E-59	AC092794.1	0.40	4.5256E-45
DNAJC19P1	0.46	7.65135E-59	AC005899.5	0.40	4.92678E-45
AL136115.2	0.46	7.90654E-59	AL591848.3	0.40	5.18423E-45
MIR548AX	0.46	7.97398E-59	AC090617.8	0.40	5.617E-45
GCNT1P1	0.46	8.16642E-59	AC072022.1	0.40	5.94349E-45
TCEAL3-AS1	0.46	1.35621E-58	MTCO3P19	0.40	5.98129E-45
AC098869.2	0.46	1.41159E-58	RAB11FIP1P1	0.40	6.89895E-45
ATG12P2	0.46	1.4587E-58	AC109454.4	0.40	7.61325E-45
MIR346	0.46	1.4667E-58	AC004492.1	0.40	7.88492E-45
AC011451.2	0.46	2.34391E-58	RNU6-181P	0.40	8.45095E-45
ANKHD1	0.46	2.68253E-58	AP002961.1	0.40	8.93774E-45
OR9H1P	0.46	3.17806E-58	GNRHR	0.40	8.98013E-45
AL512283.1	0.46	3.73661E-58	CYP3A7	0.40	1.10188E-44
ANKRD49P1	0.46	3.86095E-58	CENPCP1	0.40	1.34889E-44
RNU6-786P	0.45	6.07455E-58	MIR491	0.40	1.46935E-44
MIR3936HG	0.45	6.44436E-58	KCNMA1-AS2	0.40	1.47537E-44
AF181450.1	0.45	7.18109E-58	AC104365.1	0.40	1.67611E-44
MRPS36P2	0.45	1.3498E-57	FAM185BP	0.40	1.84575E-44
RNF113B	0.45	1.42423E-57	AC018462.1	0.40	1.88617E-44
MTATP6P19	0.45	1.64544E-57	AC068724.4	0.40	1.98586E-44
AC145146.1	0.45	2.83686E-57	AC134050.1	0.40	2.11834E-44
RNU6-750P	0.45	2.94677E-57	RNU6-608P	0.40	2.15048E-44
RNU7-130P	0.45	3.24165E-57	AC019226.1	0.40	2.20388E-44
AC092903.2	0.45	6.15147E-57	AC008770.3	0.40	2.28612E-44
AF064866.1	0.45	9.07765E-57	ANKRD20A21P	0.40	2.60352E-44
AC110760.1	0.45	9.11398E-57	RNU6-586P	0.40	2.81172E-44
AC068790.2	0.45	9.30715E-57	AF064863.1	0.40	2.84355E-44
MTATP6P11	0.45	9.97079E-57	AC097500.1	0.40	3.12442E-44
MIR4774	0.45	1.06949E-56	AC087632.2	0.40	3.26837E-44
RNA5SP168	0.45	1.19607E-56	AC011755.1	0.40	3.47043E-44
AL392023.2	0.45	1.34189E-56	AC010761.3	0.40	3.74055E-44
MIR582	0.45	1.43479E-56	LINC00216	0.40	3.85304E-44
AL049840.6	0.45	1.59782E-56	AC113385.2	0.40	4.28627E-44
AC113340.2	0.45	2.7931E-56	SNRPGP18	0.40	4.53599E-44
LINC00517	0.45	2.96756E-56	AC087854.1	0.40	4.55282E-44
AC005343.4	0.45	3.51377E-56	MIR651	0.40	4.61001E-44

ASTN2-AS1	0.45	4.58326E-56	AL359880.1	0.40	4.74741E-44
MTRNR2L4	0.45	4.92848E-56	OR11L1	0.40	5.69438E-44
AC104126.1	0.45	5.1E-56	AC073569.1	0.40	7.12456E-44
AL451064.2	0.45	5.49905E-56	ANKRD36	0.40	7.71426E-44
AC009365.3	0.45	6.43543E-56	SCYL2P1	0.40	8.02704E-44
TAS2R6P	0.45	7.71192E-56	RNU7-70P	0.40	8.69117E-44
AC010261.2	0.45	1.13891E-55	AC004765.1	0.40	9.28289E-44
AC008147.1	0.45	1.16916E-55	AC024267.5	0.40	1.10174E-43
NCKAP5-IT1	0.45	1.30087E-55	OR6F1	0.40	1.11407E-43
ZFAT-AS1	0.45	1.31718E-55	AL138752.1	0.40	1.15301E-43
AC134669.1	0.45	1.37758E-55	AC007684.2	0.40	1.18231E-43
AP005131.3	0.45	2.02955E-55	AL121652.1	0.40	1.19603E-43
CFAP70	0.45	2.18701E-55	AC108449.1	0.40	1.22001E-43
BBIP1	0.45	2.24861E-55	AC106791.2	0.40	1.29786E-43
AC007619.1	0.45	2.6075E-55	AC018926.3	0.40	1.35932E-43
AC114812.1	0.44	3.23418E-55	RNU6-719P	0.40	1.40836E-43
AC015563.2	0.44	3.89381E-55	RNU6-836P	0.40	1.42259E-43
AC114763.2	0.44	3.90834E-55	MIR4698	0.40	1.53017E-43
AC018511.1	0.44	4.50991E-55	RN7SL495P	0.40	1.77688E-43
LRP1B	0.44	5.49084E-55	AC007435.1	0.40	1.80423E-43
AC090424.1	0.44	6.47956E-55	RNY4P7	0.40	1.93465E-43
AL049780.2	0.44	7.55575E-55	PRICKLE2-AS2	0.40	2.08175E-43
RNU6-1093P	0.44	8.39242E-55	AL031601.1	0.40	2.19538E-43
RNU6-82P	0.44	9.77713E-55	NEGR1-IT1	0.40	2.38653E-43
USP3-AS1	0.44	1.00264E-54	RNU6-310P	0.40	2.53896E-43
MIR3174	0.44	1.07175E-54	RNU6-769P	0.40	2.58764E-43
PCDH9-AS3	0.44	1.12863E-54	AL139805.1	0.40	2.84555E-43
AC114763.1	0.44	1.14194E-54	MTCO3P15	0.40	2.89716E-43
AC117528.1	0.44	1.26738E-54	PRH2	0.40	3.43744E-43
AC012370.1	0.44	1.9672E-54	AC009228.2	0.40	3.63572E-43
OR2X1P	0.44	2.07241E-54	AC106037.3	0.40	3.81554E-43
LYST-AS1	0.44	2.36694E-54	AL161457.2	0.40	4.52901E-43
SNX9-AS1	0.44	2.85425E-54	RORA-AS2	0.40	4.60721E-43
AC092794.2	0.44	2.87507E-54	MIR6809	0.40	4.62257E-43
TH2LCRR	0.44	3.55952E-54	KRT18P7	0.40	4.68472E-43
OR1C1	0.44	4.11848E-54	SNORA70G	0.40	5.11418E-43
AC140481.3	0.44	4.35831E-54	AC069437.1	0.40	5.35909E-43
AC130462.1	0.44	5.12105E-54	EFCAB13	0.40	5.49773E-43
AC005740.3	0.44	5.23653E-54	AC026725.1	0.40	5.5819E-43
			AP000465.1	0.40	6.65912E-43

Table S3. Genes significantly correlated with LINC01087 expression in esophageal carcinoma (TGCA).

Gene symbol	Positive R value	P-value	Gene symbol	Positive R value	P-value
POTEKP	0.95	1.09316E-85	GNAS-AS1	0.50	8.33291E-12
AC093838.1	0.93	5.11746E-73	GABRA2	0.50	8.36137E-12
SNX18P23	0.82	1.68543E-41	AC119868.1	0.50	8.47415E-12
LINC00454	0.81	5.62255E-39	DSCR8	0.50	8.67473E-12
AC119751.6	0.79	1.86856E-35	RNU6-603P	0.50	9.56928E-12
AL731769.2	0.78	5.02683E-35	NF1P11	0.50	9.93537E-12
NEFH	0.78	1.40373E-34	MRGPRX13P	0.50	1.03476E-11
AL772337.2	0.77	3.14327E-33	SLC25A1P3	0.50	1.04975E-11
AL138773.1	0.77	3.62654E-33	AP000553.5	0.50	1.06419E-11
GFY	0.76	5.16646E-32	HPR	0.50	1.16944E-11
MAGEA8	0.75	2.76483E-31	LINC02175	0.50	1.25396E-11
CTSLP8	0.75	4.27748E-31	PRPH2	0.50	1.38312E-11
AL772337.1	0.75	9.35574E-31	AC007996.1	0.50	1.41033E-11
GNG13	0.75	2.76929E-30	AP000428.1	0.50	1.4159E-11
ZSCAN10	0.75	2.86903E-30	NXNL2	0.50	1.41644E-11
AC083836.1	0.74	4.25815E-30	C8orf37	0.50	1.4697E-11
CDH18	0.74	1.16838E-29	C5orf58	0.50	1.5424E-11
BX072579.2	0.74	1.75031E-29	GPAT2	0.50	6.44467E-12
AL445584.2	0.74	3.56406E-29	SUMO2P19	0.50	6.49208E-12
FIGN	0.73	5.93578E-29	MIR548F1	0.50	6.64905E-12
GRAMD4P3	0.73	2.91815E-28	ZNF280B	0.50	6.88797E-12
OLFM3	0.73	4.89372E-28	AP001267.3	0.50	7.26237E-12
SLITRK1	0.73	5.12214E-28	AC019294.2	0.50	7.7951E-12
TAC3	0.73	6.04703E-28	AJ239318.1	0.50	1.54911E-11
GABRG1	0.72	1.20499E-27	KLF2P4	0.50	1.69967E-11
ZFR2	0.72	1.22799E-27	AL355516.1	0.50	1.84856E-11
AC109472.1	0.72	1.5551E-27	GRIN1	0.49	2.17785E-11
ZNF317P1	0.72	1.76359E-27	AC092634.3	0.49	2.18535E-11
KLHL1	0.72	2.07296E-27	GLUD1P3	0.49	2.19774E-11
TNN	0.72	2.16239E-27	H3P9	0.49	2.25843E-11
RASL10A	0.72	2.16712E-27	AP000347.1	0.49	2.33047E-11
AC018558.2	0.72	2.76222E-27	CCDC114	0.49	2.34575E-11
AC103746.1	0.72	3.05669E-27	TPBGL	0.49	2.36581E-11
MIR5692B	0.72	3.69344E-27	MTCO3P10	0.49	2.65801E-11
LINC01297	0.72	3.78484E-27	AC093642.3	0.49	2.76987E-11
H2BU2P	0.72	4.22478E-27	AC012339.1	0.49	2.79169E-11
DPEP3	0.72	4.9596E-27	AL162386.1	0.49	2.79949E-11

PCDH15	0.72	5.59256E-27	PLIN5	0.49	2.81168E-11
TTTY23	0.72	5.89284E-27	MYBL1	0.49	2.95368E-11
FP236240.2	0.72	5.89284E-27	EPOR	0.49	3.1628E-11
CR381670.1	0.71	9.42868E-27	PTH2R	0.49	3.66177E-11
TMEFF2	0.71	1.1208E-26	AP000705.1	0.49	3.74381E-11
AL354919.1	0.71	1.31236E-26	AL157823.2	0.49	3.90351E-11
GRIN3A	0.71	1.52323E-26	EEF1A2	0.49	3.98083E-11
LINC00351	0.71	1.75196E-26	AC068620.3	0.49	4.01319E-11
ASZ1	0.71	1.84376E-26	AC087175.1	0.49	4.25604E-11
SKIDA1	0.71	2.45288E-26	SAA3P	0.49	4.40515E-11
AC092447.5	0.71	3.58466E-26	AP000350.5	0.49	5.03953E-11
AL157931.2	0.71	3.72781E-26	LINC02088	0.49	5.16291E-11
AL353709.1	0.71	4.99546E-26	AL356776.2	0.49	5.20399E-11
AC079799.2	0.70	1.06502E-25	RNU7-123P	0.49	5.21595E-11
ANGPT1	0.70	1.08601E-25	Z99129.3	0.48	5.51672E-11
SEMA3E	0.70	1.1739E-25	GTF2IP3	0.48	5.52647E-11
AL157931.1	0.70	1.99742E-25	PPIAP47	0.48	5.61765E-11
MIR4666A	0.70	2.10511E-25	BX088651.2	0.48	5.63293E-11
LINC01606	0.70	2.2184E-25	OR1X5P	0.48	6.00084E-11
AC099786.2	0.70	2.79294E-25	MAP3K12	0.48	6.42725E-11
AL035603.1	0.70	3.09088E-25	GTF2H4	0.48	6.45195E-11
OR8B5P	0.70	3.12168E-25	DLGAP4-AS1	0.48	6.64374E-11
RNU7-69P	0.70	3.75487E-25	AC073896.4	0.48	6.72719E-11
AL353784.1	0.70	4.08996E-25	AL096701.4	0.48	6.91611E-11
Z84485.1	0.70	4.88851E-25	TUBB8	0.48	7.22132E-11
AL512310.11	0.70	5.55334E-25	AL137001.2	0.48	7.50446E-11
SEMA3D	0.70	6.08486E-25	DDX11L16	0.48	7.81856E-11
AC099786.3	0.69	1.06437E-24	OR8B2	0.48	7.9119E-11
AC135803.1	0.69	1.45155E-24	AC019185.1	0.48	8.16146E-11
H4C10P	0.69	2.01931E-24	DPY19L2	0.48	8.36955E-11
AGGF1P6	0.69	2.2695E-24	PPOX	0.48	8.42236E-11
HSP90AB7P	0.69	2.3077E-24	UFL1-AS1	0.48	1.12179E-10
MAB21L2	0.69	5.27205E-24	AC129492.4	0.48	1.13154E-10
GDPD4	0.68	6.53225E-24	AL160286.2	0.48	1.16444E-10
SNHG31	0.68	6.81337E-24	AL078605.1	0.48	1.17477E-10
AC099786.1	0.68	7.60421E-24	MTND1P4	0.48	1.241E-10
NUS1P2	0.68	7.65841E-24	TEX38	0.48	1.24983E-10
MED15P1	0.68	8.05175E-24	AL139288.2	0.48	1.29651E-10
AL391840.3	0.68	9.34035E-24	CRTC3-AS1	0.48	1.38368E-10
ATXN8OS	0.68	1.54292E-23	AL138831.1	0.48	1.40436E-10
MTCO3P4	0.68	1.83341E-23	GATA3	0.48	1.43757E-10
GABBR2	0.68	1.9054E-23	AC090099.1	0.48	1.44449E-10

PTH2	0.68	2.35374E-23	AL034348.1	0.48	1.47961E-10
HAPLN1	0.68	2.84824E-23	AL035530.2	0.47	1.63038E-10
RTBDN	0.68	3.73421E-23	FRG2B	0.47	1.71602E-10
AC020584.1	0.68	4.28429E-23	AC091133.3	0.47	1.76755E-10
GRAMD4P8	0.68	4.68084E-23	AL024498.1	0.47	1.80905E-10
RNU6-54P	0.67	6.31793E-23	PTCHD3	0.47	1.83172E-10
LINC01231	0.67	6.62681E-23	LINC00664	0.47	1.85986E-10
AC018558.7	0.67	9.38758E-23	OR8G1	0.47	1.87039E-10
AL445207.1	0.67	9.60459E-23	AC118282.2	0.47	1.95739E-10
AL031658.1	0.67	2.48605E-22	MTND5P7	0.47	1.9592E-10
NR2F2	0.67	3.01142E-22	UTS2R	0.47	2.28635E-10
AL391840.2	0.66	4.91725E-22	AL133255.1	0.47	2.31943E-10
CITED2	0.66	5.08729E-22	BMP3	0.47	2.3541E-10
NLGN1	0.66	5.34278E-22	CCDC78	0.47	2.38808E-10
AP000255.1	0.66	6.35991E-22	FRG2JP	0.47	2.53001E-10
AC087477.2	0.66	6.4587E-22	LINC01143	0.47	2.56356E-10
PSMA8	0.66	8.22758E-22	GUSBP10	0.47	2.70456E-10
SNX18P12	0.66	8.251E-22	AL512624.1	0.47	2.75669E-10
LINC02663	0.66	8.35745E-22	SUGT1P4-STRA6LP	0.47	2.9294E-10
GRIA2	0.66	1.36835E-21	MTND6P29	0.47	3.00421E-10
**MED15P4**	0.66	1.50577E-21	OR8B3	0.47	3.19257E-10
SKP1P3	0.66	1.89651E-21	PIFO	0.47	3.33994E-10
AC099520.1	0.65	2.89626E-21	SYT5	0.47	3.37446E-10
AC118282.1	0.65	3.38033E-21	AL645608.4	0.47	3.42846E-10
AC079466.2	0.65	4.13908E-21	AGGF1P10	0.47	3.46215E-10
GBX2	0.65	4.78135E-21	AL591222.2	0.47	3.49052E-10
OR8D1	0.65	5.16127E-21	ERICD	0.47	3.66783E-10
Z98745.2	0.65	6.18735E-21	CROCC2	0.47	3.9032E-10
AC004408.2	0.65	6.98716E-21	MTATP6P10	0.46	4.04044E-10
SNX18P25	0.65	7.52059E-21	AL391650.2	0.46	4.09956E-10
AC092447.2	0.65	7.8462E-21	SLC25A30	0.46	4.42105E-10
MDGA2	0.65	8.66783E-21	DUX4L37	0.46	4.53948E-10
SPACA6P-AS	0.65	8.74371E-21	AC020907.5	0.46	4.58452E-10
GRIA4	0.65	9.70669E-21	ELK2BP	0.46	5.09112E-10
POTEH	0.65	1.05948E-20	CILP2	0.46	5.62452E-10
AC005006.1	0.64	1.86706E-20	AC130650.2	0.46	5.8173E-10
LINC00452	0.64	1.92668E-20	GJD2	0.46	6.06647E-10
AL451064.1	0.64	2.14049E-20	AL512310.10	0.46	6.15505E-10
LINC02571	0.64	2.32026E-20	HERC2P8	0.46	6.2085E-10
MDM1	0.64	2.61446E-20	EID3	0.46	6.32517E-10
HABP4	0.64	2.97611E-20	LRRN3	0.46	6.36802E-10

CAVIN2-AS1	0.64	3.5762E-20	SBNO1-AS1	0.46	7.2122E-10
DNM3	0.64	3.67607E-20	LINC01695	0.46	7.38592E-10
AC099654.5	0.64	3.75595E-20	AL929601.2	0.46	7.83783E-10
SLC2A11	0.64	3.77524E-20	LINC01673	0.46	7.87122E-10
CDH12P4	0.64	4.0384E-20	BANF1P2	0.46	8.27436E-10
LHX8	0.64	4.38174E-20	ZNF467	0.46	8.45871E-10
HMGB1P46	0.64	5.6998E-20	AC092720.2	0.46	9.20473E-10
AP000553.7	0.64	5.83176E-20	FOXN4	0.46	9.26626E-10
DSCR10	0.64	6.1018E-20	DDX43	0.46	9.31653E-10
CATIP	0.64	6.46267E-20	TLCD5	0.46	9.35509E-10
AL731769.1	0.64	6.47645E-20	AP000446.1	0.46	9.56618E-10
GLRB	0.64	7.79435E-20	CT45A10	0.46	9.97483E-10
AL590491.2	0.64	8.30469E-20	AC084876.2	0.45	1.07743E-09
TP53TG3HP	0.63	9.87126E-20	GDF9	0.45	1.08102E-09
AC025674.1	0.63	9.98026E-20	SAMD11	0.45	1.1043E-09
RNU6-334P	0.63	1.05051E-19	AC118282.4	0.45	1.1628E-09
MIR1915HG	0.63	1.05664E-19	FOXI1	0.45	1.27356E-09
AC142116.2	0.63	1.28544E-19	PLA2G12AP1	0.45	1.28504E-09
CDH19	0.63	1.34602E-19	FAM238C	0.45	1.30211E-09
FTLP12	0.63	1.53245E-19	LIX1	0.45	1.47511E-09
AC068205.1	0.63	1.587E-19	AP003900.1	0.45	1.47728E-09
NTSR2	0.63	2.97236E-19	AC129850.1	0.45	1.48898E-09
LINC02593	0.63	3.22847E-19	RNU6-448P	0.45	1.53366E-09
LINC02689	0.63	3.41208E-19	GPR37	0.45	1.55349E-09
SYPL2	0.62	5.12671E-19	MTATP6P8	0.45	1.5821E-09
AC126915.2	0.62	6.41854E-19	DSCR4-IT1	0.45	1.59162E-09
DNM3-IT1	0.62	7.56165E-19	AL138831.3	0.45	1.60407E-09
AL356390.1	0.62	8.10325E-19	AC091770.1	0.45	1.73073E-09
AL671883.1	0.62	8.4983E-19	AL359697.1	0.45	1.78138E-09
OR8A1	0.62	9.24851E-19	AC017100.1	0.45	1.81832E-09
AC013287.1	0.62	9.56596E-19	TAS2R14	0.45	1.90431E-09
CTCFL	0.62	9.68994E-19	LINC00442	0.45	1.91324E-09
GUSBP12	0.62	1.1817E-18	AC023347.1	0.45	1.96533E-09
LDHAL6A	0.62	1.27727E-18	C10orf88B	0.45	2.05841E-09
AL353726.1	0.62	1.29389E-18	MRPS17P1	0.45	2.23227E-09
TSBP1-AS1	0.62	1.5853E-18	LINC01581	0.45	2.28609E-09
AC016885.3	0.62	1.69284E-18	RPS6P8	0.45	2.2932E-09
NF1P4	0.62	1.74718E-18	AC010547.5	0.45	2.32182E-09
OSTCP2	0.62	2.08955E-18	MTND5P6	0.45	2.37814E-09
AACSP1	0.62	2.11729E-18	AC140113.4	0.45	2.40361E-09
PANX3	0.62	2.26354E-18	PRAME	0.45	2.45556E-09
KCNV1	0.62	2.32944E-18	AL662795.2	0.45	2.45741E-09

AL022329.3	0.61	3.48548E-18	ZNF608	0.45	2.49344E-09
AC107464.2	0.61	4.31333E-18	AP001476.1	0.45	2.65585E-09
CFAP77	0.61	4.31582E-18	TP53TG3D	0.44	2.74595E-09
AC079987.1	0.61	4.77561E-18	RPEP4	0.44	2.82738E-09
AC140125.1	0.61	4.92962E-18	HERC2P5	0.44	2.83006E-09
AC096543.2	0.61	5.36473E-18	DSCR4	0.44	2.87294E-09
AL160254.1	0.61	5.93896E-18	GRAMD1C	0.44	2.95799E-09
AC055788.2	0.61	6.88314E-18	AC007240.2	0.44	3.04806E-09
LUNAR1	0.61	7.15355E-18	AL355297.3	0.44	3.20734E-09
ERBB4	0.61	8.30064E-18	AC002091.1	0.44	3.40853E-09
LINC01140	0.61	8.51123E-18	HES7	0.44	3.42575E-09
AP000428.2	0.61	8.80707E-18	PNMA3	0.44	3.7477E-09
AL713851.1	0.61	9.72471E-18	CR392039.3	0.44	3.76847E-09
H2BU1	0.60	1.28402E-17	FBXO16	0.44	3.84836E-09
AC015574.1	0.60	1.4688E-17	MTND4P4	0.44	3.93892E-09
RN7SL221P	0.60	1.55303E-17	CR381653.2	0.44	3.9403E-09
TTC39C-AS1	0.60	1.93427E-17	AC027097.1	0.44	4.15995E-09
OR8B7P	0.60	2.03436E-17	ANKRD34A	0.44	4.29401E-09
CRYZ	0.60	2.07421E-17	AC010336.2	0.44	4.43498E-09
SNORD114-25	0.60	2.1364E-17	UFL1	0.44	4.6043E-09
LINC01621	0.60	2.17901E-17	AC245452.3	0.44	4.71441E-09
H4C5	0.60	3.50327E-17	AC010240.1	0.44	5.22516E-09
LINC01625	0.60	4.09763E-17	PABPC1L	0.44	5.50081E-09
AC010336.5	0.60	4.40215E-17	COL26A1	0.44	5.57359E-09
MIR663AHG	0.60	5.22915E-17	ANKRD26P1	0.44	5.76377E-09
AL451064.2	0.60	5.36826E-17	LINC00540	0.44	5.90415E-09
AC009802.1	0.60	5.44089E-17	AC233702.9	0.44	5.97425E-09
AL160391.1	0.59	5.52861E-17	WASF5P	0.44	6.0534E-09
CADM2	0.59	5.54083E-17	CAPS2	0.44	6.31101E-09
BX664608.1	0.59	7.30647E-17	OR8G3P	0.43	6.85043E-09
AC051618.1	0.59	8.37283E-17	DUSP19	0.43	7.4985E-09
MAPT-IT1	0.59	8.83745E-17	AC107068.1	0.43	8.33869E-09
LINC00535	0.59	9.44081E-17	ZSCAN1	0.43	8.5435E-09
AC104667.1	0.59	1.02598E-16	AL137157.1	0.43	8.60476E-09
AL022324.3	0.59	1.09061E-16	RNU6-925P	0.43	8.65048E-09
AC090578.2	0.59	1.23751E-16	ADGRL2	0.43	8.66317E-09
OR8Q1P	0.59	1.31281E-16	AL138831.2	0.43	8.99399E-09
PPP3R2	0.59	1.42453E-16	DEFB109D	0.43	9.29277E-09
KCNQ2	0.59	2.14971E-16	OR8B10P	0.43	9.73241E-09
RNU6-909P	0.59	2.20157E-16	LINC02090	0.43	1.00915E-08
SYCE1	0.59	2.25434E-16	CTXN1	0.43	1.05418E-08
AP000553.4	0.59	2.31281E-16	AL645940.1	0.43	1.06224E-08

EDIL3-DT	0.58	2.44837E-16	PKHD1	0.43	1.07199E-08
UBR5-AS1	0.58	2.47022E-16	AC138649.1	0.43	1.10613E-08
MEIOC	0.58	2.6501E-16	XAGE1A	0.43	1.15682E-08
AC019193.3	0.58	3.025E-16	LINC02505	0.43	1.15813E-08
AL929601.1	0.58	3.92156E-16	B4GALT6	0.43	1.16905E-08
AP000553.2	0.58	4.4181E-16	ANKRD20A21P	0.43	1.20262E-08
THSD7A	0.58	4.81233E-16	LINC02521	0.43	1.24143E-08
AP000350.8	0.58	4.81508E-16	MED15P6	0.43	1.26741E-08
AC010735.2	0.58	5.12559E-16	HCG18	0.43	1.3173E-08
SLC11A2	0.58	5.40885E-16	USP32P3	0.43	1.33123E-08
AL365259.1	0.58	5.88564E-16	AC018865.2	0.43	1.33578E-08
AC096736.3	0.58	7.11646E-16	PRSS43P	0.43	1.33675E-08
LINC01600	0.58	7.30846E-16	LINC01839	0.43	1.37761E-08
TATDN2P3	0.58	7.86472E-16	AL135791.1	0.43	1.3866E-08
L29074.1	0.58	9.33909E-16	H2BC15	0.43	1.45916E-08
CDH10	0.57	1.113E-15	AC237221.2	0.42	1.58085E-08
GABRG2	0.57	1.11353E-15	AC090061.1	0.42	1.59591E-08
CCDC151	0.57	1.17085E-15	AC097462.3	0.42	1.63645E-08
AC009126.1	0.57	1.23389E-15	AL133334.1	0.42	1.64585E-08
LINC01448	0.57	1.35066E-15	AC068134.1	0.42	1.65393E-08
AC069113.1	0.57	1.73219E-15	PSLNR	0.42	1.72569E-08
SNX18P24	0.57	2.08534E-15	AC138776.1	0.42	1.73784E-08
AP000553.6	0.57	2.6064E-15	KIF5A	0.42	1.74068E-08
AC073389.3	0.57	2.66823E-15	PLPPR3	0.42	1.76833E-08
AC097532.3	0.57	2.8895E-15	RASGEF1C	0.42	1.85272E-08
SELENOV	0.57	2.95553E-15	AL590064.1	0.42	2.02872E-08
PROKR2	0.57	3.10912E-15	AL353729.2	0.42	2.17491E-08
SGCG	0.57	3.13223E-15	PPIAP35	0.42	2.20791E-08
MRGPRX12P	0.57	3.36711E-15	GSG1L	0.42	2.21921E-08
AC119751.4	0.57	3.58375E-15	FAM216A	0.42	2.24118E-08
NEFMP1	0.57	3.89184E-15	AC119751.3	0.42	2.30169E-08
MTCO2P10	0.56	4.15791E-15	PRSS44P	0.42	2.32075E-08
AL353743.2	0.56	4.58526E-15	LINC01901	0.42	2.38756E-08
SEMA3A	0.56	5.11304E-15	TSPEAR-AS1	0.42	2.4026E-08
H2AC7	0.56	6.28076E-15	NF1P8	0.42	2.42358E-08
FGF12-AS3	0.56	6.49267E-15	AC136431.1	0.42	2.47731E-08
MAGEA4-AS1	0.56	6.68416E-15	H2AW	0.42	2.55361E-08
MYO18B	0.56	7.1625E-15	FIGNL2-DT	0.42	2.63178E-08
AL645608.6	0.56	7.70292E-15	RB1-DT	0.42	2.65553E-08
OR6L1P	0.56	8.04754E-15	AL355490.2	0.42	2.66158E-08
RPA2P1	0.56	8.32102E-15	AC024361.2	0.42	2.6674E-08
OR8B12	0.56	8.57164E-15	WDR17	0.42	2.70563E-08

AC110813.1	0.56	8.9811E-15	NLGN1-AS1	0.42	2.72869E-08
MAGEA8-AS1	0.56	9.0834E-15	LL22NC03-63E9.3	0.42	2.72893E-08
ZNF280A	0.56	9.69015E-15	DNAJC27-AS1	0.42	2.75494E-08
NR5A2	0.56	9.7175E-15	AC011498.4	0.42	2.77681E-08
AC140168.1	0.56	1.30013E-14	HNRNPDLP4	0.42	2.83126E-08
AC093326.1	0.56	1.37936E-14	AL132765.2	0.42	2.87746E-08
AL589823.1	0.55	1.50091E-14	METTL7A	0.42	2.88973E-08
P2RX2	0.55	1.52734E-14	IGHV7-40	0.42	2.90721E-08
GTSF1	0.55	1.6376E-14	AC105118.1	0.42	2.93486E-08
MTND4LP32	0.55	2.34457E-14	C10orf88	0.42	3.09E-08
KCNH5	0.55	2.35474E-14	RAD21L1	0.42	3.12935E-08
AC010735.1	0.55	2.63034E-14	H2AB3	0.42	3.14185E-08
AP001425.1	0.55	2.79166E-14	AL137003.1	0.42	3.36572E-08
RPS3AP24	0.55	3.26254E-14	AL138828.1	0.42	3.38533E-08
OXTR	0.55	3.71072E-14	AL008721.1	0.42	3.42204E-08
LINC01351	0.55	4.5368E-14	F11-AS1	0.42	3.44244E-08
TYW3	0.55	4.83664E-14	PDGFC	0.41	3.78972E-08
USP24P1	0.55	4.93703E-14	LBHD1	0.41	3.84795E-08
MTCYBP5	0.55	5.0682E-14	RNA5SP532	0.41	3.8892E-08
LCA5	0.55	5.06914E-14	EDIL3	0.41	4.01429E-08
AP000916.1	0.55	5.25834E-14	AC105339.5	0.41	4.39217E-08
AP000553.1	0.55	5.29325E-14	RN7SKP234	0.41	4.51628E-08
POLI	0.54	5.43972E-14	MTCYBP29	0.41	4.53015E-08
Z95115.1	0.54	5.59383E-14	AC010632.3	0.41	4.65478E-08
AC092967.1	0.54	6.03972E-14	ACOT6	0.41	4.89543E-08
OR8D2	0.54	6.38195E-14	SS18L1	0.41	4.91094E-08
OR8B6P	0.54	6.46818E-14	RGL3	0.41	5.11228E-08
FRG1GP	0.54	8.62479E-14	AC233702.1	0.41	5.33357E-08
ZNF663P	0.54	9.7037E-14	AP000705.2	0.41	5.33411E-08
OR8A3P	0.54	1.05007E-13	AL157414.4	0.41	5.45139E-08
AL031686.1	0.54	1.08209E-13	KAT14	0.41	5.49505E-08
PCDH10	0.54	1.0889E-13	AL034550.3	0.41	5.69663E-08
LINC00308	0.54	1.11468E-13	FRG1FP	0.41	5.86582E-08
NAT8L	0.54	1.12708E-13	SLC2A3	0.41	5.89207E-08
KCNN1	0.54	1.13298E-13	HESX1	0.41	5.97134E-08
AC011029.1	0.54	1.50923E-13	IGLVIVOR22-1	0.41	6.17415E-08
AC087499.2	0.54	1.53139E-13	MIR3153	0.41	6.22725E-08
AC092656.1	0.54	1.53667E-13	AC138625.1	0.41	6.40278E-08
FRG2DP	0.54	1.66986E-13	AC078916.1	0.41	6.55089E-08
AL662791.1	0.54	1.76932E-13	AL132655.2	0.41	6.59612E-08
AC119751.1	0.54	1.77803E-13	LINC00466	0.41	6.74568E-08
GBX1	0.53	2.10204E-13	SAPCD2P4	0.41	6.96613E-08

AC096555.1	0.53	2.11407E-13	HCG15	0.41	7.03774E-08
SERPINH1P1	0.53	2.15603E-13	AC027117.1	0.41	7.06623E-08
AC012409.2	0.53	2.1917E-13	ASNSP1	0.41	7.18749E-08
AL137802.2	0.53	2.32558E-13	ANKRD53	0.41	7.19604E-08
KCNC3	0.53	2.32669E-13	RN7SKP169	0.41	7.24751E-08
POM121L2	0.53	2.34418E-13	CATSPER2P1	0.41	7.27963E-08
AL645608.2	0.53	2.7307E-13	AP001626.1	0.41	7.4297E-08
DPPA2	0.53	3.14551E-13	RN7SL268P	0.41	7.43772E-08
AL450332.1	0.53	4.19791E-13	AL391152.1	0.41	7.45483E-08
THRAP3P3	0.53	4.40401E-13	H2BC9	0.41	7.5235E-08
CCM2L	0.53	4.5428E-13	AL031777.2	0.41	7.53967E-08
AC099754.1	0.53	4.8033E-13	SLC27A6	0.41	7.81484E-08
SPATA31C2	0.53	5.43119E-13	AC136944.3	0.41	8.01862E-08
AL353726.2	0.53	5.62372E-13	MST1	0.41	8.0418E-08
AL353740.1	0.53	5.98078E-13	AL513478.4	0.41	8.11344E-08
H3C5P	0.52	6.84989E-13	AL451142.1	0.40	8.28564E-08
CNTNAP3P1	0.52	7.05627E-13	ATP6V0E2-AS1	0.40	8.46946E-08
AC004925.1	0.52	7.87799E-13	CHRNE	0.40	8.88013E-08
AC093875.1	0.52	7.93676E-13	CDC37P2	0.40	8.89273E-08
PAGE5	0.52	8.08587E-13	ZNF780A	0.40	9.05499E-08
AL353572.3	0.52	8.88137E-13	BAIAP3	0.40	9.26405E-08
TNNI3	0.52	1.03724E-12	LINC01237	0.40	9.2714E-08
CLDN6	0.52	1.06228E-12	AC023824.6	0.40	9.28787E-08
COX6A2	0.52	1.12853E-12	EXTL2	0.40	9.59386E-08
PHF2P2	0.52	1.19633E-12	AC138207.3	0.40	9.73464E-08
GRAMD4P2	0.52	1.40801E-12	NEK2P1	0.40	1.00239E-07
CEACAMP10	0.52	1.68646E-12	LINC01202	0.40	1.00652E-07
SLITRK6	0.52	1.68919E-12	PCMTD2	0.40	1.03135E-07
AL591438.2	0.52	1.72274E-12	TMEM247	0.40	1.04219E-07
MTND2P6	0.52	1.77646E-12	AC023141.13	0.40	1.0432E-07
CLUL1	0.52	1.98682E-12	AC009005.1	0.40	1.06822E-07
AC099654.4	0.51	2.04448E-12	AL590096.1	0.40	1.07701E-07
CFHR4	0.51	2.05823E-12	AC105129.2	0.40	1.08919E-07
AP006261.1	0.51	2.06494E-12	CDRT15P8	0.40	1.09781E-07
VN1R34P	0.51	2.08136E-12	AC097532.2	0.40	1.1098E-07
MYOZ3	0.51	2.15975E-12	RALY-AS1	0.40	1.11449E-07
AL138885.2	0.51	2.23064E-12	RNU6-677P	0.40	1.12152E-07
FAR2P1	0.51	2.26887E-12	AC087393.1	0.40	1.12434E-07
AC087499.5	0.51	2.35386E-12	SLC22A31	0.40	1.14458E-07
LINC02600	0.51	2.37488E-12	TUBA3C	0.40	1.16921E-07
NF1P7	0.51	2.65679E-12	AP002761.3	0.40	1.17989E-07
MAP3K15	0.51	2.66823E-12	PRR36	0.40	1.18733E-07

RARRES2P10	0.51	2.8434E-12	POTEG	0.40	1.19686E-07
AC008738.1	0.51	2.91639E-12	C2orf15	0.40	1.20435E-07
SPATA31C1	0.51	3.02202E-12	EFNA5	0.40	1.21861E-07
AL009179.2	0.51	3.37603E-12	AC083843.1	0.40	1.25414E-07
SPATA31E1	0.51	3.50251E-12	H2BC8	0.40	1.25615E-07
ZNF391	0.51	3.92095E-12	AC112229.2	0.40	1.30556E-07
FXYD7	0.51	4.10528E-12	CCDC144NL-AS1	0.40	1.32491E-07
GRB14	0.51	4.22088E-12	CFAP61-AS1	0.40	1.34888E-07
FAR2P4	0.51	4.75636E-12	PACRG	0.40	1.36811E-07
AC007663.3	0.51	4.79377E-12	XAGE1B	0.40	1.38651E-07
STAG3	0.51	5.10101E-12	AC022387.2	0.40	1.39631E-07
AP001804.1	0.51	5.23803E-12	H2BC19P	0.40	1.46942E-07
NR2F2-AS1	0.51	5.58923E-12	KLHL7-DT	0.40	1.58765E-07
LINC00314	0.51	5.77569E-12	AC020934.1	0.40	1.67956E-07
SMC2-AS1	0.51	6.11304E-12	NRBF2P2	0.40	1.70323E-07
			AC007391.1	0.40	1.74439E-07

Table S4. Genes significantly correlated with LINC01087 expression in ovarian cancer (TCGA).

Gene symbol	Positive R value	P-value	Gene symbol	Positive R value	P-value
AC093838.1	0.91	2.5519E-149	AC009597.1	0.48	1.54889E-23
MED15P4	0.81	3.30589E-91	RN7SL863P	0.48	1.58438E-23
AC006512.2	0.68	1.55423E-52	TTC4P1	0.48	1.81225E-23
ATP6V1G1P7	0.67	8.25797E-52	AP000619.1	0.48	2.00541E-23
LINC00529	0.67	8.25797E-52	ANKRD63	0.48	2.93902E-23
RNU6-295P	0.67	8.25797E-52	FER1L6	0.48	3.4718E-23
AP002991.2	0.67	8.25797E-52	PNMA5	0.47	2.36614E-22
SIGLECL1	0.67	1.39398E-51	LINC01854	0.47	4.61838E-22
AC007846.1	0.67	1.77284E-51	MIR4255	0.47	6.15206E-22
RPL6P12	0.67	1.41786E-50	POTEE	0.46	2.66032E-21
AP003031.1	0.67	3.7339E-50	HOXC-AS3	0.46	2.9141E-21
AC004835.1	0.67	4.87028E-50	C10orf71-AS1	0.45	8.55858E-21
MYL12BP1	0.66	9.41662E-49	SLC25A24P2	0.45	1.08936E-20
LINC01682	0.65	1.53134E-47	LHFPL3-AS2	0.45	1.22293E-20
DHRS2	0.65	2.73486E-47	LINC01686	0.45	1.5483E-20
AC007846.2	0.64	3.31409E-45	FAM83C	0.45	1.98928E-20
LINC02706	0.64	7.13851E-45	KRT18P59	0.45	2.31439E-20
AL592464.3	0.64	1.75464E-44	SNX18P7	0.45	4.4976E-20
AFG3L2P1	0.63	6.74738E-44	AP002008.1	0.45	4.6822E-20
AL117340.1	0.63	6.82527E-44	AC090735.1	0.45	5.04702E-20
AL592464.1	0.62	5.5581E-42	Z98259.2	0.45	5.32037E-20
KRT72	0.62	3.27579E-41	GPR83	0.44	6.98501E-20
POTEKP	0.61	7.88627E-41	AC091133.4	0.44	7.81771E-20
HOXC12	0.61	1.3117E-40	AC108727.2	0.44	8.76905E-20
AC111188.2	0.61	6.41932E-40	UPK2	0.44	1.45276E-19
AC021006.1	0.59	1.97816E-37	KBTBD13	0.44	1.65193E-19
PIP5K1P2	0.59	1.46754E-36	FNTA	0.44	2.05141E-19
AC000036.1	0.59	1.82759E-36	AC012531.6	0.44	2.1494E-19
LINC01120	0.58	2.31104E-35	OR7E101P	0.44	2.69909E-19
C15orf62	0.58	2.73046E-35	VIPR2	0.44	3.25409E-19
AL606500.1	0.58	4.02016E-35	AC016168.2	0.44	3.94727E-19
TERB1	0.58	5.99555E-35	RN7SKP200	0.44	4.90634E-19
C9orf129	0.57	8.59279E-35	TMPOP2	0.43	5.55929E-19
TBC1D8-AS1	0.57	4.70651E-34	GPS2P2	0.43	7.04072E-19
AC007993.1	0.57	8.04766E-34	AC091133.5	0.43	7.11252E-19
AL513478.2	0.56	1.44061E-32	AC124947.2	0.43	9.33711E-19
POMK	0.56	1.61221E-32	AP000692.2	0.43	1.03565E-18
ARMS2	0.55	2.20195E-31	SSBP3P2	0.43	1.03606E-18

LINC01623	0.54	2.2104E-30	LINC01647	0.43	1.1827E-18
AL109809.2	0.54	6.2863E-30	ISCA1P4	0.43	1.4958E-18
AC016168.1	0.54	1.24898E-29	AL590068.2	0.43	1.75655E-18
LINC01499	0.53	1.38783E-29	RPL4P1	0.43	1.76205E-18
AC009477.1	0.53	5.27622E-29	TEX14	0.43	3.04114E-18
BRDT	0.53	1.15564E-28	AC012531.4	0.43	3.07058E-18
AC112243.1	0.52	3.88188E-28	KRT9	0.42	3.98712E-18
TRAV8-2	0.52	7.65978E-28	OR7E96P	0.42	5.46461E-18
MIR196A2	0.52	2.28855E-27	AL139384.2	0.42	6.97202E-18
LINC01475	0.52	3.17157E-27	HOOK3	0.42	7.83489E-18
CALCA	0.51	4.10743E-27	AC006460.1	0.42	7.91889E-18
AL138686.2	0.51	5.87869E-27	HYLS1	0.42	9.40279E-18
AC005342.2	0.51	9.59796E-27	CYP4F26P	0.42	9.8183E-18
TREML5P	0.51	1.6414E-26	YAP1	0.42	1.13877E-17
AC079414.2	0.51	1.73003E-26	MRPS21P5	0.42	1.20205E-17
SCP2D1-AS1	0.51	2.19581E-26	NRXN2-AS1	0.42	1.81972E-17
TEX55	0.51	2.6328E-26	AL391883.1	0.42	2.2848E-17
AC091179.1	0.51	4.32454E-26	PRAMEF19	0.42	2.49784E-17
PATE2	0.50	7.06663E-26	RNU7-187P	0.41	2.93072E-17
HOXC11	0.50	9.45616E-26	AC002115.1	0.41	3.07548E-17
AC111149.2	0.50	1.00934E-25	MIR138-2	0.41	8.98595E-17
AC064869.1	0.50	2.12053E-25	SPATA31E1	0.41	9.37168E-17
RNU7-159P	0.50	3.46128E-25	PDSS1P2	0.41	1.11644E-16
Z94160.1	0.50	4.53726E-25	AC073869.6	0.41	1.22834E-16
AP001042.3	0.50	5.19444E-25	GJA8	0.40	3.38565E-16
CLCN1	0.49	1.36212E-24	RGS8	0.40	3.8684E-16
AL391358.1	0.49	1.37433E-24	NF1P8	0.40	3.97383E-16
AC021006.2	0.49	1.56962E-24	HOTAIR	0.40	4.53704E-16
AC008443.5	0.49	2.49791E-24	AC106793.1	0.40	4.57198E-16
NUTM1	0.49	5.17771E-24	RNU6-781P	0.40	6.46058E-16
AC004147.4	0.49	6.62166E-24	AP001486.3	0.40	9.43728E-16
AC087632.1	0.48	8.75141E-24	SMOC1	0.40	9.94388E-16
			MEIOC	0.40	1.09602E-15

Table S5. Genes significantly correlated with LINC01087 expression in stomach adenocarcinoma (TCGA).

Gene symbol	Positive R value	P-value	Gene symbol	Positive R value	P-value
POTEKP	0.93	5.2227E-160	AL590560.3	0.47	2.78897E-22
AC093838.1	0.90	2.7416E-137	F7	0.47	2.87164E-22
MED15P4	0.84	1.1074E-101	AL136038.3	0.47	3.00737E-22
AL022329.3	0.80	4.92878E-85	AC091153.3	0.47	3.17414E-22
NF1P8	0.79	9.29058E-80	CCDC8	0.47	3.18856E-22
AP005205.3	0.76	1.19219E-70	LINC01518	0.47	3.2261E-22
AC018865.2	0.75	1.41379E-69	AL049543.1	0.47	3.81352E-22
AC090907.2	0.75	2.66094E-69	AC012467.1	0.47	4.60134E-22
AC135068.11	0.75	1.00826E-68	PDE6B	0.47	5.14239E-22
AC183088.4	0.75	2.08581E-68	LINC01012	0.47	5.44214E-22
LINC01580	0.74	1.68429E-67	AC243965.2	0.47	5.89769E-22
FAR2P1	0.74	1.67667E-66	AL109918.1	0.47	6.43541E-22
NF1P9	0.74	8.06921E-66	FO082796.1	0.47	6.55031E-22
SOCS2P2	0.74	2.845E-65	AC099661.1	0.47	6.72162E-22
LINC02203	0.73	5.63804E-64	AC093635.1	0.47	7.17868E-22
AL353726.2	0.72	7.75175E-62	RNU6-53P	0.47	7.33577E-22
KLF2P4	0.72	1.66255E-61	AF186192.2	0.47	7.44838E-22
AL451142.1	0.72	5.07395E-61	RN7SKP76	0.47	7.66024E-22
AURKC	0.72	1.66353E-60	IGHVII-46-1	0.47	9.5614E-22
AC037471.1	0.72	1.89607E-60	AL590062.1	0.47	1.00695E-21
NF1P1	0.72	4.27314E-60	AL353708.3	0.47	1.12836E-21
AC134980.1	0.72	4.57659E-60	LINC01740	0.47	1.12836E-21
BMP6P1	0.71	8.38813E-60	ANKRD26P3	0.47	1.15064E-21
AC103996.3	0.71	2.84535E-58	AL049646.1	0.47	1.3634E-21
LINC01193	0.70	1.54308E-57	AC244100.3	0.47	1.51406E-21
AC131180.1	0.70	6.25024E-57	LINC00355	0.46	1.78626E-21
AC073578.4	0.70	6.6343E-57	AC026471.5	0.46	1.83592E-21
AC025884.2	0.70	3.65351E-56	AL009176.1	0.46	1.87517E-21
CENPIP1	0.70	1.30932E-55	TSPEAR-AS2	0.46	2.03039E-21
AL355516.1	0.69	4.38803E-55	AL445228.2	0.46	2.16817E-21
SPATA41	0.69	7.03258E-55	AC091133.5	0.46	2.44208E-21
FAR2P4	0.69	8.08585E-55	AC092634.8	0.46	2.67843E-21
AC090099.1	0.69	9.56322E-55	TARS3	0.46	2.91424E-21
AC037471.2	0.69	1.85534E-54	AP003119.2	0.46	2.91479E-21
AC245517.4	0.69	2.81637E-54	AC016885.3	0.46	3.178E-21
AL353726.1	0.69	1.29264E-53	PIF1	0.46	3.44358E-21
AC090164.1	0.68	6.48485E-53	AC080080.1	0.46	3.56467E-21
AC092326.1	0.68	3.33285E-52	RAMP2-AS1	0.46	3.69215E-21

MED15P9	0.68	4.8643E-52	AL627309.7	0.46	3.8919E-21
FO681548.1	0.68	6.76902E-52	LINC01948	0.46	3.9701E-21
CCDC144NL	0.68	1.03463E-51	AC019254.1	0.46	4.49442E-21
AC104041.1	0.68	1.14227E-51	AC073488.1	0.46	4.75296E-21
RNU6-1178P	0.67	5.70787E-51	AL133338.1	0.46	4.93486E-21
AC015712.1	0.67	6.55394E-51	SLCO6A1	0.46	4.99945E-21
AC079460.2	0.67	1.69065E-50	CASC6	0.46	5.27662E-21
AC090774.2	0.67	2.08529E-50	FGF14-AS2	0.46	5.71915E-21
AC074389.2	0.67	6.6341E-50	RNU6-695P	0.46	5.89616E-21
KLF2P1	0.67	9.47562E-50	AC136431.1	0.46	5.91963E-21
OR4H6P	0.67	1.70377E-49	SPTY2D1OS	0.46	6.30177E-21
AC025884.1	0.67	2.9208E-49	ZNF192P1	0.46	6.72237E-21
AC025183.2	0.66	7.9419E-49	DNAJC3-DT	0.46	6.88139E-21
AC093326.1	0.66	9.69429E-49	AL132780.5	0.46	7.03138E-21
PHF2P2	0.66	1.8838E-48	DSCR10	0.46	7.87144E-21
AC245517.2	0.66	1.06475E-47	AL118516.1	0.46	8.20111E-21
LINC02348	0.65	3.70032E-47	AL117379.1	0.46	9.72508E-21
HOMER2P1	0.65	3.71309E-47	CYP4F32P	0.46	1.03268E-20
AL031674.1	0.65	4.47326E-47	MAGEA10	0.46	1.05818E-20
MTCO2P32	0.65	7.37471E-47	LINC01311	0.46	1.17568E-20
MED15P8	0.65	1.32015E-46	LINC02393	0.46	1.1958E-20
AC069148.1	0.65	1.43491E-46	AC090164.3	0.46	1.22031E-20
LINC01777	0.65	2.26646E-46	RNU6-924P	0.46	1.30717E-20
AC008805.1	0.65	4.32088E-46	AC099654.5	0.46	1.45314E-20
AL772337.3	0.65	4.89098E-46	AC099487.2	0.45	1.48367E-20
AC010491.1	0.65	7.92077E-46	DUX4L35	0.45	1.50035E-20
AC103996.2	0.65	1.15541E-45	SNX18P14	0.45	1.64217E-20
OR11J2P	0.65	1.29159E-45	MAPK6-DT	0.45	1.66191E-20
AC009477.1	0.64	1.95525E-45	AL356585.4	0.45	1.68134E-20
IGLVV-58	0.64	2.28731E-45	LINC01850	0.45	1.84129E-20
LINC01163	0.64	2.9662E-45	AC069222.1	0.45	1.86789E-20
Z98752.4	0.64	3.09149E-45	AL513320.1	0.45	1.87353E-20
LINC02253	0.64	4.12294E-45	TP53TG3F	0.45	1.87641E-20
AC103808.6	0.64	5.50594E-45	ARHGEF19	0.45	1.95881E-20
ADAM20P1	0.64	7.25696E-45	AC005006.1	0.45	1.97348E-20
FRG1DP	0.64	1.24455E-44	AL158063.1	0.45	2.051E-20
CCDC144NL-AS1	0.64	1.50414E-44	BNIP3P39	0.45	2.43763E-20
NEK2P4	0.64	3.52757E-44	AC138776.1	0.45	2.58816E-20
IGLVI-56	0.64	3.75408E-44	AC011632.1	0.45	2.63836E-20
HERC2P8	0.64	5.10788E-44	RNU6-1055P	0.45	3.01198E-20
LINC02076	0.64	9.91348E-44	AC073464.1	0.45	3.39709E-20

AL096701.4	0.63	1.62362E-43	AC127520.1	0.45	3.67103E-20
USP24P1	0.63	1.65558E-43	CLCN3P1	0.45	4.09614E-20
NF1P2	0.63	2.08908E-43	AC063977.5	0.45	4.12957E-20
OLIG3	0.63	6.97554E-43	AL139005.1	0.45	4.14724E-20
RNASEH1P1	0.63	2.47685E-42	L3MBTL1	0.45	4.52255E-20
FTCD	0.63	3.99191E-42	AC140847.2	0.45	4.53827E-20
AC026412.2	0.62	5.40879E-42	AC231759.2	0.45	4.70031E-20
IGHD5OR15-5B	0.62	6.29669E-42	AC026992.2	0.45	4.85438E-20
OR4N3P	0.62	9.72311E-42	LINC01893	0.45	5.12434E-20
CR381670.1	0.62	1.14349E-41	AP004782.1	0.45	5.60977E-20
CXADRP2	0.62	5.78331E-41	BX276092.7	0.45	5.99111E-20
LINC00653	0.62	5.98582E-41	AL512625.3	0.45	5.99234E-20
GABRG2	0.62	6.87503E-41	RNA5SP519	0.45	6.54878E-20
IGHD2OR15-2A	0.62	2.09497E-40	Z82195.3	0.45	6.72934E-20
TM2D3	0.61	4.00199E-40	AC103794.1	0.45	6.87976E-20
CT45A3	0.61	8.08943E-40	ANKIB1	0.45	6.9512E-20
AL050341.2	0.61	9.17728E-40	AC073578.5	0.45	6.9553E-20
AP000894.4	0.61	9.41291E-40	AL021918.5	0.45	7.06282E-20
ELK2BP	0.61	1.01135E-39	MMEL1-AS1	0.45	7.43127E-20
TP53TG3D	0.61	1.62361E-39	MAGEA9B	0.45	7.70369E-20
LRRD1	0.61	1.91209E-39	AC068790.6	0.45	7.79034E-20
MIR557	0.61	1.92265E-39	AC007193.2	0.45	8.06969E-20
LINC02254	0.61	2.64252E-39	BX649601.1	0.45	8.24932E-20
GRAMD4P8	0.61	2.93383E-39	AC015818.5	0.45	8.49728E-20
AC106052.1	0.60	8.40778E-39	NFYAP1	0.45	9.04339E-20
AC034231.1	0.60	8.48915E-39	LINC00299	0.45	9.22782E-20
AC016575.1	0.60	8.53396E-39	SALL3	0.45	9.46666E-20
TUFMP1	0.60	9.40827E-39	MIR5582	0.45	9.89151E-20
AC090696.1	0.60	1.32306E-38	BMF	0.45	1.10113E-19
AC002351.1	0.60	1.45424E-38	C1GALT1C1L	0.45	1.16494E-19
OR4M2	0.60	1.96785E-38	AP000547.1	0.45	1.17917E-19
AC106799.2	0.60	2.22032E-38	GRM3-AS1	0.45	1.18624E-19
NME8	0.60	2.24303E-38	AC013727.3	0.45	1.20338E-19
AC127381.1	0.60	2.29515E-38	AL158835.2	0.44	1.39562E-19
AC135068.1	0.60	3.10548E-38	AC107926.1	0.44	1.42211E-19
GTF2IP3	0.60	3.44794E-38	CAMTA1-DT	0.44	1.46977E-19
OR11H3P	0.60	3.63339E-38	AC018697.1	0.44	1.53992E-19
LINC01511	0.60	4.98347E-38	AC020704.1	0.44	1.70808E-19
Z95115.1	0.60	5.86466E-38	AC019211.1	0.44	1.75083E-19
AC073578.1	0.60	6.2983E-38	OR56A5	0.44	1.81243E-19
POTEE	0.60	1.40298E-37	SLC25A21-AS1	0.44	1.96152E-19
AC107057.1	0.60	1.8815E-37	CYP51A1P2	0.44	2.05482E-19

RASGEF1C	0.59	2.87793E-37	CYP51A1P1	0.44	2.13403E-19
MRPL45P1	0.59	3.89808E-37	MGAT2P1	0.44	2.21221E-19
AC103808.4	0.59	4.03048E-37	LINC01843	0.44	2.24972E-19
CEP83-DT	0.59	8.15674E-37	AC020550.2	0.44	2.28485E-19
RPS2P32	0.59	1.12486E-36	AC044839.2	0.44	2.39425E-19
AC106799.1	0.59	1.55866E-36	AL606500.1	0.44	2.46683E-19
HOXD-AS2	0.59	2.18369E-36	AL355385.1	0.44	2.59864E-19
LINC01524	0.59	2.58524E-36	H2BW1	0.44	2.60921E-19
AC126407.1	0.59	3.11346E-36	TMBIM7P	0.44	2.67546E-19
AC132872.1	0.59	4.01144E-36	AC091905.1	0.44	2.79157E-19
AC118758.2	0.59	4.28985E-36	OR11Q1P	0.44	2.86858E-19
AP001476.3	0.58	9.672E-36	AL138831.3	0.44	3.09118E-19
AL137001.2	0.58	1.1188E-35	RNA5SP284	0.44	3.12719E-19
SPATA31C2	0.58	1.59727E-35	AL603839.3	0.44	3.18281E-19
IGLVIV-59	0.58	1.88499E-35	ACTBP8	0.44	3.54892E-19
LINC00898	0.58	1.92734E-35	ASB7	0.44	4.05142E-19
GATAD1	0.58	2.34215E-35	AC012625.1	0.44	4.06273E-19
AC116666.1	0.58	3.18482E-35	AC092803.1	0.44	4.29066E-19
AL136162.1	0.58	5.04013E-35	LINC02251	0.44	4.32484E-19
AC106799.3	0.58	7.02789E-35	AC119150.2	0.44	4.50898E-19
NCOR1P2	0.58	8.63803E-35	ANKRD20A8P	0.44	5.80458E-19
AC145350.2	0.58	1.64162E-34	AC242842.1	0.44	6.18181E-19
DUX4L37	0.58	1.64566E-34	LINC02249	0.44	6.58316E-19
LINC00442	0.57	2.47799E-34	SPATA24	0.44	7.11061E-19
AC137761.1	0.57	4.90779E-34	AC239868.1	0.44	7.23255E-19
AC023034.1	0.57	6.16433E-34	AL161669.2	0.44	7.24903E-19
AL356585.1	0.57	6.19296E-34	AC106771.1	0.44	7.73149E-19
AL132655.2	0.57	6.74107E-34	AC006206.2	0.43	9.61784E-19
MYO18B	0.57	6.88558E-34	AC121342.1	0.43	9.66791E-19
AL139254.3	0.57	7.86084E-34	NDUFV2-AS1	0.43	1.01414E-18
DANT1	0.57	8.35316E-34	AP001425.1	0.43	1.06672E-18
MKRN3	0.57	1.0977E-33	AC001226.1	0.43	1.06888E-18
OR7E149P	0.57	1.37351E-33	PEX1	0.43	1.07641E-18
AC124947.2	0.57	1.89717E-33	AL137078.1	0.43	1.13597E-18
CDH8	0.57	2.11929E-33	AC126915.2	0.43	1.15195E-18
AC000120.1	0.57	2.23515E-33	LVRN	0.43	1.16706E-18
AC103808.3	0.57	4.43141E-33	LINC01233	0.43	1.1939E-18
AC068473.5	0.57	4.58606E-33	AL049557.1	0.43	1.19665E-18
AC025811.1	0.57	4.7811E-33	GUCY2D	0.43	1.20308E-18
AC073869.7	0.56	6.6917E-33	CTAG2	0.43	1.24829E-18
H2BU2P	0.56	9.03993E-33	ARID3A	0.43	1.27321E-18
AC023024.1	0.56	9.26964E-33	RARRES2P6	0.43	1.37678E-18

KRIT1	0.56	1.07001E-32	PRSS44P	0.43	1.38213E-18
RNA5SP359	0.56	2.09245E-32	IRX4-AS1	0.43	1.43272E-18
VPS50	0.56	3.0505E-32	AC026801.2	0.43	1.43401E-18
AC098656.1	0.56	3.08674E-32	AC107464.2	0.43	1.46032E-18
ZNF519P3	0.56	3.53138E-32	GABPB1-IT1	0.43	1.49238E-18
LINC00652	0.56	4.01066E-32	AC099654.4	0.43	1.52868E-18
AC183088.1	0.56	4.29819E-32	AC005156.1	0.43	1.5489E-18
AC060814.2	0.56	8.93401E-32	AL356475.1	0.43	1.69414E-18
CECR2	0.56	1.0428E-31	AC025031.4	0.43	1.69716E-18
SELENOS	0.56	1.04338E-31	AP001468.1	0.43	1.81539E-18
AC009271.2	0.55	1.26843E-31	AC009812.4	0.43	1.8242E-18
LINC02145	0.55	1.67504E-31	GTF2IP6	0.43	1.8438E-18
FAM169B	0.55	2.08073E-31	SAA3P	0.43	1.95913E-18
AC134684.11	0.55	2.13338E-31	C11orf71	0.43	1.99233E-18
AL137802.2	0.55	2.59514E-31	AL358777.3	0.43	2.04654E-18
POTEF	0.55	3.11771E-31	MIR5087	0.43	2.09532E-18
DSCR4-IT1	0.55	4.04868E-31	CARS1P2	0.43	2.13328E-18
DSCR4	0.55	5.39548E-31	MTND6P29	0.43	2.17315E-18
CST9LP2	0.55	1.08694E-30	AC016710.1	0.43	2.27449E-18
AC008443.2	0.55	1.17381E-30	TCF15	0.43	2.47575E-18
DBX1	0.55	1.19132E-30	MIR2052HG	0.43	2.47984E-18
AL031736.2	0.55	1.42178E-30	RN7SKP106	0.43	2.53538E-18
AL137100.3	0.55	1.60728E-30	AC024361.1	0.43	2.56126E-18
AL355602.1	0.55	2.01202E-30	AP005262.1	0.43	3.14654E-18
LINC01001	0.54	2.54604E-30	LINC02456	0.43	3.5314E-18
CR786580.1	0.54	3.34463E-30	AL391152.1	0.43	3.61491E-18
STK24-AS1	0.54	3.41888E-30	C2orf16	0.43	3.70929E-18
RNA5SP70	0.54	4.74951E-30	NINL	0.43	3.80994E-18
AC104667.1	0.54	5.77934E-30	C2orf68	0.43	3.98878E-18
AC012378.2	0.54	6.46451E-30	AC007092.1	0.43	4.3437E-18
YPEL5P1	0.54	8.31874E-30	AC068205.2	0.43	4.34611E-18
LINC02207	0.54	9.50111E-30	FRG1GP	0.43	4.72617E-18
LINC01056	0.54	1.01158E-29	LINC01446	0.43	4.84807E-18
CT45A1	0.54	1.13394E-29	AL590822.3	0.43	5.29645E-18
AC097382.3	0.54	1.86479E-29	MIR718	0.43	5.77886E-18
LUNAR1	0.54	1.87384E-29	AC004597.1	0.43	6.26806E-18
CR381670.2	0.54	1.95399E-29	ALOX12P2	0.43	6.33378E-18
NXF2	0.54	2.04924E-29	NLRP3P1	0.43	6.72284E-18
AF186192.1	0.54	2.15047E-29	LINC02163	0.42	7.32405E-18
AL137001.1	0.54	2.16177E-29	MIR548AR	0.42	7.64309E-18
AC073869.3	0.54	3.02519E-29	SYCP2L	0.42	7.70302E-18
AC113349.1	0.54	3.3008E-29	TTC28-AS1	0.42	7.77264E-18

CTAG1B	0.54	3.64158E-29	UBAC2-AS1	0.42	7.96067E-18
H2AZ1-DT	0.53	4.20851E-29	LINC00540	0.42	9.49985E-18
AC010608.2	0.53	4.88269E-29	AC022031.2	0.42	9.56736E-18
MRPS33P4	0.53	5.9217E-29	ZNRD2-AS1	0.42	9.75915E-18
CYP4F27P	0.53	6.8744E-29	ARRDC4	0.42	1.09163E-17
AC026341.3	0.53	8.80084E-29	FP325330.3	0.42	1.11459E-17
AC091271.1	0.53	9.03198E-29	BRCA1P1	0.42	1.14951E-17
FAM30B	0.53	1.03812E-28	AC023141.2	0.42	1.21055E-17
MRPS31P2	0.53	1.26033E-28	AC012640.2	0.42	1.23122E-17
AL589794.2	0.53	1.35784E-28	TTC34	0.42	1.28241E-17
AC139491.3	0.53	1.43979E-28	AL355297.3	0.42	1.33929E-17
LINC00648	0.53	1.60573E-28	ASNSP1	0.42	1.43747E-17
RBM48	0.53	1.7367E-28	AC132192.2	0.42	1.56405E-17
AC068544.2	0.53	2.08018E-28	RAB6C-AS1	0.42	1.6122E-17
AC063948.1	0.53	2.36297E-28	LINC01666	0.42	1.61886E-17
VN1R54P	0.53	3.22909E-28	F11-AS1	0.42	1.62247E-17
AC099489.1	0.53	3.73987E-28	CYCSP41	0.42	1.66359E-17
BCL11A	0.53	4.00793E-28	AC018641.1	0.42	1.70927E-17
AL590787.1	0.53	5.39212E-28	AP003108.5	0.42	1.72805E-17
LINC01756	0.53	5.45766E-28	AP000873.2	0.42	1.77118E-17
AL078622.1	0.52	8.74561E-28	POTEH	0.42	1.81745E-17
KIF25-AS1	0.52	9.10577E-28	LINC01807	0.42	1.83723E-17
MIR1285-1	0.52	9.48333E-28	C17orf100	0.42	1.96896E-17
AC026495.1	0.52	9.89194E-28	H2BC19P	0.42	1.97748E-17
AL117329.1	0.52	1.01991E-27	AC009271.1	0.42	2.00626E-17
AC005252.3	0.52	1.02357E-27	PRR3	0.42	2.00981E-17
TOLLIP-AS1	0.52	1.07927E-27	SUGT1P4-STRA6LP	0.42	2.02924E-17
AC090985.1	0.52	1.33286E-27	AC092427.1	0.42	2.16307E-17
SNRPA1	0.52	1.51857E-27	AC109347.1	0.42	2.24633E-17
ABHD17AP4	0.52	1.52442E-27	AC104794.4	0.42	2.25472E-17
AC116565.1	0.52	1.8304E-27	AC026992.1	0.42	2.27803E-17
NOTO	0.52	1.91657E-27	AC099791.2	0.42	2.36138E-17
AC090907.1	0.52	2.28102E-27	RGL2	0.42	2.40681E-17
AL136221.1	0.52	2.29991E-27	AL445531.1	0.42	2.48953E-17
AC010275.1	0.52	2.36475E-27	LINC00052	0.42	2.5798E-17
AL353801.3	0.52	2.38164E-27	HLA-DRB9	0.42	2.73625E-17
AC100812.1	0.52	2.39248E-27	AC092685.1	0.42	2.79002E-17
CHCHD2P7	0.52	2.46998E-27	AC090970.2	0.42	2.91348E-17
AC026108.1	0.52	2.6338E-27	AL033530.1	0.42	3.02681E-17
AL139384.1	0.52	2.67128E-27	TOB1-AS1	0.42	3.06571E-17
CLCP1	0.52	3.44245E-27	PACSIN3	0.42	3.16604E-17

AC112176.1	0.52	3.58394E-27	CU633967.1	0.42	3.20201E-17
LINC00865	0.52	3.58479E-27	LINC01448	0.42	3.33121E-17
SNCAIP	0.52	3.64756E-27	DPP4-DT	0.42	3.38539E-17
AL732372.2	0.52	3.8093E-27	LINC00491	0.42	3.45215E-17
AC090774.1	0.52	3.85324E-27	HOXD9	0.42	3.54323E-17
ZBED9	0.52	4.05206E-27	AP005119.2	0.42	3.54879E-17
BMS1P22	0.52	4.54695E-27	AC092171.5	0.42	3.71402E-17
RNU6-705P	0.52	5.19085E-27	LINS1	0.42	3.80355E-17
AC079160.1	0.52	6.93738E-27	AC126177.6	0.42	3.95169E-17
GNAS-AS1	0.52	6.97821E-27	AC127455.1	0.42	4.26659E-17
MIR653	0.52	7.27084E-27	PCAT19	0.42	4.29154E-17
AC108002.2	0.52	7.38845E-27	AC036111.1	0.42	4.39798E-17
AC091133.4	0.52	8.15716E-27	ARIH2OS	0.42	4.46468E-17
AC097532.2	0.52	8.60117E-27	AL034550.3	0.42	4.56177E-17
CAHM	0.51	9.23334E-27	AL035427.1	0.42	4.56909E-17
ADAMTS17	0.51	1.03842E-26	TSPEAR-AS1	0.42	4.6601E-17
AC090907.3	0.51	1.05338E-26	AL022100.1	0.42	4.79999E-17
ROBO3	0.51	1.11024E-26	AC245100.7	0.41	4.98809E-17
AC092634.3	0.51	1.30137E-26	TCTE3	0.41	4.99848E-17
KARS1P2	0.51	1.38412E-26	AC092447.7	0.41	5.24057E-17
AP001781.1	0.51	1.51833E-26	NUS1P2	0.41	5.43084E-17
KCNMB3P1	0.51	1.83475E-26	AL357556.3	0.41	5.44255E-17
LINC01456	0.51	2.04156E-26	GTSE1-DT	0.41	5.5546E-17
PRAME	0.51	2.15051E-26	FRAS1	0.41	5.86708E-17
HERC2P4	0.51	2.73612E-26	RSL24D1P3	0.41	5.97704E-17
AL359513.1	0.51	3.9253E-26	AL023755.1	0.41	5.99459E-17
AC011029.1	0.51	4.24635E-26	AC129507.3	0.41	6.00149E-17
AC125494.3	0.51	4.74413E-26	MIR1305	0.41	6.42455E-17
LINC02696	0.51	5.32167E-26	LINC01639	0.41	6.54411E-17
LINC01146	0.51	5.99206E-26	ESRRAP2	0.41	6.54548E-17
KIAA0895LP1	0.51	6.30211E-26	AC016590.3	0.41	6.75487E-17
AL691520.1	0.51	7.28807E-26	AC010719.1	0.41	7.34395E-17
NAALAD2	0.51	7.32526E-26	AC026412.3	0.41	7.59267E-17
AC136475.2	0.51	1.07174E-25	C5orf34-AS1	0.41	7.93505E-17
AC090527.3	0.50	1.32105E-25	CASC20	0.41	8.30224E-17
AC060780.2	0.50	1.42779E-25	SULT1C4	0.41	8.32533E-17
SLC25A3P3	0.50	1.45396E-25	SSBL4P	0.41	8.39358E-17
MIR647	0.50	1.5159E-25	AC007383.1	0.41	1.04138E-16
H2AZ2P1	0.50	1.6815E-25	AC099565.1	0.41	1.0754E-16
AC068831.5	0.50	2.69026E-25	SLC47A1P2	0.41	1.08346E-16
CNTN5	0.50	2.88314E-25	AL358780.1	0.41	1.10876E-16
AC016355.1	0.50	3.10192E-25	AC119751.4	0.41	1.13696E-16

DSCR8	0.50	3.21923E-25	AC069061.2	0.41	1.20485E-16
AL645929.1	0.50	3.32181E-25	AC095057.3	0.41	1.31096E-16
AC005391.1	0.50	3.35162E-25	AL023802.1	0.41	1.31778E-16
AC022762.1	0.50	3.92163E-25	AVP	0.41	1.32487E-16
LRRC28	0.50	4.17447E-25	TSKS	0.41	1.34364E-16
PASD1	0.50	4.24969E-25	RNA5SP532	0.41	1.34427E-16
PCDHB5	0.50	4.77229E-25	IGF2BP1	0.41	1.37118E-16
RN7SL832P	0.50	5.13957E-25	PXN-AS1	0.41	1.39419E-16
PLAG1	0.50	6.164E-25	POM121L2	0.41	1.44059E-16
AC135068.2	0.50	6.2877E-25	OBSCN-AS1	0.41	1.4563E-16
MRPL46	0.50	7.4346E-25	LINC01793	0.41	1.54603E-16
PARTICL	0.50	7.70311E-25	USP32P3	0.41	1.56911E-16
AC109583.1	0.50	9.11713E-25	RNVU1-26	0.41	1.58117E-16
AL139042.1	0.50	9.31676E-25	AL132765.2	0.41	1.70509E-16
AP000640.1	0.50	1.00221E-24	AC097374.1	0.41	1.79832E-16
AC073488.6	0.50	1.05898E-24	AL137077.2	0.41	1.83725E-16
AC139491.1	0.50	1.11634E-24	MRPL45P2	0.41	1.88085E-16
AC091925.1	0.50	1.13895E-24	AC097639.1	0.41	1.89416E-16
AC017083.1	0.50	1.27789E-24	AC092745.4	0.41	2.10995E-16
AC073578.2	0.50	1.29602E-24	MKS1	0.41	2.25634E-16
AC000032.1	0.49	1.44654E-24	LMCD1-AS1	0.41	2.36244E-16
AC118658.1	0.49	1.67631E-24	SNORD115-32	0.41	2.48457E-16
EIF2AK3-DT	0.49	1.68017E-24	HOXD1	0.41	2.54934E-16
COA6-AS1	0.49	1.68924E-24	AC027584.1	0.41	2.63271E-16
DUX4L27	0.49	1.99458E-24	AP005057.1	0.41	2.75529E-16
AC009084.2	0.49	2.29218E-24	LINC00508	0.41	2.86628E-16
AC010999.2	0.49	2.30777E-24	AP005119.1	0.41	3.01384E-16
AC008443.5	0.49	2.33213E-24	AC096633.1	0.40	3.59475E-16
AL844170.1	0.49	2.38733E-24	AL596442.3	0.40	3.60224E-16
AL356596.1	0.49	2.55071E-24	PAN3-AS1	0.40	3.77228E-16
AC023024.2	0.49	2.85737E-24	AC007881.3	0.40	3.82249E-16
LINC02550	0.49	3.02149E-24	HNRNPA1P62	0.40	3.94534E-16
OXCT2	0.49	3.23859E-24	AC002056.2	0.40	3.96214E-16
NKAIN1P2	0.49	3.25021E-24	AL590326.1	0.40	3.9726E-16
FAM133B	0.49	4.88211E-24	PRR23D3P	0.40	4.06493E-16
AC114956.1	0.49	5.44824E-24	AC011370.1	0.40	4.23327E-16
AC062029.1	0.49	5.65318E-24	HOXD13	0.40	4.30923E-16
PANK2-AS1	0.49	6.53301E-24	AC003973.2	0.40	4.37886E-16
AP001476.1	0.49	7.16568E-24	RN7SKP184	0.40	4.46502E-16
CCDC39-AS1	0.49	7.18203E-24	AMZ2P3	0.40	4.70798E-16
MRGPRX13P	0.49	7.47592E-24	C8orf44	0.40	4.95919E-16
IGHD1OR15-1A	0.49	7.6533E-24	AL132780.2	0.40	4.98805E-16

ABHD17AP5	0.49	7.92705E-24	TULP1	0.40	5.01468E-16
FAM41C	0.49	8.2899E-24	AL356123.2	0.40	5.03084E-16
AC099684.2	0.49	1.0296E-23	MTND4P4	0.40	5.16277E-16
AC245517.3	0.49	1.05734E-23	NRBF2P2	0.40	5.36853E-16
HSPA8P13	0.49	1.08219E-23	AL121658.1	0.40	5.53013E-16
AC016825.1	0.49	1.25938E-23	UCKL1	0.40	5.63876E-16
AL390198.1	0.49	1.4316E-23	AC108727.1	0.40	5.71171E-16
AC022819.1	0.49	1.47369E-23	IQCG	0.40	5.82199E-16
LINC02610	0.49	1.52698E-23	C17orf113	0.40	5.98153E-16
AC037479.1	0.48	1.84202E-23	AL023806.1	0.40	6.13024E-16
HOXD10	0.48	1.99421E-23	SEMA6A-AS1	0.40	6.21252E-16
SLC25A15P4	0.48	2.72693E-23	CYP51A1	0.40	6.32162E-16
AL080284.1	0.48	3.16024E-23	NEU1	0.40	6.38871E-16
LINC01977	0.48	3.36136E-23	AL133243.2	0.40	6.68283E-16
ITPR1-DT	0.48	3.64594E-23	AC126177.5	0.40	6.79099E-16
AC145350.3	0.48	3.69945E-23	AL008636.1	0.40	6.81278E-16
KLF14	0.48	3.72439E-23	GPR176-DT	0.40	6.90954E-16
F10-AS1	0.48	3.76119E-23	MTCYBP29	0.40	6.91529E-16
AC108488.3	0.48	4.06718E-23	LINC00470	0.40	7.02367E-16
AC092335.1	0.48	4.15732E-23	LINC01415	0.40	7.06091E-16
AC008124.1	0.48	4.25219E-23	AC069544.1	0.40	7.06281E-16
AC015961.2	0.48	4.67501E-23	AL158070.2	0.40	7.52985E-16
AL442067.3	0.48	5.40062E-23	AC090589.3	0.40	7.89382E-16
NOC2LP1	0.48	5.5552E-23	AC009955.4	0.40	7.9067E-16
AL513550.1	0.48	5.60518E-23	AL021707.2	0.40	7.94752E-16
LINC02315	0.48	6.35585E-23	ISX	0.40	8.05589E-16
ATP1A1-AS1	0.48	6.49526E-23	ASXL1	0.40	8.34074E-16
ZNF334	0.48	7.02134E-23	AC073389.3	0.40	8.34783E-16
FAM230C	0.48	7.21929E-23	AL713922.1	0.40	8.48395E-16
AC119403.1	0.48	7.92969E-23	OFCC1	0.40	9.76771E-16
HERC2P5	0.48	8.0436E-23	AL133153.1	0.40	1.05731E-15
AC026671.1	0.48	8.81674E-23	VN1R71P	0.40	1.09336E-15
AFDN-DT	0.48	8.91016E-23	FAR2P3	0.40	1.09748E-15
MIR4453HG	0.48	9.30321E-23	LINC00482	0.40	1.11314E-15
AC007566.1	0.48	9.38181E-23	C5orf58	0.40	1.1415E-15
SYN3	0.48	9.82099E-23	AC092910.3	0.40	1.18209E-15
AL132655.1	0.48	1.00455E-22	NIFK-AS1	0.40	1.1961E-15
ZNF560	0.48	1.00563E-22	AL161669.3	0.40	1.24573E-15
AACSP1	0.48	1.02921E-22	AC006206.1	0.40	1.24746E-15
AC132807.2	0.48	1.03799E-22	AP000829.1	0.40	1.26474E-15
SLC6A13	0.48	1.11228E-22	LINC01592	0.40	1.26817E-15
CALN1	0.48	1.312E-22	ZNF519	0.40	1.30209E-15

AC093627.5	0.48	1.3307E-22	AC090517.2	0.40	1.33514E-15
HMGN2P15	0.48	1.48605E-22	LINC01143	0.40	1.35777E-15
AC021087.3	0.48	1.54522E-22	DNMT3B	0.40	1.3625E-15
AC090826.1	0.48	1.59518E-22	AC010894.1	0.40	1.38639E-15
EXOC3-AS1	0.48	1.66187E-22	GUSBP10	0.40	1.41835E-15
SALL4	0.47	1.87902E-22	AL355596.1	0.40	1.44407E-15
CTAG1A	0.47	1.9066E-22	LINC01424	0.40	1.44803E-15
AC140479.3	0.47	1.91627E-22	FRG1FP	0.40	1.53329E-15
ZNF280A	0.47	2.02049E-22	MBL1P	0.40	1.54085E-15
AC135068.8	0.47	2.06172E-22	AC237221.2	0.40	1.54714E-15
AC023824.3	0.47	2.08943E-22	AC233702.1	0.40	1.65269E-15
AC129926.2	0.47	2.17469E-22	AGGF1P10	0.40	1.67012E-15
AL357833.1	0.47	2.30575E-22	AC239859.5	0.40	1.69189E-15
AC084855.2	0.47	2.30899E-22	ZNF682	0.40	1.70222E-15
			FOXN4	0.40	1.74826E-15

Table S6. Genes significantly correlated with LINC01087 expression in testicular germ cell tumor carcinoma (TCGA).

Gene symbol	Positive R value	P-value	Gene symbol	Positive R value	P-value
POTEKP	0.98	8.011E-106	CYP4F27P	0.47	7.00781E-10
AC093838.1	0.89	3.03876E-55	DEFB108B	0.47	7.10577E-10
MED15P4	0.88	2.52966E-52	AC008080.2	0.47	7.1239E-10
LINC01518	0.87	3.84773E-50	LYPD9P	0.47	7.26741E-10
FAR2P4	0.86	3.92245E-46	LINC02470	0.47	7.36368E-10
NEK2P4	0.81	9.36716E-38	TSPAN18-AS1	0.47	8.28077E-10
KLF2P4	0.81	1.18027E-37	MROH2A	0.47	8.50081E-10
GRAMD4P8	0.80	5.04393E-36	AC063977.4	0.47	8.74424E-10
NF1P8	0.78	1.75758E-33	AP002008.2	0.47	9.01823E-10
CR769776.3	0.78	9.33232E-33	AC083906.4	0.47	9.20414E-10
KLF2P1	0.77	2.21707E-32	ABTB2	0.47	9.28077E-10
POTEF	0.74	8.56954E-28	AP001992.1	0.46	9.62062E-10
AC245517.2	0.73	2.43827E-27	AC023468.1	0.46	9.99099E-10
NF1P7	0.73	3.6848E-27	AC007846.1	0.46	1.04852E-09
SNORD56B	0.73	3.98763E-27	LINC02527	0.46	1.05081E-09
AC135068.1	0.73	4.77114E-27	RNA5SP65	0.46	1.15159E-09
ANKRD20A1	0.72	1.64792E-26	AC022400.8	0.46	1.18377E-09
NEK2P1	0.72	1.84012E-26	AC025539.1	0.46	1.27643E-09
AL512310.11	0.72	2.47755E-26	CTAG1A	0.46	1.33471E-09
CTCFL	0.72	3.78984E-26	VCX3B	0.46	1.34733E-09
AC018865.2	0.72	5.01659E-26	AC114401.1	0.46	1.39734E-09
NCOR1P2	0.72	8.40193E-26	CTAG2	0.46	1.43191E-09
AC073869.3	0.71	2.64017E-25	AC114485.1	0.46	1.52085E-09
AC245517.4	0.71	6.97186E-25	PRSS43P	0.46	1.54414E-09
RNU6-508P	0.70	1.12728E-24	MED15P1	0.46	1.56473E-09
RNA5SP133	0.70	1.12728E-24	SLC25A18	0.46	1.57752E-09
RNU6-115P	0.70	1.12728E-24	LINC02022	0.46	1.67739E-09
RNU6-741P	0.70	1.12728E-24	BX664727.3	0.46	1.68903E-09
BX088651.1	0.70	1.12728E-24	AC016903.2	0.46	1.73231E-09
RNU6ATAC41P	0.70	1.12728E-24	AC136431.1	0.46	2.03601E-09
RN7SKP59	0.70	1.12728E-24	TPM3P9	0.46	2.05863E-09
FAM197Y5	0.70	1.12728E-24	AC244102.1	0.46	2.21691E-09
AL035417.1	0.70	1.12728E-24	AP002449.1	0.46	2.40564E-09
AC007967.2	0.70	1.12728E-24	TCHH	0.45	2.44196E-09
AL135790.2	0.70	1.12728E-24	AL080276.1	0.45	2.49868E-09
MTND6P14	0.70	1.12728E-24	CPB2	0.45	2.6008E-09
AL354766.1	0.70	1.12728E-24	AL132655.1	0.45	2.62247E-09
RPL30P5	0.70	1.12728E-24	AP005210.2	0.45	2.69372E-09

AC113347.2	0.70	1.12728E-24	AC007114.2	0.45	2.73821E-09
AC010625.1	0.70	1.12728E-24	MIR6779	0.45	3.01021E-09
OR11H3P	0.70	1.12728E-24	AC003093.1	0.45	3.05425E-09
MIR3199-1	0.70	1.12728E-24	ZNF705E	0.45	3.07008E-09
AC010655.4	0.70	1.12728E-24	RNA5SP131	0.45	3.14997E-09
RN7SL719P	0.70	1.12728E-24	AL391380.1	0.45	3.32723E-09
IGHD2OR15-2B	0.70	1.12728E-24	RAB6D	0.45	3.39262E-09
AC140658.3	0.70	3.05794E-24	AC121247.2	0.45	3.40768E-09
POTEI	0.69	2.57725E-23	MRGPRX8P	0.45	3.40926E-09
VN1R54P	0.68	7.45637E-23	GAB2	0.45	3.44726E-09
AC131280.1	0.68	8.60713E-23	AC104009.1	0.45	3.57582E-09
ANKRD20A3P	0.68	1.50593E-22	LINC01432	0.45	3.57689E-09
NF1P6	0.68	2.2286E-22	MIR3164	0.45	3.63077E-09
AC023310.4	0.68	2.72791E-22	ATP6V1B1	0.45	3.64006E-09
AC009477.1	0.68	3.21873E-22	AL355377.4	0.45	3.70641E-09
FAR2P1	0.68	4.32718E-22	AF099810.1	0.45	3.74163E-09
LINC02203	0.67	1.78447E-21	RN7SKP241	0.45	3.88304E-09
IGHV1-14	0.67	2.01231E-21	MTCO1P48	0.45	3.98281E-09
AC037471.1	0.65	1.87027E-20	AC007064.2	0.45	4.07884E-09
POTEE	0.65	2.28188E-20	RPS17P14	0.45	4.16846E-09
IGKV2-28	0.65	4.90488E-20	E2F3-IT1	0.45	4.18381E-09
AC135068.2	0.65	6.20793E-20	MTCP1	0.45	4.26421E-09
POTEH	0.64	1.34161E-19	AC004453.2	0.45	4.57567E-09
AC135068.11	0.64	2.05878E-19	LINC01804	0.45	4.80121E-09
AC140481.3	0.64	2.20318E-19	SMCR5	0.45	4.91214E-09
ART1	0.64	3.90504E-19	OR2L2	0.45	4.96816E-09
RNU6-71P	0.64	5.01809E-19	GAL3ST1	0.45	5.12443E-09
ABBA01006766.1	0.63	8.39729E-19	PRSS38	0.45	5.16086E-09
AC100757.2	0.63	1.53603E-18	GTF2IP3	0.45	5.38714E-09
AC183088.2	0.63	1.55752E-18	ZNF280A	0.45	5.49502E-09
RPSAP58	0.63	1.56319E-18	AC137695.2	0.45	5.60929E-09
AC127381.1	0.63	2.72803E-18	AC027313.1	0.45	5.62987E-09
GPAT2P1	0.62	4.43589E-18	AL627223.1	0.45	5.69997E-09
AL929601.1	0.62	6.71884E-18	PGBP	0.45	5.81942E-09
UBBP4	0.62	8.18432E-18	KRTAP6-1	0.45	5.83532E-09
IGHJ5	0.62	8.52603E-18	RPS15P9	0.44	5.88823E-09
PCP4L1	0.61	2.33987E-17	AC093110.1	0.44	5.98625E-09
TMEM132D-AS1	0.61	2.62555E-17	AC002525.1	0.44	6.32284E-09
NBEAP1	0.60	7.76719E-17	GRAMD4P3	0.44	6.63112E-09
NHP2P1	0.60	1.39182E-16	LINC02646	0.44	6.78084E-09
RCVRN	0.60	1.42076E-16	ELF2P4	0.44	6.80746E-09
AC074389.2	0.60	2.02359E-16	BTBD10	0.44	6.87693E-09

NF1P4	0.59	3.78413E-16	AL023773.1	0.44	6.97912E-09
THUMPD3P1	0.59	5.47711E-16	AC006972.1	0.44	7.33173E-09
KRTAP19-8	0.59	7.03208E-16	AC027419.1	0.44	7.58908E-09
AC005521.1	0.59	7.43929E-16	AL451142.1	0.44	7.67731E-09
IGHVII-43-1	0.58	1.13868E-15	RAC3	0.44	7.76981E-09
NF1P10	0.58	1.15355E-15	AC110015.1	0.44	7.87069E-09
AL157886.1	0.58	1.16882E-15	RNU6-1048P	0.44	8.50763E-09
AL353726.1	0.58	1.35171E-15	AC010145.1	0.44	9.33009E-09
RN7SKP204	0.58	1.38975E-15	MED15P9	0.44	9.9481E-09
AC007923.1	0.58	1.5461E-15	MIR4433B	0.44	1.0498E-08
MED15P8	0.58	1.54797E-15	DQX1	0.44	1.06731E-08
AL033523.2	0.58	1.62385E-15	TUBAP7	0.44	1.08638E-08
AC097374.1	0.58	1.79092E-15	AC087393.1	0.44	1.09976E-08
EDDM3A	0.58	2.22172E-15	LINC01193	0.44	1.15334E-08
AC079466.1	0.58	2.29111E-15	ORM2	0.44	1.17819E-08
CGREF1	0.58	2.86023E-15	FAM9A	0.44	1.18622E-08
AL356489.4	0.58	4.12501E-15	TTC21B-AS1	0.44	1.19473E-08
AL139327.1	0.57	4.3419E-15	Z82185.1	0.44	1.19662E-08
AL445985.2	0.57	4.6646E-15	GGT3P	0.44	1.2831E-08
MAGEA1	0.57	5.70977E-15	AL139095.2	0.44	1.29052E-08
SSXP1	0.57	5.7326E-15	PABPC1P10	0.44	1.30718E-08
RNU6-873P	0.57	5.7326E-15	GABRA6	0.44	1.34587E-08
RN7SKP93	0.57	5.7326E-15	AC121161.2	0.43	1.39961E-08
RNA5SP516	0.57	5.7326E-15	AL512329.1	0.43	1.41644E-08
MIR1238	0.57	5.7326E-15	USP17L2	0.43	1.45413E-08
CDKN2AIPNLP3	0.57	5.7326E-15	RN7SL812P	0.43	1.46257E-08
NDUFA12P1	0.57	5.7326E-15	AC003686.1	0.43	1.4855E-08
Z98751.1	0.57	5.7326E-15	MIR4740	0.43	1.54195E-08
ZNF886P	0.57	5.7326E-15	OR56A5	0.43	1.54955E-08
TPMTP3	0.57	5.7326E-15	AC068058.1	0.43	1.65384E-08
BX284632.1	0.57	5.7326E-15	RPL3P8	0.43	1.74703E-08
RN7SL654P	0.57	5.7326E-15	AC083806.3	0.43	1.78355E-08
AC012087.2	0.57	5.7326E-15	ELOCP27	0.43	1.80531E-08
AC241929.1	0.57	5.7326E-15	STAM-AS1	0.43	1.83037E-08
AL590410.1	0.57	5.7326E-15	RPS6P2	0.43	1.85083E-08
VCX3A	0.57	5.97833E-15	RPS3AP13	0.43	1.88636E-08
AC138035.2	0.57	6.21263E-15	RN7SL523P	0.43	1.89996E-08
AC139365.2	0.57	9.38106E-15	AF064863.1	0.43	1.90657E-08
LINC02780	0.57	1.02428E-14	MAGEA6	0.43	1.91278E-08
CENPIP1	0.57	1.17023E-14	AL109933.3	0.43	1.96991E-08
PNMA6E	0.57	1.23392E-14	RAP1AP	0.43	1.97856E-08
AC006927.5	0.57	1.27993E-14	ACVR2B	0.43	1.98147E-08

VCX2	0.57	1.34191E-14	RNU6-1143P	0.43	1.98309E-08
FP325317.2	0.57	1.43499E-14	AC108865.1	0.43	1.9934E-08
AC244107.1	0.57	1.45229E-14	AL121949.3	0.43	2.07417E-08
AC132938.6	0.57	1.51749E-14	AL132775.2	0.43	2.07983E-08
BX664718.1	0.56	1.59868E-14	LINC00244	0.43	2.08321E-08
LINC01029	0.56	1.7296E-14	AL929236.1	0.43	2.10327E-08
OMP	0.56	1.97292E-14	ANKRD36	0.43	2.11599E-08
AC107886.1	0.56	1.98717E-14	ARPP19P1	0.43	2.1624E-08
LINC00454	0.56	2.57957E-14	LINC01587	0.43	2.17281E-08
FO082842.1	0.56	4.14976E-14	ZNF761	0.43	2.175E-08
MAGEA9B	0.56	4.62765E-14	AL159987.2	0.43	2.1824E-08
AC100757.1	0.56	4.62827E-14	AL110505.1	0.43	2.19526E-08
SNX18P9	0.56	4.81782E-14	AL359198.1	0.43	2.25291E-08
LINC01639	0.55	7.42967E-14	AL121894.3	0.43	2.27436E-08
NF1P9	0.55	7.96523E-14	MIR4632	0.43	2.31714E-08
OR7E16P	0.55	8.17789E-14	NDUFS5P2	0.43	2.32873E-08
AL121949.2	0.55	8.30323E-14	AC023090.2	0.43	2.36316E-08
OR52E3P	0.55	9.58919E-14	NF1P1	0.43	2.39318E-08
HM13-AS1	0.55	1.04152E-13	PRSS40B	0.43	2.41051E-08
AC116666.1	0.55	1.20455E-13	PCAT7	0.43	2.4416E-08
TPM3P6	0.55	1.29967E-13	ABHD17AP4	0.43	2.45501E-08
GPAT2	0.55	1.3979E-13	TPT1P3	0.43	2.61385E-08
RN7SKP209	0.55	1.5141E-13	AC018358.1	0.43	2.61711E-08
AC092490.1	0.55	1.60729E-13	IGLVV-58	0.43	2.62069E-08
AC008543.2	0.55	1.68139E-13	TMEM147-AS1	0.43	2.76716E-08
OR7E4P	0.55	1.68849E-13	CYP4X1	0.43	2.82803E-08
GPR55	0.54	1.93361E-13	AC025031.4	0.43	3.01838E-08
PHF2P2	0.54	2.06319E-13	AC073862.3	0.43	3.07314E-08
LINC01251	0.54	2.21298E-13	NOC2LP2	0.43	3.14451E-08
GPR151	0.54	2.25819E-13	DENND2C	0.42	3.20329E-08
AC011997.2	0.54	2.59748E-13	ANKRD20A7P	0.42	3.26122E-08
AP000244.1	0.54	2.71509E-13	DEFB108C	0.42	3.28577E-08
AC068790.3	0.54	2.7895E-13	AL139109.1	0.42	3.2962E-08
AC068790.5	0.54	2.81542E-13	AC005520.1	0.42	3.36076E-08
USP24P1	0.54	3.90859E-13	LINC00470	0.42	3.51665E-08
RNU6-1306P	0.54	4.13333E-13	NBPF17P	0.42	3.55508E-08
AL513478.4	0.54	4.5442E-13	SYNDIG1L	0.42	3.56249E-08
ELK2BP	0.54	5.9921E-13	RNU6-1283P	0.42	3.57256E-08
AC005006.1	0.53	6.55492E-13	AC239859.1	0.42	3.64605E-08
SLC5A11	0.53	6.8714E-13	AC009102.4	0.42	3.75171E-08
VCX	0.53	7.40679E-13	BCRP7	0.42	3.83201E-08

AL021937.3	0.53	7.57565E-13	EDDM3B	0.42	3.93336E-08
AC114752.2	0.53	7.82049E-13	CLDN18	0.42	4.30394E-08
CCDC144NL	0.53	9.41655E-13	KPNB1P1	0.42	4.57454E-08
AC066580.1	0.53	9.41758E-13	RGPD5	0.42	4.68686E-08
MED15P6	0.53	9.84272E-13	LINC02365	0.42	4.75159E-08
AC016687.3	0.53	1.15873E-12	CSAG1	0.42	4.78361E-08
AC005291.1	0.53	1.20562E-12	TPTE2P1	0.42	4.85793E-08
ANKRD18DP	0.53	1.21692E-12	AC048337.1	0.42	4.91992E-08
AC092623.1	0.53	1.25586E-12	MOBP	0.42	4.92016E-08
DPRXP1	0.53	1.53958E-12	AL929601.2	0.42	4.92963E-08
NUTF2P4	0.53	1.59339E-12	AL049765.2	0.42	5.04123E-08
MAGEA10	0.53	1.66413E-12	AC131180.1	0.42	5.06147E-08
LINC02675	0.53	1.71517E-12	AP005205.3	0.42	5.10978E-08
AC092803.2	0.53	1.73757E-12	AL731557.1	0.42	5.18726E-08
APOBEC3B-AS1	0.53	1.79986E-12	AC079336.3	0.42	5.21579E-08
MTATP6P4	0.53	1.91791E-12	FBXO36P1	0.42	5.26506E-08
AL132775.1	0.52	2.0323E-12	CEP192	0.42	5.26806E-08
LINC02074	0.52	2.36559E-12	MIR6775	0.42	5.28381E-08
KRTAP7-1	0.52	2.36685E-12	SNRPCP14	0.42	5.30425E-08
LINC02636	0.52	2.47796E-12	AL139010.1	0.42	5.52922E-08
ATP6V1G1P7	0.52	3.35344E-12	AL033523.1	0.42	5.54911E-08
AL157702.1	0.52	3.42124E-12	CLUHP3	0.42	5.61399E-08
CT55	0.52	3.79907E-12	F9	0.42	5.69591E-08
AC136431.2	0.52	3.84498E-12	AC121342.1	0.42	5.70032E-08
SOCS2P2	0.52	4.26118E-12	NLRP13	0.42	5.74883E-08
AL355516.1	0.52	4.27243E-12	MYO18B	0.42	5.84309E-08
AP001033.3	0.52	4.28548E-12	AC107926.1	0.42	5.9791E-08
AC005291.2	0.52	4.91387E-12	AL359736.1	0.42	6.07861E-08
AL353726.2	0.52	5.37697E-12	RN7SL79P	0.42	6.09998E-08
LINC01297	0.52	5.42385E-12	AC233702.1	0.42	6.25855E-08
BOK-AS1	0.52	5.47723E-12	COX20P2	0.42	6.26029E-08
AC183088.4	0.51	6.35232E-12	LINC01694	0.42	6.57356E-08
ART5	0.51	6.8005E-12	AC011626.1	0.42	6.9559E-08
MIR3972	0.51	6.94443E-12	AC023824.1	0.42	6.97164E-08
MPPE1P1	0.51	8.42152E-12	AC068790.2	0.42	7.06949E-08
MIR7975	0.51	8.73433E-12	RAB5CP1	0.42	7.10578E-08
RFPL3	0.51	8.8823E-12	AC073869.2	0.42	7.14685E-08
PPP1R2P6	0.51	9.03131E-12	ARL13A	0.41	7.32628E-08
AGGF1P5	0.51	9.26537E-12	AC106800.2	0.41	7.34786E-08
AQP11	0.51	9.50631E-12	CYP4F9P	0.41	7.34786E-08
RNASEH1P1	0.51	9.96326E-12	AC135050.6	0.41	7.55747E-08
PRR5-ARHGAP8	0.51	1.00849E-11	AC092058.1	0.41	7.60766E-08

AC006511.1	0.51	1.0586E-11	BCR	0.41	7.69076E-08
KRTAP4-1	0.51	1.19953E-11	L34079.2	0.41	7.74213E-08
SLC18A2	0.51	1.28025E-11	LINC01511	0.41	7.85077E-08
AL078602.1	0.51	1.28556E-11	AP001547.1	0.41	7.92558E-08
FUNDC2P3	0.51	1.32183E-11	NOC2LP1	0.41	8.09629E-08
LINC01861	0.51	1.33017E-11	AL590762.1	0.41	8.15098E-08
AL354674.1	0.51	1.38895E-11	AC107385.2	0.41	8.21145E-08
AC140479.4	0.51	1.38895E-11	RPL30P2	0.41	8.23242E-08
SMIM12P1	0.51	1.40251E-11	CCK	0.41	8.27271E-08
AC087222.1	0.51	1.42454E-11	AP001033.2	0.41	8.30454E-08
AC006504.4	0.51	1.49924E-11	LINC02084	0.41	8.56065E-08
ETNPPL	0.51	1.54307E-11	MAPRE1P3	0.41	8.80429E-08
RNU6-815P	0.51	1.61194E-11	MRPS21P9	0.41	9.27768E-08
AC009133.2	0.51	1.61194E-11	AL121949.1	0.41	9.30247E-08
RN7SL474P	0.51	1.61194E-11	MIRLET7I	0.41	9.49898E-08
AL390961.1	0.51	1.62284E-11	GSDME	0.41	1.01066E-07
LCN15	0.51	1.77538E-11	FO393415.1	0.41	1.01676E-07
LINC02432	0.50	1.84168E-11	CMTM6	0.41	1.05976E-07
AC007666.1	0.50	1.85408E-11	AC004771.1	0.41	1.068E-07
AC002351.1	0.50	1.85899E-11	AP001531.1	0.41	1.10565E-07
IGHV1OR21-1	0.50	2.11021E-11	AC020612.4	0.41	1.12598E-07
SRSF3P2	0.50	2.14786E-11	CST5	0.41	1.1279E-07
AC025277.1	0.50	2.77852E-11	AMMECR1L	0.41	1.15529E-07
AC244102.4	0.50	2.85431E-11	ANKRD20A4P	0.41	1.16896E-07
AC023906.5	0.50	2.92909E-11	AC002519.1	0.41	1.17941E-07
RNU6-1178P	0.50	2.98423E-11	LINC00895	0.41	1.19058E-07
CTAG1B	0.50	3.18457E-11	AL356747.1	0.41	1.1921E-07
MC3R	0.50	3.23874E-11	MIR1281	0.41	1.19582E-07
AL138686.1	0.50	3.55768E-11	INTS9-AS1	0.41	1.2531E-07
LINC01488	0.50	3.66205E-11	C10orf82	0.41	1.26789E-07
FAM30B	0.50	3.75602E-11	RNA5SP174	0.41	1.28792E-07
AL049693.1	0.50	3.787E-11	NRBF2P2	0.41	1.28923E-07
RNY1P3	0.50	3.787E-11	EAF1	0.41	1.29192E-07
AC117454.1	0.50	3.787E-11	AP001972.4	0.41	1.32458E-07
IGHVII-30-21	0.50	3.81692E-11	OR1J4	0.41	1.33082E-07
AL022329.3	0.50	3.97682E-11	AC006967.1	0.41	1.40892E-07
LDHAL6A	0.50	4.035E-11	AC087269.1	0.41	1.51959E-07
DPRXP7	0.50	4.05483E-11	AC027612.3	0.41	1.52519E-07
ANKRD20A19P	0.50	4.4853E-11	TRIM3	0.41	1.55326E-07
AJ239322.1	0.50	4.58522E-11	AL360091.1	0.41	1.56027E-07
PPP1R26P4	0.50	4.67216E-11	KLB	0.41	1.56665E-07
KCNH3	0.50	4.83361E-11	ARVCF	0.40	1.5783E-07

ZNF812P	0.50	4.98303E-11	AC000072.1	0.40	1.60312E-07
KRTAP11-1	0.49	5.22735E-11	AP000776.1	0.40	1.63332E-07
AC036108.2	0.49	6.68283E-11	AL451139.1	0.40	1.65037E-07
SNORD115-5	0.49	7.08232E-11	RARRES2P7	0.40	1.66609E-07
AC027369.4	0.49	7.08232E-11	AC127071.3	0.40	1.67201E-07
OXTR	0.49	7.35471E-11	AC104119.1	0.40	1.67933E-07
PASD1	0.49	7.35874E-11	PROZ	0.40	1.6994E-07
AC091646.1	0.49	7.56972E-11	RPS2P45	0.40	1.7008E-07
RXFP1	0.49	7.98967E-11	ANK2-AS1	0.40	1.70456E-07
ADD3-AS1	0.49	7.99198E-11	AC087269.2	0.40	1.70923E-07
AC011379.2	0.49	8.34289E-11	LINC00529	0.40	1.72305E-07
FAM245A	0.49	8.43416E-11	SPRING1	0.40	1.7424E-07
SCYGR9	0.49	8.70201E-11	BAIAP3	0.40	1.78983E-07
UCP1	0.49	8.87735E-11	MIR644A	0.40	1.81164E-07
DRAXINP1	0.49	8.97159E-11	CHRNA10	0.40	1.81841E-07
FAM245B	0.49	9.51019E-11	KRT12	0.40	1.82102E-07
RPS6P26	0.49	9.62815E-11	CLDN24	0.40	1.8242E-07
AC009102.3	0.49	1.05642E-10	IGBP1-AS1	0.40	1.8282E-07
RNA5SP341	0.49	1.06358E-10	MFF-DT	0.40	1.83108E-07
OR7C2	0.49	1.06777E-10	NSFP1	0.40	1.87353E-07
RNU6-1087P	0.49	1.06777E-10	AC236972.1	0.40	1.90773E-07
OR9L1P	0.49	1.06777E-10	KRTAP9-1	0.40	1.90773E-07
AL390123.1	0.49	1.06777E-10	RNU6-600P	0.40	1.94169E-07
NBPF21P	0.49	1.08676E-10	AL133243.2	0.40	1.96559E-07
AL450311.1	0.49	1.13392E-10	BMP6P1	0.40	1.98542E-07
RPL12P39	0.49	1.23993E-10	AC004022.2	0.40	1.98985E-07
AC113143.2	0.49	1.29936E-10	RPL35AP31	0.40	1.9992E-07
AL157414.2	0.49	1.30845E-10	GTF3C6P3	0.40	2.02046E-07
ARAFP1	0.49	1.30845E-10	AL035691.1	0.40	2.04562E-07
AC018712.2	0.49	1.30845E-10	AL390961.3	0.40	2.06321E-07
RPSAP68	0.49	1.30845E-10	MIR4443	0.40	2.06477E-07
AC134980.1	0.49	1.38333E-10	AL136418.1	0.40	2.06703E-07
AC087863.1	0.48	1.60126E-10	RNU6-705P	0.40	2.1379E-07
CEND1P1	0.48	1.60126E-10	AC015982.1	0.40	2.18563E-07
NUTF2P8	0.48	1.60126E-10	PFN1P10	0.40	2.25003E-07
AC025678.2	0.48	1.72302E-10	AC004232.2	0.40	2.30066E-07
OR1L1	0.48	1.78356E-10	RPL6P30	0.40	2.32029E-07
RNA5SP290	0.48	1.91214E-10	VN1R91P	0.40	2.34208E-07
UBE2V1P11	0.48	1.957E-10	AC008080.1	0.40	2.34431E-07
AC117522.1	0.48	1.957E-10	KMT5B	0.40	2.35101E-07
KATNBL1P4	0.48	1.957E-10	ARHGEF33	0.40	2.35893E-07
AC100810.2	0.48	1.957E-10	AC131235.1	0.40	2.36847E-07

MTND6P19	0.48	1.957E-10	PRAP1	0.40	2.37059E-07
PRR20G	0.48	2.14599E-10	AC097015.1	0.40	2.40522E-07
AC087392.3	0.48	2.35739E-10	AC008443.6	0.40	2.47207E-07
AC112243.1	0.48	2.60192E-10	AC023908.1	0.40	2.54218E-07
ZPLD2P	0.48	2.637E-10	AL139008.2	0.40	2.56285E-07
AC116099.1	0.48	2.64597E-10	RPSAP37	0.40	2.65074E-07
AC008443.2	0.48	2.64937E-10	OR8S21P	0.40	2.65074E-07
FAM9B	0.48	2.67154E-10	AL445675.1	0.40	2.6548E-07
AC021683.6	0.48	2.69873E-10	AC008083.1	0.40	2.66939E-07
AL391358.1	0.48	2.99658E-10	AL132765.1	0.40	2.68743E-07
AC007342.4	0.48	2.99886E-10	FAM95B1	0.40	2.73853E-07
ELOCP21	0.48	3.45616E-10	AP002812.5	0.40	2.74579E-07
AC004875.1	0.48	3.48049E-10	AL133493.2	0.40	2.75417E-07
AC010601.1	0.48	3.49833E-10	AC104071.1	0.40	2.83963E-07
OR52M1	0.48	3.50053E-10	AC008738.6	0.40	2.84721E-07
AC134978.1	0.47	3.85112E-10	RPL23AP15	0.40	2.89886E-07
AP001804.1	0.47	3.90742E-10	MTCO1P54	0.40	2.90778E-07
LINC02181	0.47	4.01627E-10	MTDHP2	0.40	2.92571E-07
ABCA9-AS1	0.47	4.2265E-10	AC019257.2	0.40	3.02178E-07
AP003559.1	0.47	4.42463E-10	EXOC5P1	0.40	3.04097E-07
GOLGA8M	0.47	4.55371E-10	PGAM1P11	0.40	3.12389E-07
RN7SKP169	0.47	4.69699E-10	ARHGAP28	0.40	3.13931E-07
BX072579.2	0.47	4.84168E-10	FAM30C	0.40	3.25863E-07
OR7E145P	0.47	5.10522E-10	DPYS	0.40	3.27978E-07
AL109897.1	0.47	5.1855E-10	AL365203.1	0.40	3.28291E-07
AC018697.1	0.47	5.35282E-10	LINC01287	0.40	3.2947E-07
TAS1R2	0.47	5.50799E-10	PLCD1	0.40	3.32018E-07
LINC01524	0.47	5.82417E-10			
CCDC144NL-AS1	0.47	6.08038E-10			
OR56A3	0.47	6.09016E-10			
SLC7A10	0.47	6.28858E-10			

Table S7. GO annotation of genes positively associated with LINC01087 expression in BC, ESCA, OV, STAD and TGCT.

GO domain	GO term description	GO term ID	N. of genes	P-value adj	Gene symbol
BP	synaptic transmission. GABAergic	GO:0051932	9	0.008	GABRG1 (ESCA), NLGN1 (ESCA), GABRG2 (ESCA),OXTR (ESCA, STAD),GABRA2 (ESCA),BAIAP3 (ESCA, STAD), SYN3 (STAD), RAC3 (TGCT),GABRA6 (TGCT)
CC	synaptic membrane	GO:0097060	22	0.009	SLITRK1 (ESCA), GABRG1 (ESCA), GRIN3A (ESCA), GABBR2 (ESCA), NLGN1 (ESCA), GRIA2 (ESCA), GRIA4 (ESCA), DNM3 (ESCA), GLRB (ESCA), ERBB4 (ESCA), CDH10 (ESCA), GABRG2 (ESCA, STAD), KCNC3 (ESCA), GABRA2 (ESCA), GRIN1 (ESCA), GSG1L (ESCA), CHRNE (ESCA), CDH8 (STAD), SNCAIP (STAD), CNTN5 (STAD), GABRA6 (TGCT), CHRNA10 (TGCT)
CC	postsynaptic membrane	GO:0045211	19	0.009	SLITRK1 (ESCA), GABRG1 (ESCA), GRIN3A (ESCA), GABBR2 (ESCA), NLGN1 (ESCA), GRIA2 (ESCA), GRIA4 (ESCA), DNM3 (ESCA), GLRB (ESCA), ERBB4 (ESCA), CDH10 (ESCA), GABRG2 (ESCA, STAD), KCNC3 (ESCA), GABRA2 (ESCA), GRIN1 (ESCA), GSG1L (ESCA), CHRNE (ESCA), GABRA6 (TGCT), CHRNA10 (TGCT)
CC	DNA packaging complex	GO:0044815	13	0.032	H2BU1 (ESCA), H4C5 (ESCA), H2AC7 (ESCA), STAG3 (ESCA), H2BC15 (ESCA), H2AW (ESCA), RAD21L1 (ESCA), H2AB3 (ESCA), SS18L1 (ESCA), H2BC9 (ESCA), BCL11A (STAD), H2BW1 (STAD), H2BC19P (STAD)
CC	postsynaptic specialization membrane	GO:0099634	10	0.020	SLITRK1 (ESCA), GRIN3A (ESCA), NLGN1 (ESCA), GRIA2 (ESCA), ERBB4 (ESCA), CDH10 (ESCA), GABRG2 (ESCA, STAD), GABRA2 (ESCA), GSG1L (ESCA), CHRNA10 (TGCT)
CC	integral component of postsynaptic specialization membrane	GO:0099060	9	0.009	SLITRK1(ESCA), GRIN3A(ESCA), NLGN1(ESCA), ERBB4(ESCA), CDH10(ESCA), GABRG2(ESCA, STAD), GABRA2(ESCA), GSG1L (ESCA), CHRNA10 (TGCT)
CC	intrinsic component of postsynaptic specialization membrane	GO:0098948	9	0.009	SLITRK1 (ESCA), GRIN3A (ESCA), NLGN1 (ESCA), ERBB4 (ESCA), CDH10 (ESCA), GABRG2 (ESCA, STAD), GABRA2 (ESCA), GSG1L (ESCA), CHRNA10 (TGCT)
CC	GABA-ergic synapse	GO:0098982	8	0.017	SLITRK1 (ESCA), GLRB (ESCA), ERBB4 (ESCA), CDH10 (ESCA), GABRG2 (ESCA, STAD), GABRA2 (ESCA), EFNA5 (ESCA), CNTN5 (STAD)
CC	synaptonemal complex	GO:0000795	6	0.019	SYCE1 (ESCA), STAG3 (ESCA), RAD21L1 (ESCA), SYCP2L (STAD), FAM9B (TGCT), FAM9A (TGCT)
CC	synaptonemal structure	GO:0099086	6	0.019	SYCE1 (ESCA), STAG3 (ESCA), RAD21L1 (ESCA), SYCP2L (STAD), FAM9B (TGCT), FAM9A (TGCT)
CC	chloride channel complex	GO:0034707	6	0.043	GABRG1 (ESCA), GLRB (ESCA), GABRG2 (ESCA, STAD), GABRA2 (ESCA), CLCN1 (OV), GABRA6 (ESCA)

CC	GABA receptor complex	GO:1902710	5	0.009	GABRG1 (ESCA), GABBR2 (ESCA), GABRG2 (ESCA, STAD), GABRA2 (ESCA), GABRA6 (TGCT)
CC	synaptic cleft	GO:0043083	4	0.030	NLGN1 (ESCA), DNM3 (ESCA), GRIN1 (ESCA), CDH8 (STAD)
CC	GABA-A receptor complex	GO:1902711	4	0.032	GABRG1 (ESCA), GABRG2 (ESCA, STAD), GABRA2 (ESCA), GABRA6 (TGCT)
MF	channel activity	GO:0015267	23	0.027	GABRG1 (ESCA), GRIN3A (ESCA), GRIA2 (ESCA), GRIA4 (ESCA), GLRB (ESCA), PANX3 (ESCA), KCNV1 (ESCA), KCNQ2 (ESCA), GABRG2 (ESCA, STAD), P2RX2 (ESCA), KCNH5 (ESCA), KCNN1 (ESCA), KCNC3 (ESCA), GABRA2 (ESCA), GRIN1 (ESCA), GJD2 (ESCA), CHRNE (ESCA), CLCN1 (OV), GJA8 (OV), AQP11 (TGCT), KCNH3 (TGCT), GABRA6 (TGCT), CHRNA10 (TGCT)
MF	passive transmembrane transporter activity	GO:0022803	23	0.027	GABRG1 (ESCA), GRIN3A (ESCA), GRIA2 (ESCA), GRIA4 (ESCA), GLRB (ESCA), PANX3 (ESCA), KCNV1 (ESCA), KCNQ2 (ESCA), GABRG2 (ESCA, STAD), P2RX2 (ESCA), KCNH5 (ESCA), KCNN1 (ESCA), KCNC3 (ESCA), GABRA2 (ESCA), GRIN1 (ESCA), GJD2 (ESCA), CHRNE (ESCA), CLCN1 (OV), GJA8 (OV), AQP11 (TGCT), KCNH3 (TGCT), GABRA6 (TGCT), CHRNA10 (TGCT)
MF	gated channel activity	GO:0022836	19	0.012	GABRG1 (ESCA), GRIN3A (ESCA), GRIA2 (ESCA), GRIA4 (ESCA), GLRB (ESCA), KCNV1 (ESCA), KCNQ2 (ESCA), GABRG2 (ESCA, STAD), P2RX2 (ESCA), KCNH5 (ESCA), KCNN1 (ESCA), KCNC3 (ESCA), GABRA2 (ESCA), GRIN1 (ESCA), CHRNE (ESCA), CLCN1 (OV), KCNH3 (TGCT), GABRA6 (TGCT), CHRNA10 (TGCT)
MF	ligand-gated ion channel activity	GO:0015276	13	0.002	GABRG1 (ESCA), GRIN3A (ESCA), GRIA2 (ESCA), GRIA4 (ESCA), GLRB (ESCA), GABRG2 (ESCA, STAD), P2RX2 (ESCA), KCNN1 (ESCA), GABRA2 (ESCA), GRIN1 (ESCA), CHRNE (ESCA) GABRA6 (TGCT), CHRNA10 (TGCT)
MF	ligand-gated channel activity	GO:0022834	13	0.002	GABRG1, GRIN3A, GRIA2, GRIA4, GLRB, GABRG2, P2RX2, KCNN1, GABRA2, GRIN1, CHRNE, GABRA6, CHRNA10
MF	extracellular ligand-gated ion channel activity	GO:0005230	12	1.82E-05	GABRG1 (ESCA), GRIN3A (ESCA), GRIA2 (ESCA), GRIA4 (ESCA), GLRB (ESCA), GABRG2 (ESCA, STAD), P2RX2 (ESCA), GABRA2 (ESCA), GRIN1 (ESCA), CHRNE (ESCA), GABRA6 (TGCT), CHRNA10 (TGCT)
MF	transmitter-gated ion channel activity	GO:0022824	11	1.82E-05	GABRG1 (ESCA), GRIN3A(ESCA), GRIA2(ESCA), GRIA4(ESCA), GLRB (ESCA), GABRG2 (ESCA, STAD), GABRA2(ESCA), GRIN1 (ESCA), CHRNE (ESCA), GABRA6 (TGCT), CHRNA10 (TGCT)
MF	transmitter-gated channel activity	GO:0022835	11	1.82E-05	GABRG1 (ESCA), GRIN3A (ESCA), GRIA2 (ESCA), GRIA4 (ESCA), GLRB (ESCA), GABRG2 (ESCA, STAD), GABRA2 (ESCA), GRIN1 (ESCA), CHRNE (ESCA), GABRA6 (TGCT), CHRNA10 (TGCT)

MF	neurotransmitter receptor activity	GO:0030594	11	0.003	GABRG1 (ESCA), GRIN3A (ESCA), GRIA2 (ESCA), GRIA4 (ESCA), GLRB (ESCA), GABRG2 (ESCA, STAD), GABRA2 (ESCA), GRIN1 (ESCA), CHRNE (ESCA), GABRA6 (TGCT), CHRNA10 (TGCT)
MF	peptide receptor activity	GO:0001653	11	0.026	NTSR2 (ESCA), PROKR2 (ESCA), OXTR (ESCA, TGCT), PTH2R (ESCA), UTS2R (ESCA), GPR37 (ESCA), GPR83 (OV), VIPR2 (OV), GUCY2D (STAD), MC3R (TGCT), RXFP1 (TGCT)
MF	odorant binding	GO:0005549	9	0.04348	OR13G1 (BRCA), OR8D1 (ESCA), OR8A1 (ESCA), OR8B12 (ESCA), OR8D2 (ESCA), OR8B2 (ESCA), OR8G1 (ESCA), OR8B3 (ESCA), OR8G3P (ESCA)
MF	transmitter-gated ion channel activity involved in regulation of postsynaptic membrane potential	GO:1904315	8	0.001	GABRG1 (ESCA), GRIN3A (ESCA), GLRB (ESCA), GABRG2 (ESCA), GABRA2 (ESCA, STAD), CHRNE (ESCA), GABRA6 (TGCT), CHRNA10 (TGCT)
MF	neurotransmitter receptor activity involved in regulation of postsynaptic membrane potential	GO:0099529	8	0.001	GABRG1 (ESCA), GRIN3A (ESCA), GLRB (ESCA), GABRG2 (ESCA, STAD), GABRA2 (ESCA), CHRNE (ESCA), GABRA6 (TGCT), CHRNA10 (TGCT)
MF	postsynaptic neurotransmitter receptor activity	GO:0098960	8	0.005	GABRG1 (ESCA), GRIN3A (ESCA), GLRB (ESCA), GABRG2 (ESCA, STAD), GABRA2 (ESCA), CHRNE (ESCA), GABRA6 (TGCT), CHRNA10 (TGCT)
MF	inhibitory extracellular ligand-gated ion channel activity	GO:0005237	5	0.001	GABRG1 (ESCA), GLRB (ESCA), GABRG2 (ESCA, STAD), GABRA2 (ESCA), GABRA6 (TGCT)
MF	ligand-gated anion channel activity	GO:0099095	5	0.002	GABRG1 (ESCA), GLRB (ESCA), GABRG2 (ESCA, STAD), GABRA2 (ESCA), GABRA6 (TGCT)
MF	GABA receptor activity	GO:0016917	5	0.005	GABRG1, GABBR2, GABRG2, GABRA2, GABRA6
MF	benzodiazepine receptor activity	GO:0008503	4	0.004	GABRG1 (ESCA), GABRG2 (ESCA, STAD), GABRA2 (ESCA), GABRA6 (TGCT)
MF	GABA-gated chloride ion channel activity	GO:0022851	4	0.006	GABRG1 (ESCA), GABRG2 (ESCA, STAD), GABRA2 (ESCA), GABRA6 (TGCT)
MF	GABA-A receptor activity	GO:0004890	4	0.026	GABRG1 (ESCA), GABRG2 (ESCA, STAD), GABRA2 (ESCA), GABRA6 (TGCT)
MF	ionotropic glutamate receptor activity	GO:0004970	4	0.026	GRIN3A (ESCA), GRIA2 (ESCA), GRIA4 (ESCA), GRIN1 (ESCA)

Tumors associated with enriched genes in each GO term are reported in brackets. Abbreviations: adj. adjusted p-value; BC, breast cancer; BP; biological process; CC, cellular component; ESCA, esophageal carcinoma; GABA: gamma-amminobutyric acid; GO, gene ontology; MF, molecular function; OV, ovarian cancer; STAD, stomach adenomcarcinoma; TGCT, testicular germ cell cancers.

Table S8. KEGG pathways associated with LINC01087 expression in BC, ESCA, OV, STAD and TGCT.

Pathway ID	Pathway description	N. of genes	P-value adj	Gene symbol
hsa04080	Neuroactive ligand-receptor interaction	28	3.48159E-07	GNRHR (BC), TAC3 (ESCA), GABRG1 (ESCA), GRIN3A (ESCA), GABBR2 (ESCA), PTH2 (ESCA), GRIA2 (ESCA), GRIA4 (ESCA), GLRB (ESCA), NTSR2 (ESCA), GABRG2 (ESCA, STAD), P2RX2 (ESCA), OXTR (ESCA, STAD), GABRA2 (ESCA), GRIN1 (ESCA), PTH2R (ESCA), UTS2R (ESCA), CHRNE (ESCA), CALCA (OV), PATE2 (OV), GPR83 (OV), VIPR2 (OV), AVP (STAD), MC3R (TGCT), RXFP1 (TGCT), GABRA6 (TGCT), CCK (TGCT), CHRNA10 (TGCT)
hsa05034	Alcoholism	12	0.02821	GNG13 (ESCA), GRIN3A (ESCA), H2BU1 (ESCA), H4C5 (ESCA), H2AC7 (ESCA), GRIN1 (ESCA), H2BC15 (ESCA), H2AW (ESCA), H2AB3 (ESCA), H2BC9 (ESCA), H2BW1 (STAD), SLC18A2(TGCT)
hsa04742	Taste transduction	9	0.00501	TAS2R20 (BC), TAS2R4 (BC), GNG13(ESCA), GABBR2(ESCA), P2RX2(ESCA), GABRA2(ESCA), TAS2R14(ESCA), TAS1R2 (TGCT), GABRA6 (TGCT)
hsa05033	Nicotine addiction	8	0.00014	GABRG1 (ESCA), GRIN3A (ESCA), GRIA2 (ESCA), GRIA4 (ESCA), GABRG2 (ESCA, STAD), GABRA2 (ESCA), GRIN1 (ESCA), GABRA6 (TGCT)

Tumors associated with enriched genes in each KEGG pathway are reported in brackets. Abbreviations: adj. adjusted p-value; BC, breast cancer; ESCA, esophageal carcinoma; KEGG, Kyoto encyclopedia of genes and genomes; OV, ovarian cancer; STAD, stomach adenocarcinoma; TGCT, testicular germ cell cancers.

Table S9. Prediction of subcellular localization of LINC0187 using "lncLocator" database.

**Subcellular locations**	**score**
Cytoplasm	0.76
Cytosol	0.16
Ribosome	0.04
Nucleus	0.03

Table S10. List of the 64 miRNAs significantly correlated with LINC01087 expression in BC, ESCA, OV, STAD and TGCT in TCGA datasets (Analysis 2).

BC			ESCA			OV			STAD			TGCT		
**miRNA symbol**	**R value**	**P-value**	**miRNA symbol**	**R value**	**P-value**	**miRNA symbol**	**R v alue**	**P-value**	**miRNA symbol**	**R value**	**P-value**	**miRNA symbol**	**R value**	**P-value**
**MIR1252**	**0.67**	**4.7256E-143**	**MIR5692B**	**0.72**	**3.69344E-27**	MIR196A2	0.52	2.28855E-27	MIR557	0.61	1.92265E-39	**MIR3199-1**	**0.70**	**1.12728E-24**
**MIR7157**	**0.64**	**2.8214E-128**	**MIR4666A**	**0.70**	**2.10511E-25**	MIR4255	0.47	6.15206E-22	MIR1285-1	0.52	9.48333E-28	MIR1238	0.57	5.7326E-15
MIR4460	0.63	6.6005E-126	MIR1915HG	0.63	1.05664E-19	MIR138-2	0.41	8.98595E-17	MIR653	0.52	7.27084E-27	MIR3972	0.51	6.94443E-12
MIR1537	0.58	4.9776E-103	MIR663AHG	0.60	5.22915E-17				MIR647	0.50	1.5159E-25	MIR7975	0.51	8.73433E-12
MIR554	0.56	2.90578E-93	MIR548F1	0.50	6.64905E-12				MIR4453HG	0.48	9.30321E-23	MIR6779	0.45	3.01021E-09
MIR569	0.55	9.277E-91	MIR3153	0.41	6.22725E-08				MIR5582	0.45	9.89151E-20	MIR3164	0.45	3.63077E-09
MIR3173	0.54	7.2137E-85							MIR5087	0.43	2.09532E-18	MIR4433B	0.44	1.0498E-08
MIR545	0.53	1.00071E-82							MIR2052HG	0.43	2.47984E-18	MIR4740	0.43	1.54195E-08
MIR4505	0.51	3.97646E-75							MIR718	0.43	5.77886E-18	MIR4632	0.43	2.31714E-08
MIR920	0.51	1.46709E-74							MIR548AR	0.42	7.64309E-18	MIR6775	0.42	5.28381E-08
MIR3671	0.51	3.62973E-74							MIR1305	0.41	6.42455E-17	MIRLET7I	0.41	9.49898E-08
MIR553	0.48	9.56536E-65										MIR1281	0.41	1.19582E-07
MIR3134	0.47	2.83038E-63										MIR644A	0.40	1.81164E-07
MIR509-1	0.47	2.60502E-62										MIR4443	0.40	2.06477E-07
MIR606	0.47	3.47304E-61												
MIR4526	0.47	6.48118E-61												
MIR548AX	0.46	7.97398E-59												
MIR346	0.46	1.4667E-58												
MIR3936HG	0.45	6.44436E-58												
MIR4774	0.45	1.06949E-56												
MIR582	0.45	1.43479E-56												
MIR3174	0.44	1.07175E-54												
MIR3170	0.44	3.95551E-53												
MIR5586	0.43	1.25373E-51												

MIR376A2	0.43	3.2875E-51
MIR626	0.42	7.24075E-50
MIR1284	0.41	7.15407E-47
MIR4796	0.41	3.20014E-45
MIR491	0.40	1.46935E-44
MIR651	0.40	4.61001E-44

miRNAs showing the top 5 strongest correlation value (R) across the tumor types are highlighted in bold. Abbreviations: BC, breast cancer; ESCA, esophageal carcinoma; OV, ovarian cancer; STAD, stomach adenocarcinoma; TGCT, testicular germ cell cancers.

Table S11. List of the 31 mRNA targets of LINC01087-related miRNAs that overlapped between Analysis 1 and Analysis 2.

ACVR2B
ARIH2OS
**C8orf37**
**EFNA5**
FAM169B
FAM216A
FTCD
GABBR2
GSG1L
HES7
**HOOK3**
HOXD10
**IQCG**
KBTBD13
LVRN
**MYOZ3**
**PCP4L1**
PDE6B
PKHD1
**PLAG1**
**POLI**
RBM48
RGL2
RTBDN
SELENOS
**SLC2A3**
**SNRPA1**
UFL1
VPS50
ZC3H11A
ZNF410

Target genes shared between the Analyses 2 and 3 are highlighted in bold.

Table S12. List of the 20 miRNA identified to interact with LINC01087 in the DIANA-LncBASE repository (Analysis 3).

hsa-let-7a-5p
hsa-let-7d-5p
hsa-let-7e-5p
hsa-miR-148b-5p
hsa-miR-152-3p
hsa-miR-181a-5p
hsa-miR-181b-5p
hsa-miR-181d-5p
hsa-miR-197-3p
hsa-miR-19a-3p
hsa-miR-19b-3p
hsa-miR-21-5p
hsa-miR-25-3p
hsa-miR-32-5p
hsa-miR-34a-5p
hsa-miR-423-5p
hsa-miR-7-5p
hsa-miR-92a-3p
hsa-miR-92b-3p
hsa-miR-98-5p

Table S13. List of the 68 mRNA targets of LINC01087-related miRNAs that overlapped between Analysis 1 and Analysis 3.

ADAMTS17
ADGRL2
AMMECR1L
ANKIB1
ANKRD36
ARID3A
ARRDC4
BCL11A
BCR
BMF
BMP3
BTBD10
C10orf88
C17orf100
C2orf68
**C8orf37**
CDH18
CMTM6
CST5
CTCFL
CYP51A1
DENND2C
DSCR8
EDIL3
**EFNA5**
ERBB4
FIGN
GATA3
GPR37
GPR55
GPR83
GRIA2
HABP4
HAPLN1
**HOOK3**
HOXC12
IGF2BP1
**IQCG**
KLB

KRIT1
KRTAP11-1
LRRC28
LRRN3
MAGEA6
MATR3
MST1
**MYOZ3**
NAT8L
NUTM1
OXTR
PATE2
PCDH10
**PCP4L1**
PDGFC
**PLAG1**
**POLI**
POTEG
PRR3
PRR5-ARHGAP8
SALL3
SALL4
SLC11A2
SLC2A11
**SLC2A3**
**SNRPA1**
ZFR2
ZNF280B
ZNF780A

Target genes shared between the Analyses 2 and 3 are highlighted in bold.

Supplementary Figures

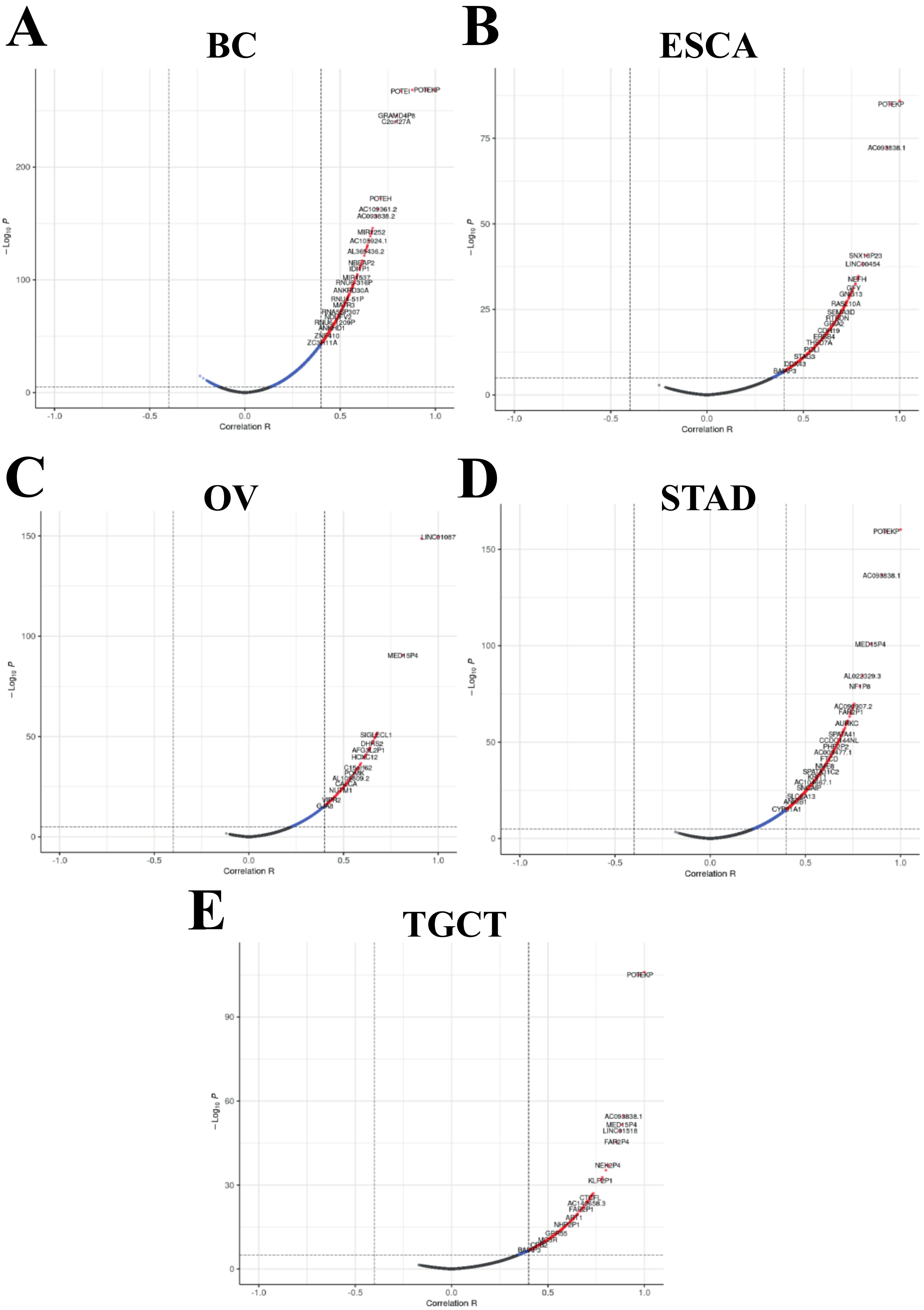

Figure S1. Differentially expressed genes correlated with LINC01087 expression in several tissue types extracted from TCGA. Volcano plots showing both significant (Spearman correlation test, R> 0.4. *p*< 0.05, in red) and non-significant (Spearman correlation test, R< 0.4. *p*> 0.05, in dark and light grey) correlations between LINC01087 expression and transcripts in BC (**A**), ESCA (**B**), OV (**C**), STAD (**D**), and TGCT (**E**). Genes showing cut-off criteria of |R|< 0.4 and *p*< 0.05 value are highlighted in blue. The expression values are reported as log2FC. Corresponding data of the statistical analyses are displayed in **Supplementary Table S2**-**S6**. Abbreviations: BC, breast cancer; ESCA, esophageal carcinoma**;** OV, ovarian cancer; STAD, stomach adenocarcinoma; TCGA, the cancer genome atlas; TGCT, testicular germ cell tumours.
